# Supplementary material for: A combinatorial code of neurexin-3 alternative splicing controls inhibitory synapses via a trans-synaptic dystroglycan signaling loop
Source: Nat Commun. 2023 Mar 30;14:1771. doi: 10.1038/s41467-023-36872-8 (PMC10063607; doi:10.1038/s41467-023-36872-8)
Supplement: Supplementary file 1 — Supplementary Information [file 41467_2023_36872_MOESM1_ESM.docx]

**SUPPLEMETARY INFORMATION**

**A combinatorial code of neurexin-3 alternative splicing controls inhibitory synapses via a trans-synaptic dystroglycan signaling loop**

Justin H. Trotter, Cosmos Yuqi Wang, Peng Zhou, George Nakahara, and Thomas C. Südhof


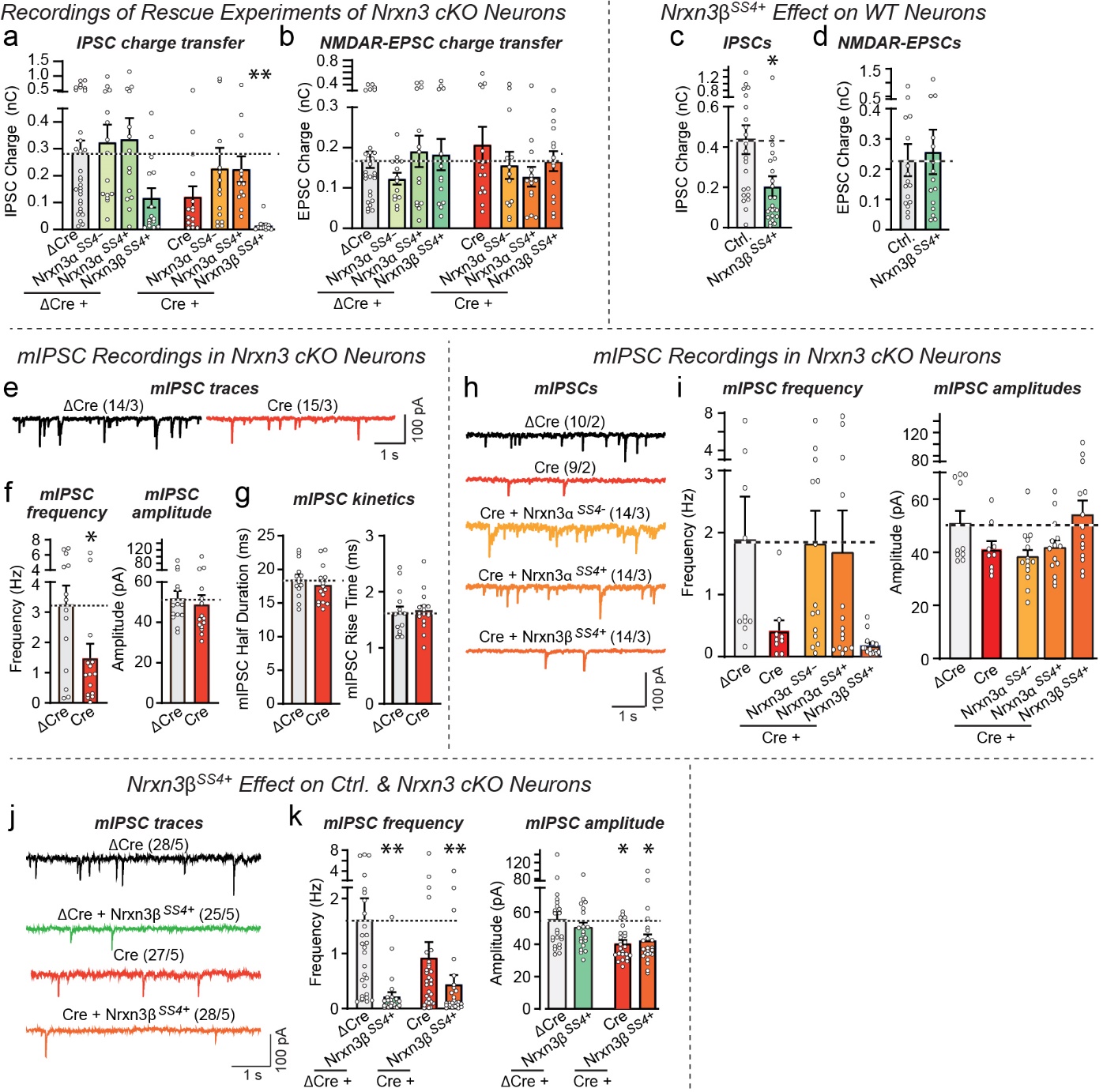


Figure S1: **Further analyses confirm that Nrxn3α-SS4+ and Nrxn3α-SS4- splice variants both rescue impaired GC🡪MC inhibitory synaptic transmission in *Nrxn3*-deficient cultured OB neurons, whereas Nrxn3β-SS4+ suppresses synaptic transmission at these synapses.**

**a** & **b**. Quantification of evoked IPSC charge transfer confirms that the conditional *Nrxn3* deletion impairs evoked IPSCs in a manner that can be rescued by Nrxn3α but not Nrxn3β (a), but that the deletion of *Nrxn3* has no effect on evoked NMDAR-EPSCs (b). Data are from the same experiments as shown in Figure 1b, 1c, 1f & 1g.

**c** & **d**. Quantification of the synaptic charge transfer during evoked IPSCs confirms that the expression of Nrxn3β-SS4+ in wild-type neurons severely impairs evoked IPSCs in a dominant-negative fashion (c) but has no effect on evoked NMDAR-EPSCs (d). Data are from the same experiments as shown in Figure 1d, 1e, 1h & 1i.

**e**-**g**. Conditional deletion of *Nrxn3* in cultured OB neurons decreases the frequency of spontaneous mIPSCs by ~60% without significantly altering the amplitude or kinetics of mIPSCs (e, sample traces; f, summary graphs of the mIPSC frequency and amplitudes; g, summary graphs of the mIPSC decay and rise times).

**h** & **i**. Conditional deletion of *Nrxn3* in cultured OB neurons decreases spontaneous mIPSCs; this decrease is rescued by Nrxn3α but not Nrxn3β (h, sample traces; i, summary graphs of mIPSC frequency and amplitudes).

**j** & **k**. Independent experiments documenting that expression of Nrxn3β-SS4+ with an insert at SS4 suppresses the mIPSC frequency in both wild-type and *Nrxn3*-deficient cultured OB neurons (j, sample traces; k, summary graphs of the mIPSC frequency and amplitudes).

Numerical data are means ± SEM; n’s (cells/experiments) are indicated above the sample traces and apply to all graphs in an experimental series. Statistical analyses were performed with a one-way analysis of variance (ANOVA) with Dunnett’s multiple comparison test (a, b, i, and k) or a two-tailed unpaired *t* test (c, d, f, and g), with * = p<0.05, ** = p<0.01, and *** = p<0.001. Source data and statistical results are provided within the Source Data file.


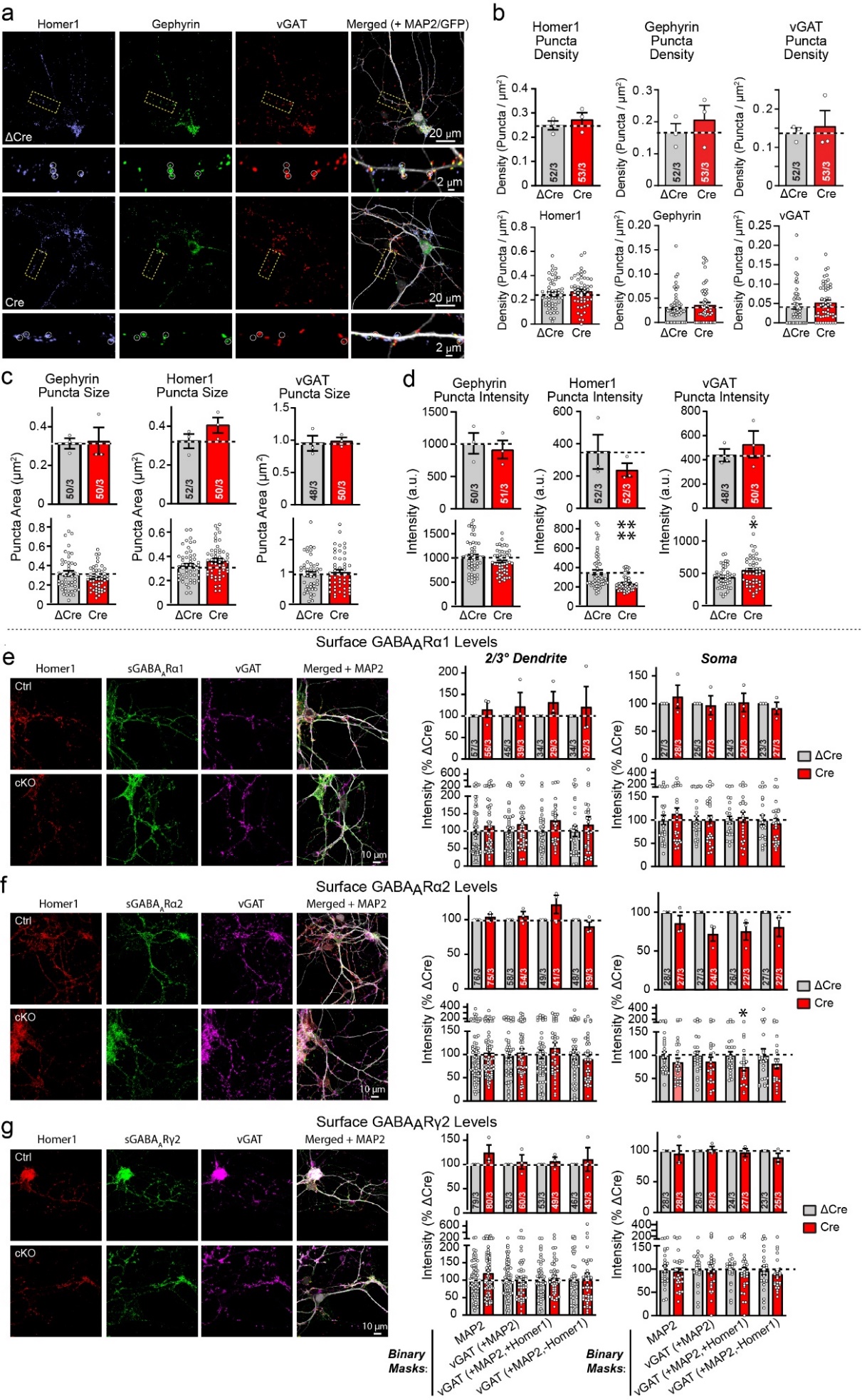


Figure S2: **The *Nrxn3* deletion does not alter the inhibitory synapse density in cultured OB neurons (a-d), nor does it alter the surface levels of postsynaptic GABA_A_-receptors (e-g)**

**a.** Further representative images related to the data of Fig. 1j & 1k.

**b.** Additional quantifications of immunocytochemical analyses of cultured OB neurons in which larger mitral/tufted cells form dendrodendritic synapses with smaller granule cells and other interneurons, confirming that the synapse density is not altered by deletion of *Nrxn3*. Summary graphs show quantifications of the density of Homer1+ excitatory synapses (left, Homer1-positive), gephyrin+ inhibitory synapses (middle, gephyrin-positive), and vGAT+ inhibitory synapses (right vGAT-positive). Data are shown as averaged per experiment (top) or per ROI (bottom).

**c & d.** The *Nrxn3* deletion has no effect on the size (c) or intensity (d) of synaptic puncta (left, gephyrin; middle, Homer1; right, vGAT). Data are shown as averaged per independent culture (top) or per ROI (bottom).

**e-g**. The surface expression of several GABA_A_R subunits, namely GABA_A_Rα1 (e), GABA_A_Rα2 (f) and GABA_A_Rγ2 (g) is not altered by the *Nrxn3* deletion. For each set of data, representative images are shown on the left and summary graphs on the right. Receptor levels were measured by staining non-permeabilized neurons with antibodies to the indicated receptors, and were quantified at secondary and tertiary dendrites (left summary graphs) and over the soma (right summary graphs). Receptors levels monitored as background-subtracted fluorescence intensity are quantified in areas defined by binary masks of other stains including the dendrite and various combinations of synaptic markers. Data are shown as averaged values per experiment (top) or per ROI (bottom) for the reasons explained in the legend to panel b.

Numerical data are means ± SEM; n’s (images/experiments) are indicated in the summary graph bars and apply to all graphs in an experimental series. Statistical analyses were performed using a two-tailed unpaired *t* test, with * = p<0.05 and **** = p<0.0001. Source data and statistical results are provided within the Source Data file.


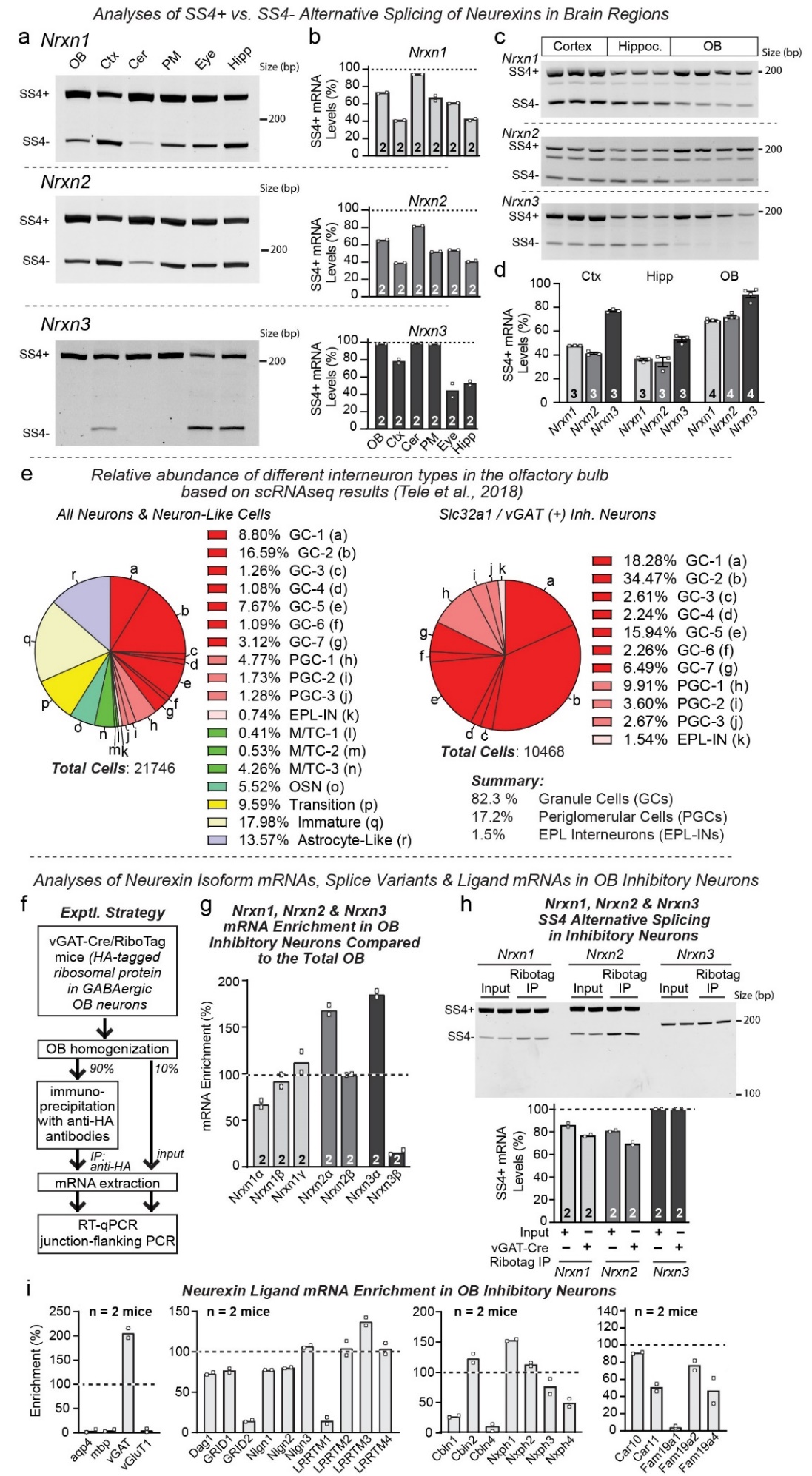


Figure S3: **Expression patterns of neurexin mRNAs and their SS4 splice variants in different brain regions (a-d), distribution of cell types in the OB based on scRNA-seq (e), RT-PCR analyses of the *Nrxn1-3* mRNA levels and SS4 alternative splicing in OB (f-i) and of the mRNA levels of neurexin ligands (j).**

**a & b**. *Nrxn1*-*Nrxn3* mRNAs exhibit brain region-specific differences in SS4 alternative splicing inclusion (a, representative gels; b, summary graphs of the SS4+ RNA as percent of the total). Abbreviations used: OB, olfactory bulb; Ctx, cortex; PM, pons and medulla; Cer, cerebellum; Hipp, hippocampus.

**c & d**. *Nrxn1*-*Nrxn3* mRNAs differ in SS4 alternative splicing when analyzed with RNA from dissociated cortical (Cx), hippocampal (Hipp), and olfactory bulb (OB) cultures (c, representative images; d, summary graphs).

**e**. Inhibitory granule cells make up ~82% of total *Slc32a1*-expressing inhibitory neurons. The distribution of neuron and neuron-like cell types (left) and inhibitory neurons (right) in the adult olfactory bulb were generated using scRNA-seq data from Tele et al., 2018.

**f.** Experimental strategy to isolate and analyze translating mRNAs from OB inhibitory neurons.

**g**. Nrxn2α and Nrxn3α mRNAs are enriched in OB inhibitory neurons. Data show relative abundance of indicated mRNAs in inhibitory neurons normalized to the total mRNAs.

**h** & **i**. Nearly all of Nrxn3 mRNAs are expressed as SS4+ variants in OB inhibitory neurons (h, representative image of junction-flanking PCR analysis of total OB mRNA (input) and vGAT-Cre/Ribotag-isolated OB inhibitory neuron mRNA [immunoprecipitation (IP): anti-HA; i, quantification of the relative abundance of SS4+ mRNAs).

**j.** Validation of the purification of mRNAs from OB inhibitory neurons by quantifications of cell-specific markers (left summary graph; aqp, aquaporin for astrocytes; mbp, myelin basic protein for oligodendrocytes; vGluT1 and vGAT for excitatory and inhibitory neurons, respectively), and measurements of neurexin ligand mRNAs (middle and right summary graphs).

Numerical data are means ± SEM; n’s (cells/experiments) are indicated in the summary graph bars and apply to all graphs in an experimental series. Source data and statistical results are provided within the Source Data file. Scanned gels are p­rovided in the Supplementary Information File.


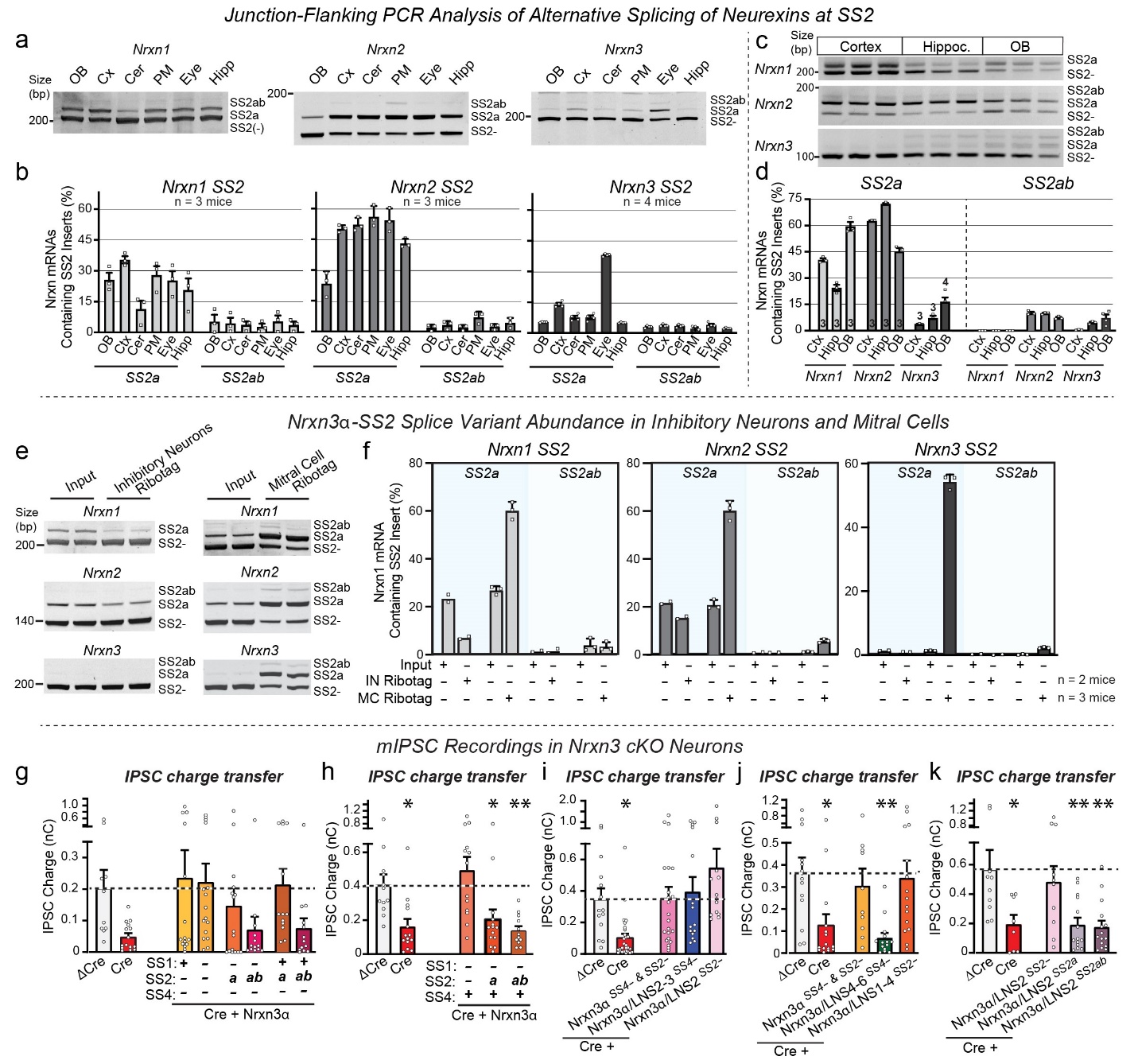


Figure S4: **Analyses of *Nrxn1-Nrxn3* mRNA Splicing at SS2 (a-f), and of synaptic charge transfer in Nrxn3 cKO rescue experiments (g-k)**

**a** & **b**. Analyses of the alternative splicing of *Nrxn1*-*Nrxn3* mRNAs in various brain regions at SS2, which is expressed in three variants: Two more common variants lacking (SS2-) or containing an 8 residue insert (SS2a), and a rare variant that includes the 8-residue ‘a’ insert and an additional 7-residue ‘b’ insert (SS2ab) (a, representative gels of junction-spanning RT-PCR products; b, summary graphs of the percentages of the SS2a and SS2ab mRNAs of the indicated neurexins).

**c** & **d**. Analyses of neurexin-SS2 alternative splicing in dissociated cultures from cortex (Ctx), hippocampus (Hipp), and OB confirms that most *Nrxn3* mRNAs lack an insert in SS2 (c, representative gels; d, summary graphs of the levels SS2 variants, SS2a and SS2ab, expressed as a % of total transcripts).

**e** & **f**. Junction-spanning RT-PCR measurements of SS2-, SS2a, and SS2ab splice variants in mRNAs isolated by RiboTag IPs from OB mitral/tufted cells and inhibitory neurons demonstrates that granule cells selectively express SS2- neurexin variants, whereas mitral/tufted cells abundantly express SS2+ variants (e, representative splice-junction PCR gels; f, summary graphs of the percentages of SS2a and SS2ab mRNA).

**g** - **k**. Nrxn3α-LNS2 constructs lacking an insert in SS2 rescue the suppression of evoked IPSC charge transfer induced by the deletion of *Nrxn3* in OB neurons. Experiments are the same as those shown in Figure 1l-1o and Figure 2b-2h.

Numerical data are means ± SEM; n’s (images/experiments) are indicated in the summary graph bars (a-f) and apply to all graphs in an experimental series or are described in the main figures. Statistical analyses were performed with a one-way analysis of variance (ANOVA) with Dunnett’s multiple comparison test (g-k), with * = p<0.05 and ** = p<0.01. Source data and statistical results are provided within the Source Data file. Scanned gels are provided in the Supplementary Information File.


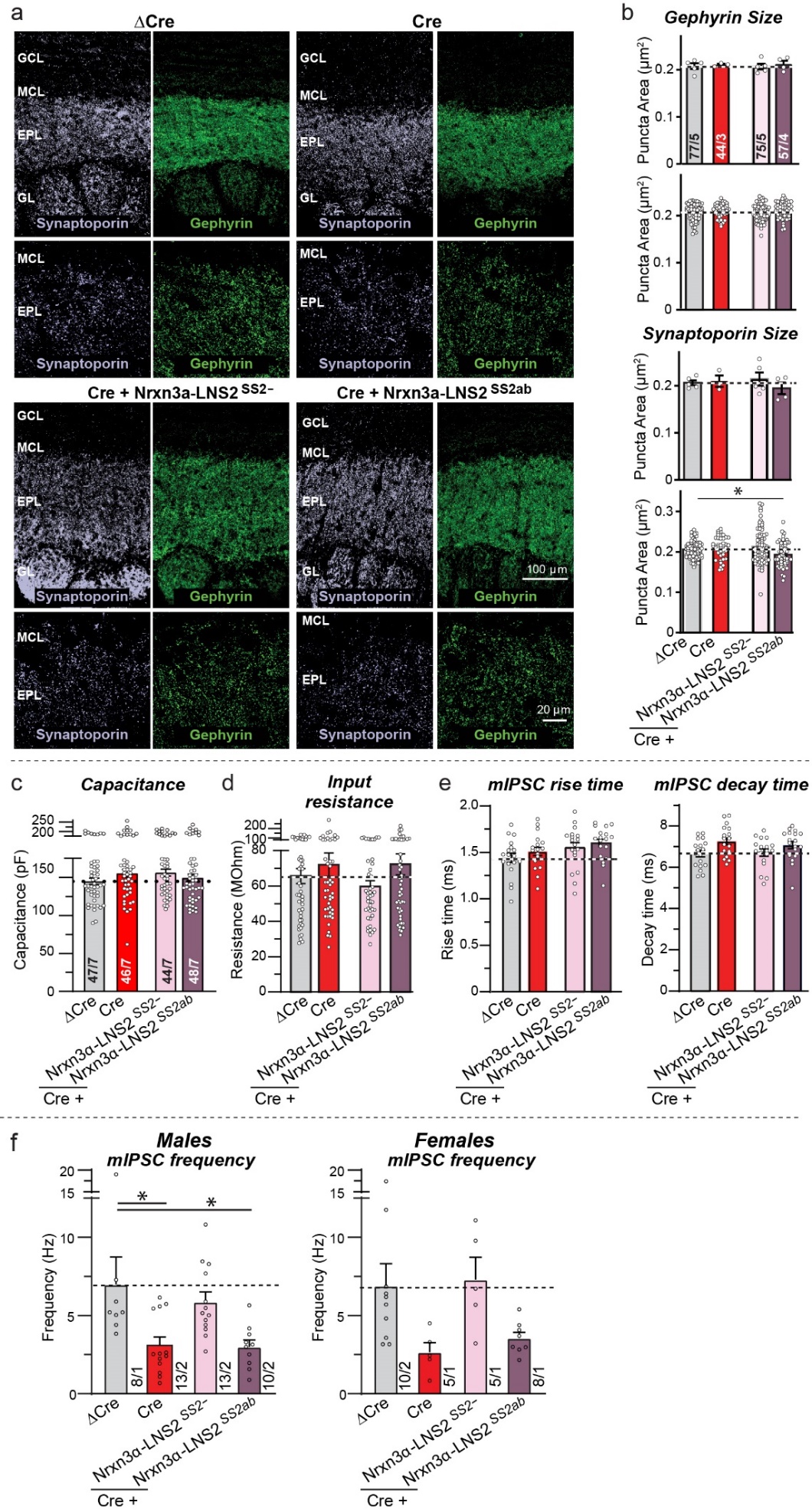


Figure S5: **Further data confirming that the *Nrxn3* deletion in the OB *in vivo* does not lower synapse numbers (a & b), but that the severe impairment in inhibitory GC🡪MC synapses induced by the *Nrxn3* deletion *in vivo* can be rescued with Nrxn3α^LNS2-^**

**a** & **b**. Conditional *Nrxn3* deletion and expression of Nrxn3α^LNS2-^ and Nrxn3α^LNS2ab^ rescue constructs in the OB *in vivo* do not alter the size of reciprocal mitral/granule cell synaptic puncta as analyzed by immunohistochemistry for a presynaptic (synaptoporin) and postsynaptic marker (gephyrin) (a, sample images; b, summary graphs of puncta size). Low magnification images (top) show that the overall anatomy of the OB circuit is intact. High magnification images imaged at Nyquist (bottom) were used for quantifying synaptic puncta. Note that puncta sizes in b are plotted both as per animal (top) and as per region-of-interest (bottom) because the latter approach is a standard of the field but artifactually boosts statistical significance independent of the actual number of experimental observations. Data complement those shown in Fig. 3.

**c** & **d**. Measurements of the cell capacitance (c) and input resistance (d) show that the conditional deletion of *Nrxn3* and the expression of Nrxn3α^LNS2-^ and Nrxn3α^LNS2ab^ rescue constructs does not significantly affect the basic electrical properties of neurons. Data complement those shown in Fig. 4.

**e**. Quantifications of the mIPSC rise and decay times uncover no changes induced by the conditional deletion of *Nrxn3* or the expression of Nrxn3α^LNS2-^ and Nrxn3α^LNS2ab^ rescue constructs in mitral cells in vivo. Data complement those shown in Fig. 4a-c.

**f**. Plots of the data shown in Figure 4a-4b separately for male and female mice to demonstrate that both genders exhibit similar phenotypes.

Numerical data are means ± SEM; n’s (images/experiments) are indicated in the summary graph bars and apply to all graphs in an experimental series. Statistical analyses were performed with a one-way analysis of variance (ANOVA) with Dunnett’s multiple comparison test comparing all samples to the ΔCre control condition (b) or Tukey’s multiple comparison test (c-f), with * = p<0.05. Source data and statistical results are provided within the Source Data file.


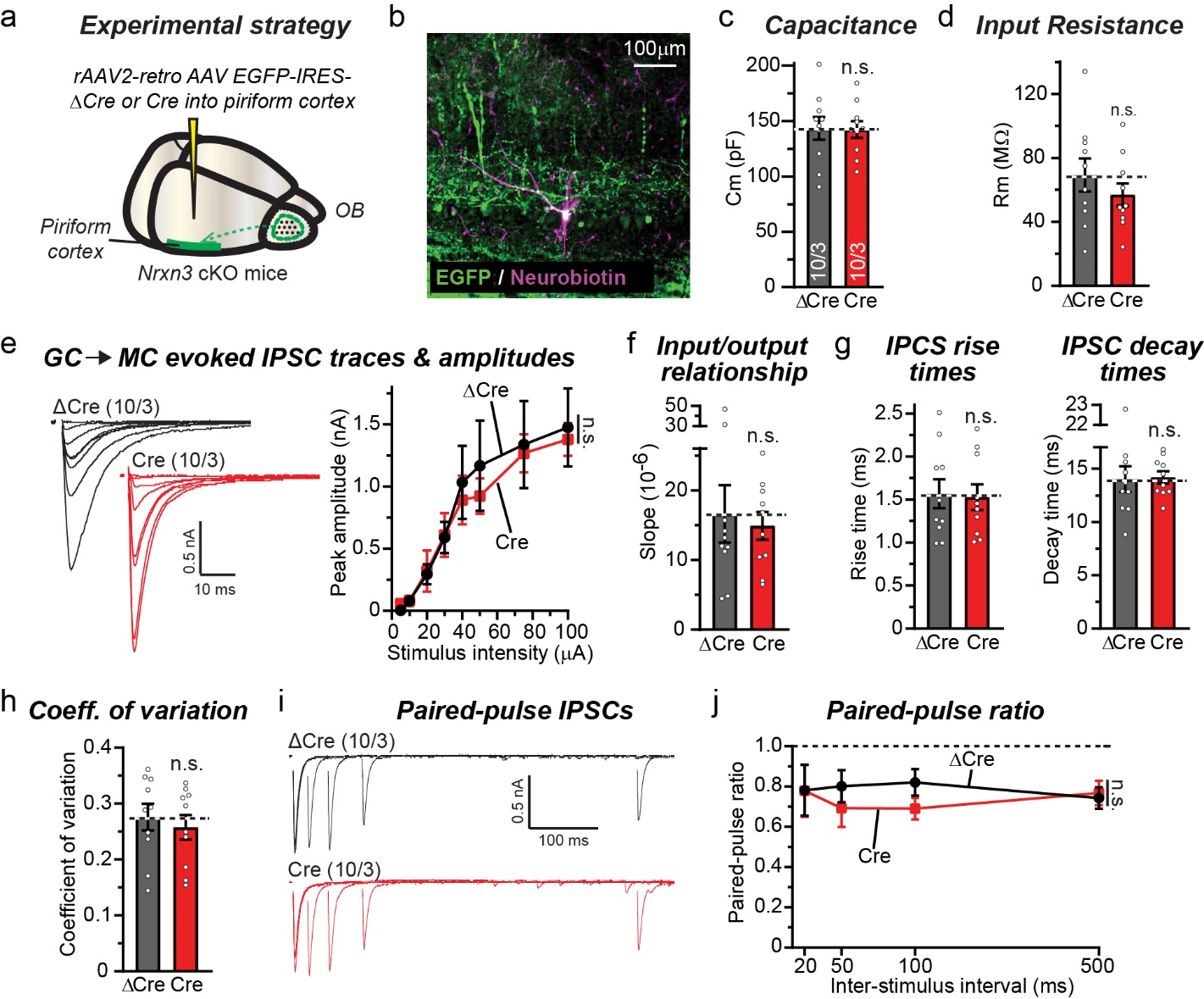


Figure S6: **Conditional deletion of *Nrxn3* in mitral cells of the OB *in vivo* via retro-AAV-Cre injected into the piriform cortex does not impair inhibitory GC🡪MC synapses**

**a**. Experimental design for mitral cell-specific *Nrxn3* deletions in the OB using retro-AAVs encoding ΔCre or Cre together with EGFP. retro-AAVs are injected into the piriform cortex of Nrxn3 *cKO* mice, where they infect axons projecting from OB mitral/tufted cells and thereby delete *Nrxn3* specifically in mitral/tufted cells of the OB.

**b**. Representative fluorescence image of an OB section from a mouse infected with AAVs expressing EGFP (red) in which a mitral cell was patched and filled with neurobiotin (purple).

**c** & **d**. *Nrxn3* deletion does not change the capacitance (c) or input resistance (d) of mitral cells.

**e-f**. The *Nrxn3* deletion does not alter the amplitude of IPSCs at GC🡪MC synapses evoked by extracellular stimulation, as documented by input/output curves to control for possible variations in stimulating electrode placement (e, left, representative IPSC traces; e, right, summary plot of input/output amplitude measurements; f, summary graph of the slope of the input/output curves).

**g**. The *Nrxn3* deletion in mitral cells does not give rise to a detectable change in the IPSC kinetics.

**h**. The *Nrxn3* deletion does not change the coefficient of variation of evoked IPSCs at GC🡪MC synapses, suggesting normal release probability.

**i** & **j**. Consistent with a normal release probability, the *Nrxn3* deletion does not change the paired-pulse ratio (i, representative traces; j, summary plot of the paired-pulse ratio).

Numerical data are means ± SEM; n’s (cells/experiments) are indicated in the summary graph bar (c) or above the sample traces (e and i) and apply to all graphs in an experimental series. Statistical analyses were performed using two-tailed unpaired *t* test in c, d, and f-h, and by two-way ANOVA with a Bonferroni multiple comparisons test e & j. Source data and statistical results are provided within the Source Data file.


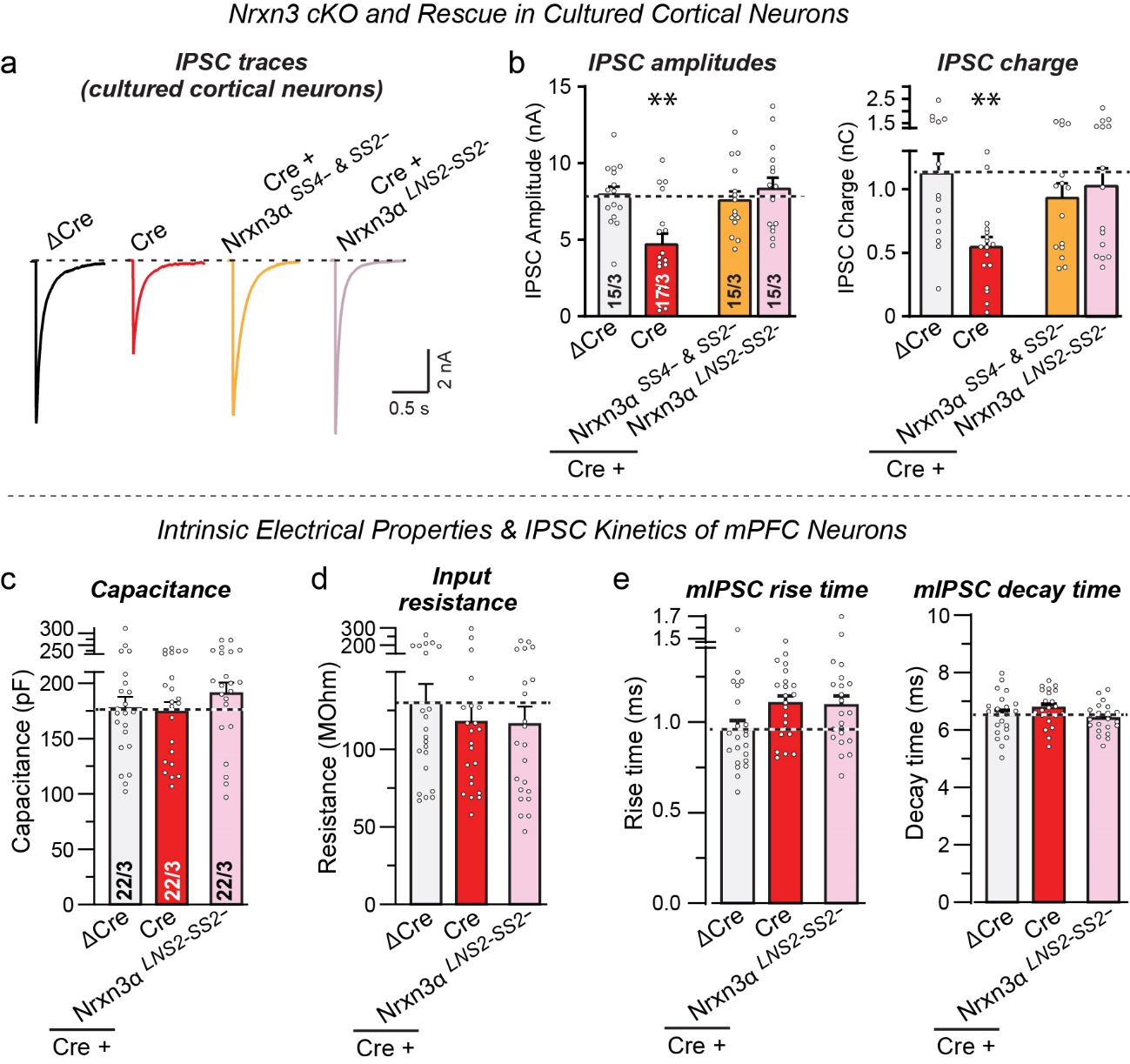


Figure S7: **Conditional *Nrxn3* deletion in cultured cortical neurons impairs evoked inhibitory synaptic transmission (a & b), and conditional *Nrxn3* deletion in the mPFC *in vivo* does not detectably alter the passive electrical properties of pyramidal neurons (c & d) or the kinetics of mIPSCs (e)**

**a** & **b**. Conditional deletion of *Nrxn3* in cultured cortical mouse neurons significantly decreases the amplitude and charge transfer of evoked IPSCs. Neurons were cultured from newborn *Nrxn3* cKO mice, infected with lentiviruses expressing ΔCre (control) or Cre at 4 days in culture, and analyzed at 14-16 days in culture (a, representative traces; b, summary graphs of the IPSC amplitude and charge transfer). The experiments provided the rationale for performing in vivo experiments shown in Fig. 5.

**c** & **d**. Measurements of the cell capacitance (c) and input resistance (d) show that the conditional deletion of *Nrxn3* and the expression of the Nrxn3α^LNS2-^ rescue construct in the mPFC in vivo does not significantly affect the basic electrical properties of neurons. Data complement those shown in Fig. 5.

**e**. Quantifications of the mIPSC rise and decay times uncover no changes induced by the conditional deletion of *Nrxn3* or the expression of the Nrxn3α^LNS2-^ rescue construct in the mPFC in vivo. Data complement those shown in Fig. 5.

Numerical data are means ± SEM; n’s (cells/experiments) are indicated in the summary graph bars and apply to all graphs in an experimental series. Statistical analyses were performed with a one-way analysis of variance (ANOVA) with Dunnett’s multiple comparison test comparing all samples to the ΔCre control condition (b) or with Tukey’s multiple comparison test (c-e), with * = p<0.05, ** = p<0.01, and *** = p<0.001. Source data and statistical results are provided within the Source Data file.


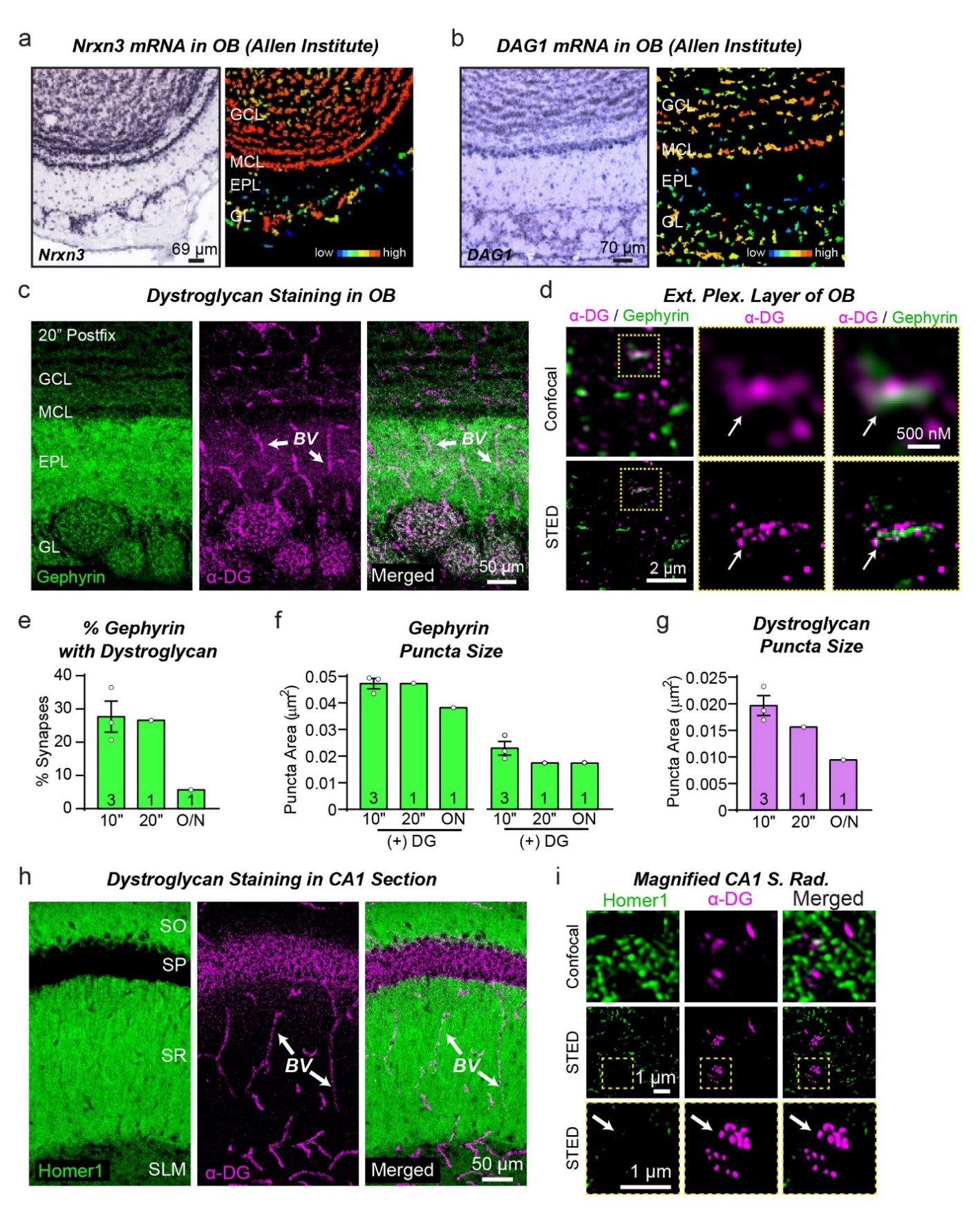


Figure S8: **Dystroglycan and *Nrxn3* are abundantly expressed in the mouse olfactory bulb**

**a-b.** *Nrxn3* and *Dag1* mRNA are widely distributed throughout the adult OB (left, original signal; right, expression heat map). Abbreviations: GCL, granule cell layer; MCL, mitral cell layer; EPL, external plexiform layer; GL, glomerular layer. In situ hybridization data are from the Allen Mouse Brain Atlas (Lein, E.S. et al. (2007); Nrxn3, https://mouse.brain-map.org/experiment/show/74000492; Dag1, https://mouse.brain-map.org/experiment/show/355892.

**c**. A brief 20 min post-fixation with 4% PFA permits robust detection of gephyrin (left) and dystroglycan puncta (middle) in synaptic layers of the OB. Arrows indicate dystroglycan associated with blood vessels (BV’s). Data complement those shown in Fig. 7.

**d.** STED super resolution imaging shows that gephyrin-containing inhibitory synapses frequently contain dystroglycan nanoclusters.

**e.** Approximately 30% of gephyrin-positive inhibitory synapses in the EPL contain at least one dystroglycan nanocluster. Quantification was performed on high magnification images obtained using STED microscopy. Considering that imaging was only performed in two dimensions, these numbers underestimate the total percentage of inhibitory synapses with dystroglycan. Data shown as averaged per animal.

**f**. The average size of inhibitory postsynaptic gephyrin discs is 2-fold larger when dystroglycan nanoclusters are present, regardless of the duration of post-fixation.

**g**. The size of dystroglycan clusters is larger under light fixation conditions.

**h-i**. Dystroglycan-positive synapses in hippocampal CA1 do not readily co-localize with the excitatory postsynaptic marker Homer1 (h, low magnification of CA1; i, top, high magnification confocal image; i, top, middle, high magnification STED image; i, bottom, single synapse magnification).

Numerical data are means ± SEM; n’s (animals) are indicated in the summary graphs and reflected in representative staining shown in Fig. 7a-c and S8c. Source data for all experiments are provided as a Source Data file.


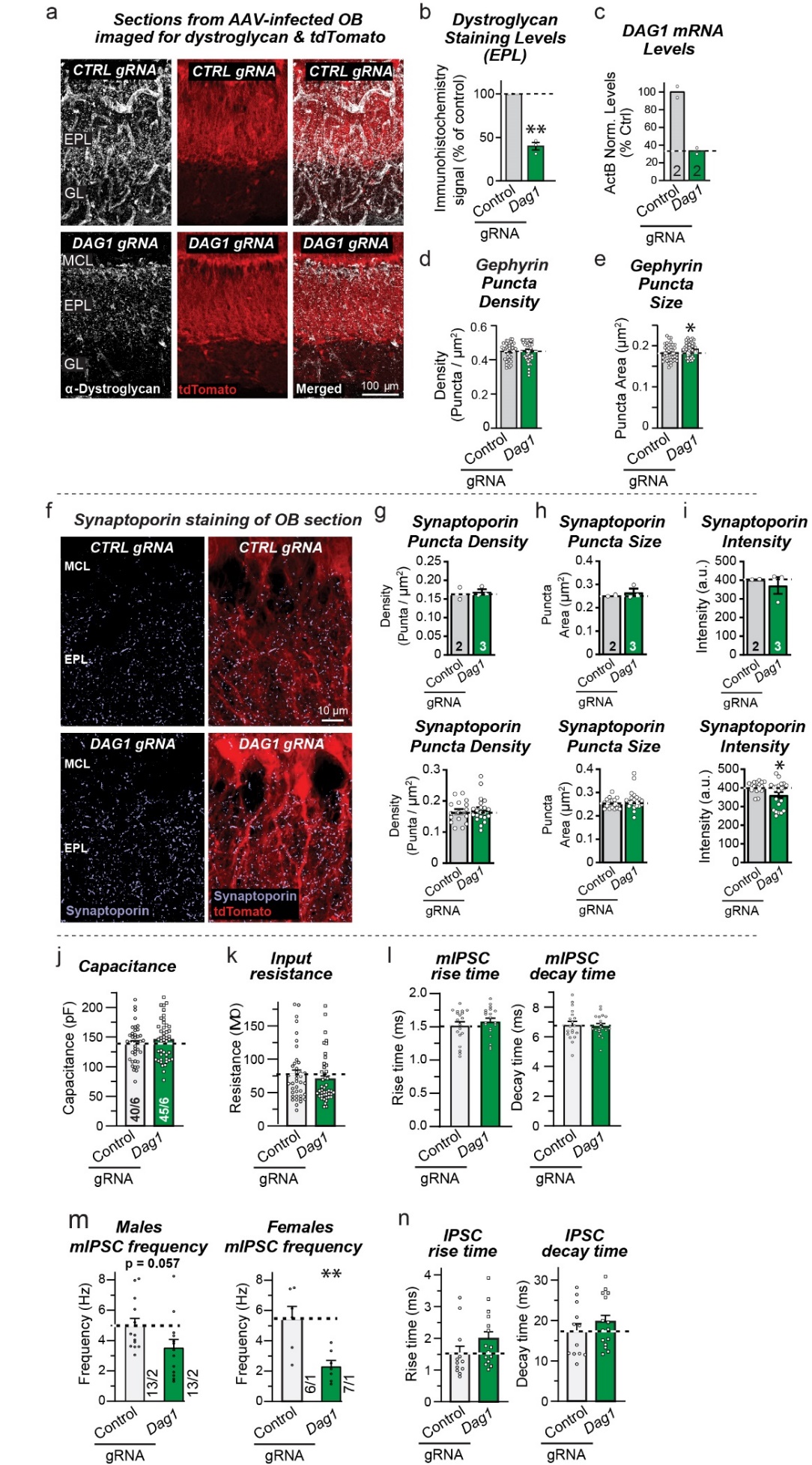


Figure S9: **Deletion of dystroglycan (*Dag1*) in the OB is efficient (a-c), doesn’t change inhibitory synapse numbers (d-i), and has no effect on the intrinsic electrical properties (j, k) and kinetics of mIPSCs (l) or evoked IPSCs (m) in mitral cells**

**a** & **b**. Analyses of the effect of the CRISPR-mediated deletion of dystroglycan (*Dag1*) in the OB by immunocytochemistry, revealing a ~60% loss of dystroglycan in the external plexiform layer (EPL) (a, representative images of OB sections stained for dystroglycan and tdTomato (to mark AAV-infected cells, see Fig. 8a-d); b, summary graph of the dystroglycan staining intensity)).

**c**. Deletion of dystroglycan (*Dag1*) in the OB suppresses dystroglycan mRNAs levels as measured with qRT-PCR.

**d & e**. The dystroglycan (*Dag1*) deletion has no effect on the density (d) or size (e) of gephyrin puncta. Summary graphs depict synaptic puncta quantified per region-of-interest, also referred to as pseudoreplicates. Experiment is the same as shown in Figure 8c & 8d.

**f-i**. The dystroglycan (*Dag1*) deletion has no effect on the density (g), size (h), or intensity (i) of synaptoporin puncta. Top, averaged per animal; bottom, pseudoreplicates.

**j** **& k**. Summary graphs of the capacitance (j) and input resistance (k) of mitral cells as a function of the dystroglycan deletion in the OB in vivo described in Figure 8f-8h demonstrate that the dystroglycan deletion does not detectably alter the size and membrane properties of the neurons.

**l**. Summary graphs of the mIPSC rise and decay times of mitral cells described in Figure 8f-8h.

**m**. Plots of the data shown in Figure 8f-8g separately for male and female mice to demonstrate that both sexes exhibit similar phenotypes.

**n**. Summary graphs of the IPSC rise and decay times monitored in mitral cells described in Figure 8i-8n.

Numerical data are means ± SEM; n’s (cells or images/experiments) are indicated in the summary graph bars and apply to all graphs in an experimental series. Statistical analyses were performed using two-tailed unpaired *t* test, with * = p<0.05 and ** = p<0.01. Source data and statistical results are provided within the Source Data file.


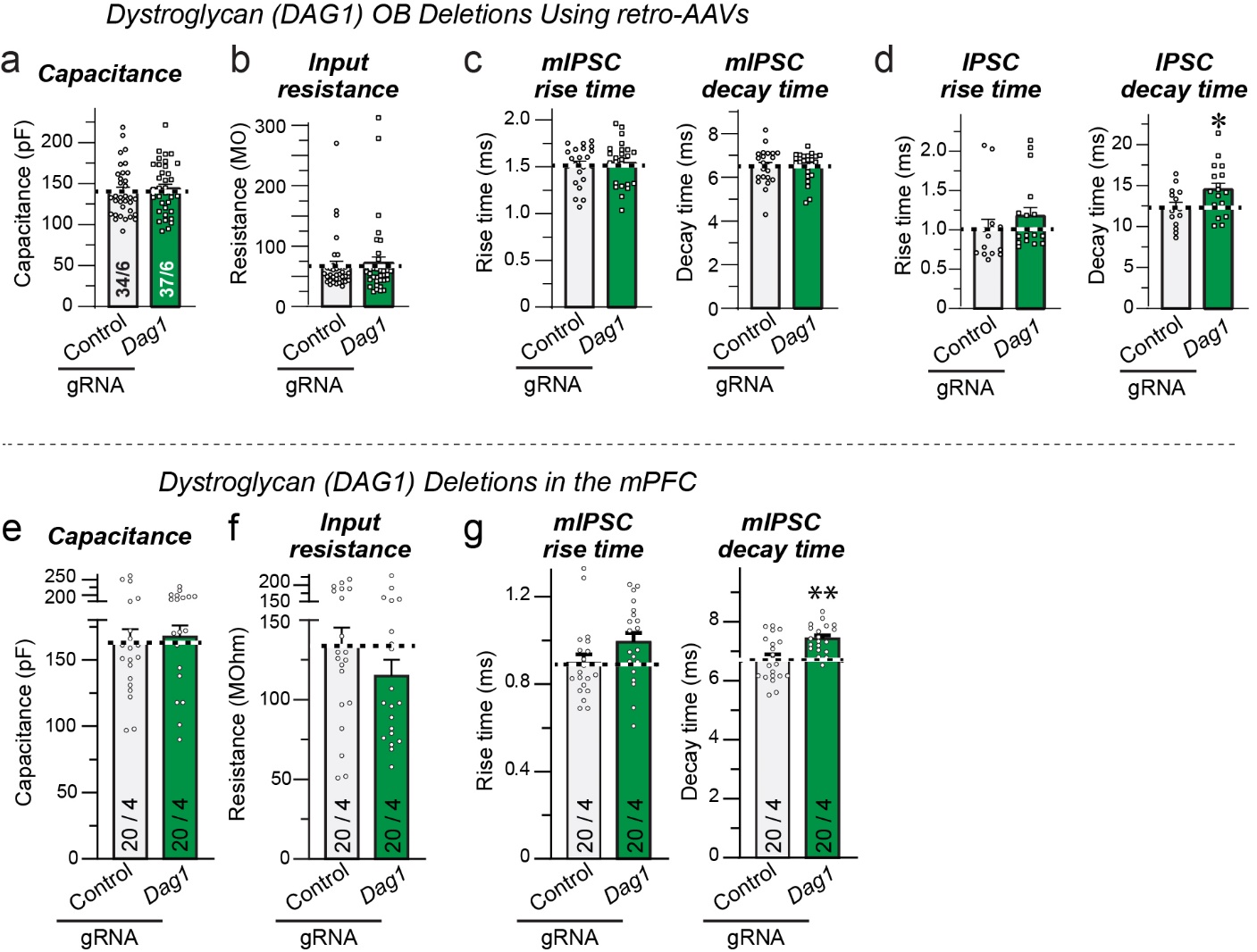


Figure S10: **Further characterization of the effect of CRISPR-mediated *in vivo* deletions of dystroglycan (*Dag1*) in mitral cells of the OB (a-e) and in the mPFC (f-h)**

**a** & **b**. Summary graphs of the capacitance (a) and input resistance (b) of mitral cells as a function of the dystroglycan deletion specifically only in mitral cells described in Figure 9a demonstrate that the dystroglycan deletion does not detectably alter cell size and membrane properties. Data correspond to the recordings shown in Figure 9f-9n.

**c**. Summary graphs of the mIPSC rise and decay times of mitral cells as a function of the dystroglycan deletion in the OB in vivo described in Figure 9f-9h demonstrate that the dystroglycan deletion does not induce a detectable change in the mIPSC kinetics.

**d**. Summary graphs of the IPSC rise and decay times of mitral cells as a function of the dystroglycan deletion in the OB in vivo described in Figure 9i-9n demonstrate that the dystroglycan deletion does not give rise to a major change in the IPSC kinetics.

**e** & **f**. Summary graphs of the capacitance (f) and input resistance (g) of pyramidal neurons in the mPFC as a function of the dystroglycan deletion in the mPFC in vivo described in Figure 9o-9r demonstrate that the dystroglycan deletion does not cause a major change in cell size and membrane properties.

**g**. Summary graphs of the mIPSC rise and decay times of layer 5 pyramidal neurons as a function of the dystroglycan deletion in the mPFC in vivo described in Figure 9p-9r demonstrate that the dystroglycan deletion does not cause a major change in the mIPSC kinetics.

Numerical data are means ± SEM; n’s (cells or images/experiments) are indicated in the summary graph bars and apply to all graphs in an experimental series. Statistical analyses were performed using a two-tailed unpaired *t* test, with * = p<0.05 and ** = p<0.01. Source data and statistical results are provided within the Source Data file.
