# Supplementary material for: A combinatorial code of neurexin-3 alternative splicing controls inhibitory synapses via a trans-synaptic dystroglycan signaling loop
Source: Nat Commun. 2023 Mar 30;14:1771. doi: 10.1038/s41467-023-36872-8 (PMC10063607; doi:10.1038/s41467-023-36872-8)
Supplement: Supplementary file 3 — Reporting Summary [file 41467_2023_36872_MOESM3_ESM.pdf]

nature portfolio

Corresponding author(s): Justin H. Trotter and Thomas C. Südhof

Last updated by author(s): 1/21/2023

Reporting Summary

Nature Portfolio wishes to improve the reproducibility of the work that we publish. This form provides structure for consistency and transparency in reporting. For further information on Nature Portfolio policies, see our Editorial Policies and the Editorial Policy Checklist.

Statistics

For all statistical analyses, confirm that the following items are present in the figure legend, table legend, main text, or Methods section.

n/a	Confirmed	
☐	☒	The exact sample size (n) for each experimental group/condition, given as a discrete number and unit of measurement
☐	☒	A statement on whether measurements were taken from distinct samples or whether the same sample was measured repeatedly
☐	☒	The statistical test(s) used AND whether they are one- or two-sided<br>*Only common tests should be described solely by name; describe more complex techniques in the Methods section.*
☐	☒	A description of all covariates tested
☐	☒	A description of any assumptions or corrections, such as tests of normality and adjustment for multiple comparisons
☐	☒	A full description of the statistical parameters including central tendency (e.g. means) or other basic estimates (e.g. regression coefficient) AND variation (e.g. standard deviation) or associated estimates of uncertainty (e.g. confidence intervals)
☐	☒	For null hypothesis testing, the test statistic (e.g. F, t, r) with confidence intervals, effect sizes, degrees of freedom and P value noted<br>*Give P values as exact values whenever suitable.*
☒	☐	For Bayesian analysis, information on the choice of priors and Markov chain Monte Carlo settings
☒	☐	For hierarchical and complex designs, identification of the appropriate level for tests and full reporting of outcomes
☒	☐	Estimates of effect sizes (e.g. Cohen's d, Pearson's r), indicating how they were calculated

Our web collection on statistics for biologists contains articles on many of the points above.

Software and code

Policy information about availability of computer code

Data collection	QuantStudio 3 (Thermo Fisher), NIS-Elements (Nikon), Image Lab 6.0.1 (Bio-Rad), Clampex 9 & 10.7 (Molecular Devises), STEDYCON, Huygen's Essentials
Data analysis	QuantStudio 3 (Thermo Fisher), Excel (Microsoft), NIS-Elements (Nikon), Image Lab 6.0.1 (Bio-Rad), Clampfit (Molecular Devises), GraphPad Prism 6 or 9

For manuscripts utilizing custom algorithms or software that are central to the research but not yet described in published literature, software must be made available to editors and reviewers. We strongly encourage code deposition in a community repository (e.g. GitHub). See the Nature Portfolio guidelines for submitting code & software for further information.

Data

Policy information about availability of data

All manuscripts must include a data availability statement. This statement should provide the following information, where applicable:

- Accession codes, unique identifiers, or web links for publicly available datasets
- A description of any restrictions on data availability
- For clinical datasets or third party data, please ensure that the statement adheres to our policy

Source data are provided within this paper. Additional data that support the findings of this study are available from the corresponding author upon reasonable request. Databases used for this study include https://gene.sfari.org/database/gene-scoring/ and the Allen Brain Atlas (i.e. Nrxn3, https://mouse.brain-map.org/experiment/show/74000492; Dag1, https://mouse.brain-map.org/experiment/show/355892).

Human research participants

Policy information about studies involving human research participants and Sex and Gender in Research.

Reporting on sex and gender	n/a
Population characteristics	n/a
Recruitment	n/a
Ethics oversight	n/a

Note that full information on the approval of the study protocol must also be provided in the manuscript.

Field-specific reporting

Please select the one below that is the best fit for your research. If you are not sure, read the appropriate sections before making your selection.

☒ Life sciences ☐ Behavioural & social sciences ☐ Ecological, evolutionary & environmental sciences

For a reference copy of the document with all sections, see nature.com/documents/nr-reporting-summary-flat.pdf

Life sciences study design

All studies must disclose on these points even when the disclosure is negative.

Sample size	Sample sizes are indicated in the figure legends and/or figures, and were determined based on historical practices in the lab (Chen et al., Neuron, 2017; Luo et al., EMBO J, 2020) to claim statistical effects. Careful effort was made when possible to average per true biological replicate rather than use pseudo-replicates. We did not conduct a power analysis or employ other statistical methods to predetermine sample size.
Data exclusions	No data were excluded from the analyses.
Replication	Careful effort was made to incorporate variation by sampling from different litters and wherever possible, balance sampling of sexes for analysis. As detailed in the materials and methods, for all culture experiments, a typical independent culture batch included pooled tissue from ~6 pups regardless of sex. Key phenotypes were reproduced across many different molecular manipulations (rescue and CRISPR) and measurement types (e.g. mIPSC, evoked IPSC, PPR) both in vitro and in vivo. Attempts at replication were successful. All experiments have underwent at least 3 independent replications or included at least 3 biological replicates, as is defined in the figure legends and statistics and reproducibility section.
Randomization	Allocation was random.
Blinding	For all staining and electrophysiology experiments, data was collected blind to genotype or treatment. The only experiments that were not blinded were splice junction PCR experiments in Fig. S3-S4, since analysis can be performed readily without risk of experimental bias.

Reporting for specific materials, systems and methods

We require information from authors about some types of materials, experimental systems and methods used in many studies. Here, indicate whether each material, system or method listed is relevant to your study. If you are not sure if a list item applies to your research, read the appropriate section before selecting a response.

Materials & experimental systems

n/a	Involved in the study
☐	☒ Antibodies
☐	☒ Eukaryotic cell lines
☒	☐ Palaeontology and archaeology
☐	☒ Animals and other organisms
☒	☐ Clinical data
☒	☐ Dual use research of concern

Methods

n/a	Involved in the study
☒	☐ ChIP-seq
☒	☐ Flow cytometry
☒	☐ MRI-based neuroimaging

Antibodies

Antibodies used	The following antibodies were used at the indicated concentrations (IHC-immunohistochemistry; ICC-immunocytochemistry):<br>1. anti-alpha-Dystroglycan rabbit [45-3] (Abcam Cat# ab199768; 1:250 IHC)<br>2. anti-HA rabbit (Cell Signaling Cat# 3724; 1:500 IHC)<br>3, anti-Gephyrin mouse (Synaptic Systems Cat# 147 011; 1:1000 ICC)<br>4. anti-Gephyrin guinea pig (Synaptic Systems Cat# 147 318 ; 1:250-1:500 IHC)<br>5. anti-Homer1 rabbit (Synaptic Systems Cat# 160 003, 1:500-1:1000)<br>6. anti-MAP2 chicken (Encorbio Cat# CPCA-MAP2; 1:1000)<br>7. anti-GABAARα1 (Synaptic Systems Cat# 224 203; 1:250 live ICC)<br>8. anti-GABAARα2 (Synaptic Systems Cat# 224 103; 1:250 live ICC)<br>9. anti-GABAARγ2 (Synaptic Systems Cat# 224 003; 1:250 live ICC)<br>10. anti-Synaptophysin-2 rabbit (homemade, Wang et al., 2021; 1:500)<br>11. anti-vGAT guinea pig (Synaptic Systems Cat# 131 004; 1:1000 ICC)<br>12. anti-Dystroglycan, clone IIH6C4 mouse (Millipore, Cat# 05-593; 1:250 IHC)<br>13. Goat anti-Mouse IgM Heavy Chain Alexa594 (ThermoFisher, A-21044; 1:400, IHC/STED)<br>14. Goat anti-Mouse IgG Alexa546 (ThermoFisher, A11003; 1:1000, ICC/IHC)<br>15. Goat anti-Mouse IgG Alexa405 (ThermoFisher, A31553; 1:1000, ICC/IHC)<br>16. Goat anti-Rabbit IgG Alexa405 (ThermoFisher, A31556; 1:1000, ICC/IHC)<br>17. Goat anti-Rabbit IgG Alexa546 (ThermoFisher, A11010; 1:1000, ICC/IHC)<br>18. Goat anti-Rabbit IgG STAR Red (Abberior, STRED-1001; 1:400, IHC/STED)<br>19. Goat anti-Rabbit IgG STAR460L (Abberior, ST460L-1002; 1:400, IHC/STED)<br>20. Goat anti-Rabbit IgG CF568 (Biotium, 20098-1 mg; 1:3000, IHC)<br>21. Goat anti-Guinea Pig IgG Alexa647 (ThermoFisher, A21450; 1:1000-1:3000, ICC/IHC)<br>22. Goat anti-Guinea Pig IgG STAR Red (Abberior, STRED-1006; 1:400, IHC/STED)<br>23. Goat anti-Chicken Igy Alexa488 (ThermoFisher, A11039; 1:1000, ICC)
Validation	The following antibodies were used at the indicated concentrations (IHC-immunohistochemistry; ICC-immunocytochemistry):<br>1. anti-alpha-Dystroglycan rabbit [45-3] (Abcam Cat# ab199768), validated here; vendor provides validation with staining and immunoblotting; also generated/validated by Furtunato et al., 2014<br>2. anti-HA rabbit (Cell Signaling Cat# 3724), Trotter et al., 2019. JCB 219(8):2677-2698 (IHC)<br>3, anti-Gephyrin mouse (Synaptic Systems Cat# 147 011), validated in knockout by vendor (ICC)<br>4. anti-Gephyrin guinea pig (Synaptic Systems Cat# 147 318), validated in knockout by vendor (ICC)<br>5. anti-Homer1 rabbit (Synaptic Systems Cat# 160 003), validated by vendorfor ICC/IHC and other applications (ICC)<br>6. anti-MAP2 chicken (Encorbio Cat# CPCA-MAP2), validated by vendor for ICC/IHC and other applications (ICC)<br>7. anti-GABAARα1 (Synaptic Systems Cat# 224 203), validated by vendor for ICC/IHC/ and other applications (ICC)<br>8. anti-GABAARα2 (Synaptic Systems Cat# 224 103), validated by vendor in KO (ICC)<br>9. anti-GABAARγ2 (Synaptic Systems Cat# 224 003), validated by vendor in KO (ICC)<br>10. anti-Synaptophysin-2 rabbit (homemade), Wang et al., 2021<br>11. anti-vGAT guinea pig (Synaptic Systems Cat# 131 004), validated by vendor in KO (ICC)<br>12. anti-Dystroglycan, clone IIH6C4 mouse (Millipore, Cat# 05-593), vendor (ICC) and several papers referenced in the manuscript (i.e. Miller and Wright, 2021; Uezu et al., 2019; Früh et al., 2016)<br>13-23. All secondaries extensively validated by vendors, previous papers from lab, and often test with omission of primary antibodies during initial optimization of staining conditions.

Eukaryotic cell lines

Policy information about cell lines and Sex and Gender in Research

Cell line source(s)	HEK293T/17 cells (ATCC, CRL-11268) were from stock original obtained in 2018.
Authentication	The cell line was not authenticated (other than by morphology and passage).
Mycoplasma contamination	Cell lines were tested negative for mycoplasma contamination using the fluorochrome Hoechst DNA stain and the direct culture method.

Commonly misidentified lines (See ICLAC register)	No commonly misidentified lines were used.

Animals and other research organisms

Policy information about studies involving animals; ARRIVE guidelines recommended for reporting animal research, and Sex and Gender in Research

Laboratory animals	Experimental mice included both males and females between the ages of 0-60 days. The following strains were used:<br>Nrxn3 conditional knockout (cKO) (Aoto et al., 2015)<br>Constitutive Cas9 KI mice (Jax, stock# 024858)<br>Conditional Cas9 KI mice (Jax, stock# 024857)<br>Tbet-Cre (Jax, stock# 024507)<br>vGAT-Cre (Jax, stock# 028862)<br>RiboTag mice (Jax, stock# 029977)<br>Wild-type CD1 mice (Jax)<br><br>All mice were weaned at 20 days of age and housed in groups of 2 to 5 on a 12 hr light/dark cycle with access to food and water ad libidum and 40-60% humidity. Room temperatures were maintained at approximately 22C. All procedures conformed to National Institutes of Health Guidelines for the Care and Use of Laboratory Mice and were approved by the Stanford Animal Use Committees [Administrative Panel for Laboratory Animal Care (APLAC/) Institutional Animal Care and Use Committee (IACUC)].
Wild animals	No wild animals were involved in this study.
Reporting on sex	As defined in the materials and methods, for all cell culture experiments, multiple early postnatal pups were pooled regardless of sex. Typically, a given independent culture included tissue from 6 pups and each independent experiment should contain approximately equal numbers of males and females. Because in vitro phenotypes were reproducible across many manipulations we did not anticipate that the in vivo functions would be sex-dependent. Thus, for most in vivo experiments, we did not consider sex but tried to sample from males and females as much as possible. Nevertheless, we have disaggregated the data for two key experiments (Fig. S5f and S9m) and confirmed that our major in vivo phenotypes exist in both males and females as either a trend or with statistical significance. We have also noted sex for relevant in vivo experiments in the Source Data file.
Field-collected samples	This study did not involve field-collected samples.
Ethics oversight	All procedures conformed to National Institutes of Health Guidelines for the Care and Use of Laboratory Mice and were approved by the Stanford Animal Use Committees [Administrative Panel for Laboratory Animal Care (APLAC/) Institutional Animal Care and Use Committee (IACUC)].

Note that full information on the approval of the study protocol must also be provided in the manuscript.
