# Supplementary material for: A combinatorial code of neurexin-3 alternative splicing controls inhibitory synapses via a trans-synaptic dystroglycan signaling loop
Source: Nat Commun. 2023 Mar 30;14:1771. doi: 10.1038/s41467-023-36872-8 (PMC10063607; doi:10.1038/s41467-023-36872-8)
Supplement: Supplementary file 4 — Source Data [file 41467_2023_36872_MOESM4_ESM.pdf]

IPSC Amplitude (in nA)

[illegible]	[illegible]	[illegible]	[illegible]	[illegible]	[illegible]	[illegible]	[illegible]	[illegible]
	1.308107	2.713652	2.358691	2.53538	3.437096	0.1358272	3.83426	0.1361828
	4.525092	0.5609894	2.536092	9.171696	0.614819	0.6948296	5.820393	0.1362654
	3.466079	4.846139	5.141919	4.763264	2.953832	1.094869	1.877572	0.6228734
	3.823068	1.415487	5.765168	2.364167	5.747629	0.5313798	0.4144319	
	8.219751	1.883643	1.918545	2.187961	0.3060608	9.553122	3.488609	
			6.169101					
	0.9096411	0.0375881		5.876596	0.1777632	1.08261	1.26387	0.03424968
	0.2965365	0.1621298	3.55373	0.1025707	3.022207	1.117108	2.891118	0.07234917
	0.964915	0.2572851	0.4449716	5.926176	0.215094	2.121248	2.352317	0.01845643
	1.932971	0.01661155	1.166468	2.024837	0.367549	0.2574619	1.067538	0.1704715
	0.1207189	0.1314204	1.366085		0.3393845			
						5.243177	3.858481	0.1293892
	1.341731	0.3422128		2.911407	1.329099	4.302327	8.780096	0.1048829
	9.187503	0.1464321	2.874695	5.535801	6.98343	7.977143	4.077941	0.04133365
	8.92882	0.6201663	9.682705	1.393036	0.3492326	2.010459	9.358041	0.02142173
	0.8409352	1.893116	4.362415	4.587543	2.076822	3.546144		0.2541922
	3.989505	2.021023	5.728086	5.584713	0.05244542			
		0.9643376	2.717944					
	3.269317							
	3.955259							
	4.82109							
	5.171583							
	1.646953							
	4.991968							
	3.133881							
	3.012914							
	0.7626118							
	1.31847							
	2.196644							
	7.453976							
	11.76682							
	0.693166							
	5.418999							
[illegible]	[illegible]	[illegible]	[illegible]	[illegible]	[illegible]	[illegible]	[illegible]	[illegible]
[illegible]	[illegible]	[illegible]	[illegible]	[illegible]	[illegible]	[illegible]	[illegible]	[illegible]
[illegible]	[illegible]							
[illegible]		[illegible]	[illegible]	[illegible]	[illegible]	[illegible]	[illegible]	[illegible]

IPSC Amplitude

[illegible]	[illegible]	[illegible]
	2.86929126	1.793195068
	6.410631836	0.168608292
	3.472938477	0.227737854
	6.378326172	1.263599121
	2.872906738	0.692398254
	0.41430423	0.151052032
	2.848843262	4.59476709
	7.015697266	2.255911621
	10.01822949	0.242272629
	9.523632813	1.005764587
	7.295168992	7.203474121
	8.153916992	4.781345703
	2.528153564	3.364261963
	4.531416016	0.244389206
	0.238221909	3.163719727
		0.635532898
	9.616870117	
	2.895237061	4.210188965
	1.71219873	4.83412207
	3.449808838	1.776162842
		4.12428125
	9.342532227	0.610080486
	1.922970093	0.655162964
	13.50410547	4.668575684
		9.272292969
[illegible]	[illegible]	[illegible]
[illegible]	[illegible]	[illegible]
[illegible]	[illegible]	
[illegible]	[illegible]	
[illegible]	[illegible]	
[illegible]	[illegible]	

NMDA EPSC Amplitude

[illegible]	[illegible]	[illegible]	[illegible]	[illegible]	[illegible]	[illegible]	[illegible]	[illegible]
	0.843480042	0.510406067	0.504426147	0.85314856	0.231050995	0.1779715	0.6340576	0.3587506
	0.488039764	0.239658493	0.559984802	0.229114563	0.582827576	0.274544	0.1480792	1.113251
	0.155575974	0.38148842	0.450352722	0.649893921	1.218014038	0.58108	0.4899805	0.1530507
	0.25195488	0.221069696	0.514592224	0.837359863	0.878408325	0.1247752	0.1637157	0.1970083
			0.274330872	0.377978882	0.776037842			
	0.443863464	0.891197205				0.8431519	0.6854857	0.5414064
	0.930544617	1.044407104	0.454980145	0.483229645	0.291473175	0.6272863	0.6492874	0.5838781
	0.6362948	0.500726074	0.327141418	0.613577332	0.625271118	0.234286	0.3018	0.5820643
	0.359873596	0.405643219	0.623242432	0.132191742	0.75913855	1.208824	0.4829763	0.3577665
	0.424861786	0.593328369	0.451396606	0.520339478	0.299733093	0.4221451	0.431575	0.7540398
			0.516902405	0.247093155	0.29471991			
	0.436822266	1.69291394				0.5111649	0.8469136	1.078833
	0.436312347	0.99753418	0.671119995	1.095247559	0.686210022	1.567932	0.7899121	0.5293716
	0.451890045	0.663772583	0.710448303	0.871100037	1.60243811	0.3222314	0.3624017	0.8599769
	0.852368164	0.786927795	0.280302704	0.802574524	0.58892218	0.4284198	0.2712822	0.8198214
	0.9806851	0.665802002	0.418979584	1.527552612			0.5743439	0.5411661
	0.73125177							
	0.433539185							
	0.462208069							
	0.76885199							
	0.272403839							
	0.499698395							
	0.282589203							
	0.88730658							
	0.802724731							
	0.883231323							
	1.400634033							
	0.211800415							
	1.233322632							
	0.883962708							
[illegible]	[illegible]	[illegible]	[illegible]	[illegible]	[illegible]	[illegible]	[illegible]	[illegible]
[illegible]	[illegible]	[illegible]	[illegible]	[illegible]	[illegible]	[illegible]	[illegible]	[illegible]
[illegible]	[illegible]							
[illegible]								

NMDA EPSC Amplitude

[illegible]	[illegible]	[illegible]
	0.838889282	0.296781555
	0.52664447	0.711409119
	2.326744873	3.310697754
	0.765518982	0.566563477
	0.392482422	0.603948059
	0.484812897	0.195353943
	0.188860138	0.740680522
	0.491620819	1.304493774
	0.85169519	0.327481049
	1.326748657	0.659894165
	0.356746277	
	0.948073486	0.40203009
	1.212956543	1.225114868
	0.466608429	0.331909454
	0.304508942	0.912628174
	0.720377991	0.878454834
[illegible]	[illegible]	[illegible]
[illegible]	[illegible]	[illegible]
[illegible]	[illegible]	
[illegible]	[illegible]	
[illegible]	[illegible]	
[illegible]		

[illegible]	Homer- Inh. Synapse Density, Avg. Per Culture	
	ΔCre	Cre
	0.025856938	0.026958951
	0.034562879	0.025131553
	0.035464146	0.059334326
[illegible]	[illegible]	[illegible]
[illegible]	[illegible]	[illegible]
[illegible]	[illegible]	
[illegible]	[illegible]	
[illegible]	[illegible]	
[illegible]	[illegible]	

	Homer+ Inh. Synapse Density, Avg. Per Culture	
	ΔCre	Cre
	0.031457167	0.031082589
	0.051703792	0.059505883
	0.045683349	0.071423718
[illegible]	[illegible]	[illegible]
[illegible]	[illegible]	[illegible]
[illegible]	[illegible]	
[illegible]	[illegible]	
[illegible]	[illegible]	
[illegible]	[illegible]	

	Total Inh. Synapses, Avg. Culture	
	ΔCre	Cre
	0.057324105	0.05802152
	0.086268672	0.084637436
	0.081147495	0.130758044
[illegible]	[illegible]	[illegible]
[illegible]	[illegible]	[illegible]
[illegible]	[illegible]	
[illegible]	[illegible]	
[illegible]	[illegible]	
[illegible]	[illegible]	

Homer- Inh. Synapse Density, Pseudoreplicate		Homer+ Inh. Synapse Density, Pseudoreplicate		Total Inh. Synapses, Pseudoreplicate	
ΔCre	Cre	ΔCre	Cre	ΔCre	Cre
0	0.114263	0	0.025621	0	0.139884
0.036751194	0	0.11452803	0.024564	0.151279224	0.024564
[illegible]	[illegible]	[illegible]	[illegible]	[illegible]	[illegible]
[illegible]	[illegible]	[illegible]	[illegible]	[illegible]	[illegible]
0	0.021084	0.050057819	0	0.050057819	0.021084
0.057327427	0.013644	0	0.016518	0.057327427	0.030162
0.022691173	0.086374	0	0.018285	0.022691173	0.104659
[illegible]	[illegible]	[illegible]	[illegible]	[illegible]	[illegible]
[illegible]	[illegible]	[illegible]	[illegible]	[illegible]	[illegible]
0.036251586	0.017461	0.018125793	0.017461	0.054377379	0.034922
		0.063286	0	0.136789	0
0.073504	0				
		[illegible]	[illegible]	[illegible]	[illegible]
[illegible]	[illegible]				
		[illegible]	[illegible]	[illegible]	[illegible]
[illegible]	[illegible]				
		0.064246	0.095715	0.064246	0.128719
0	0.033003				
		0.066638	0.06404	0.087393	0.06404
0.020756	0				
		0	0.052482	0.04386	0.052482
0.04386	0				
		0	0.023004	0.016412	0.023004
0.016412	0				
		0.022676	0.043234	0.022676	0.108085
0	0.064851				
			0.033636		0.033636
	0				
			0		0
	0				
0	0.067736	0	0.088149	0	0.155885
0.021110407	0	0.042220815	0.106201	0.063331222	0.106201
0	0.0163	0	0.017434	0	0.033734
0	0.034351	0.038647343	0.062073	0.038647343	0.096424
0.025980774	0	0	0.060334	0.025980774	0.060334
0.035495156	0.060584	0	0.177157	0.035495156	0.23774
0.024491795	0	0.125077934	0.06288	0.14956973	0.06288
0.082781457	0.015284	0.027593819	0.015284	0.110375276	0.030567
0.02392917	0.035199	0	0.123196	0.02392917	0.158395
				0.05027473	0
0	0	0.05027473	0		
				0	0.04940646
0	0	0	0.04940646		
				0.110762375	0.027446137
0.023852117	0	0.086910258	0.027446137		
				0.264536223	0.1595716
0.158101203	0.064293524	0.10643502	0.095278075		
				0.151385526	0.032346189
0.075692763	0.01795977	0.075692763	0.014386419		
				0	0.074607711
0	0.032784206	0	0.041823505		
				0.295032466	0.17334556
0.06844222	0.083109142	0.226590246	0.090236419		
				0.110649943	0
0.036883314	0	0.073766628	0		
0.015263	0.044713	0.015263	0.022356	0.030525	0.067069
0.023132	0.08098	0.030807	0.110908	0.053939	0.191888
0.01996	0.123101	0.165937	0.084691	0.185897	0.207792
0	0.127988	0	0.120634	0	0.248622
0.05062	0.131962	0.07593	0.099481	0.12655	0.231442
0.073452	0.01908	0.037002	0	0.110454	0.01908
0.044588	0.134143	0.022207	0.045362	0.066795	0.179505
0.024655	0.057871	0.021575	0.018028	0.04623	0.075898
0.027122		0.12874		0.155863	0.071383
					0.197546
0.099865	0.025176	0.044593	0.046207	0.144458	
					0.019712
0.09473	0.059673	0	0.137874	0.09473	
					0.116083
0	0.019712	0	0	0	
					0.155217
0.039805	0.015642	0.061563	0.100441	0.101369	
					0.158217
0.034973	0.04729	0	0.107726	0.034973	
					0.117128
0.033858	0.016801	0.047887	0.141416	0.081745	
					0.035746
0.01638	0.027345	0.105151	0.089783	0.121531	
0	0.017873	0.029551	0.017873	0.029551	

[illegible]	Homer- Pseudoreplicate		[illegible]	Homer+ Pseudoreplicate		[illegible]	Total Pseudoreplicate	
[illegible]	[illegible]	[illegible]	[illegible]	[illegible]	[illegible]	[illegible]	[illegible]	[illegible]
[illegible]	[illegible]	[illegible]	[illegible]	[illegible]	[illegible]	[illegible]	[illegible]	[illegible]
[illegible]	[illegible]		[illegible]	[illegible]		[illegible]	[illegible]	
[illegible]	[illegible]		[illegible]	[illegible]		[illegible]	[illegible]	
[illegible]	[illegible]		[illegible]	[illegible]		[illegible]	[illegible]	
[illegible]	[illegible]		[illegible]	[illegible]		[illegible]	[illegible]	

[illegible]	[illegible]	[illegible]	[illegible]	[illegible]	[illegible]	[illegible]	[illegible]	[illegible]
	7.158001953	0.05638467	0.976806641	7.513725586	0.013424594	0.27601474	2.48929	0.03098145
	3.564458496	0.959856567	9.303960938	0.807631226	0.224725555	0.057293545	1.582867	0.0286377
	0.748796753	0.327402435	6.545655273	7.216987305	0.061624313	0.048894482	5.970719	0.2374261
	4.344000977	0.159571869	0.026782821		1.448972168	0.071039917	1.879897	1.665018
		0.317850311	0.080865005	1.460355347	2.972604736	0.324068512	4.465053	1.060264
	1.999592773			0.733286377				0.1085086
	10.84047559	0.698164063	0.6446297	3.452107666	3.275814453	1.451843628	0.5565224	
		2.233148926	1.054497192	0.025101412	8.536992188	0.255865677	0.554101	7.741561
	2.118257813	0.40677478	0.941948181	0.153724091	3.938776611	1.071834473	4.752984	0.5058947
	2.24265625	0.585594055	0.361753601		8.761864258	0.342019287	6.888483	0.327248
	1.825074951	0.240332901	0.369937012	2.735105957		1.680742188	6.105039	0.09728783
	3.605583008			1.193963013	3.630581299			
		1.695481445	2.042491943	0.454282684	1.619797363	5.985065918	1.083612	1.023029
		1.000462158	0.6124375	2.632481445	2.323583984	0.16327327	4.832414	1.091364
		1.635197876	4.98025293	1.937458008		0.743617798	1.776256	0.2653829
		1.324406128	7.16244873			1.984326294		1.784644
		1.635947144	2.755767334	3.820648193				
				2.347461914				
				5.581985352				
				6.976185059				
[illegible]	[illegible]	[illegible]	[illegible]	[illegible]	[illegible]	[illegible]	[illegible]	[illegible]
[illegible]	[illegible]	[illegible]	[illegible]	[illegible]	[illegible]	[illegible]	[illegible]	[illegible]
[illegible]	[illegible]							
[illegible]		[illegible]	[illegible]	[illegible]	[illegible]	[illegible]	[illegible]	[illegible]

[illegible]	[illegible]	[illegible]	[illegible]	[illegible]	[illegible]
	4.128657227	2.647081523	2.679908203	10.30027051	0.959768005
	8.755003906	1.28718042	8.100171387	2.073922363	4.841379883
	6.621474609	1.773821045	3.505862734	0.543865662	4.843619629
	4.467369629	7.199003906		0.416026168	5.122117188
	5.713401855	6.743768802	10.65703613		
			5.645251953	2.87163623	2.531376953
	3.064416504	1.568126587	8.888024414	3.213451172	2.778970947
	12.06360547	3.781130615	8.730179688	2.865221924	2.181524414
	8.039263184	8.861204102	8.288791016	4.73110498	1.766531372
	5.970728027	3.097463623		5.685089844	4.041144287
	9.4551875	5.234327148	5.559492188		
			5.803757324	2.9341604	0.55430719
	6.584349609	0.8770495	7.778402832	2.583173584	2.17753833
	6.807844727	2.895778809	10.36684766	4.547365723	2.519941162
		0.451543579	6.310141602	0.990324341	0.976242188
		0.339794373		1.805053711	
[illegible]	[illegible]	[illegible]	[illegible]	[illegible]	[illegible]
[illegible]	[illegible]	[illegible]	[illegible]	[illegible]	[illegible]
[illegible]	[illegible]				
[illegible]		[illegible]	[illegible]	[illegible]	[illegible]

	IPSC Amplitude				
**Figure 2c**	**dCre**	**Cre**	**Cre + Nrxn3a (-SS4)**	**Cre + Nrxn3a (LNS4-LNS6)**	**Cre + Nrxn3a (LNS1-LNS4)**
	1.878397583	0.372478027	3.935572998	0.084747612	5.267785645
	5.794429199	0.794836426	3.807510986	0.194543289	1.907627197
	0.894480286	1.380685059	1.49530481	0.446087555	0.6237901
	5.245019531	5.650089844	4.662871582	0.920987366	4.714319824
		0.591476196	7.262024902	0.118982117	7.95752832
	4.927504395	0.032868874	4.601295898	0.219041168	1.520331665
	4.504977051	0.122413185	1.351060913	1.0060495	2.651093018
	1.323639771	0.288438599	7.58114502	0.724992615	0.709278564
	4.025512695	0.040847393	5.081791504	1.107179688	2.391909668
	9.749810547	0.198694687	0.528076721	0.321287048	6.24501123
	0.553428223	0.257778564		0.076472359	9.093652344
	1.923399048	0.725104797		0.502667694	3.482282471
	6.204882813	0.943583984		0.090415604	2.928464355
	4.113476074	5.684670898		4.848932129	8.100895996
	5.459125	2.19826123		0.018875826	2.741332275
**Mean**	4.042720158	1.285481851	4.030665533	0.712084105	4.022353511
**Number (# cells)**	14	15	10	15	15
**Statistical Test**	ANOVA with Dunnett's Multiple comparisons test				
**Significance**		to dCre, ** or 0.0047	ns	to dCre, *** or 0.0005	ns

	IPSC Amplitude				
**Figure 2f**	**dCre**	**Cre**	**Cre + Nrxn3a (-SS4)**	**Cre + Nrxn3a (LNS2/3-only)**	**Cre + Nrxn3a (LNS2-only)**
	0.785692322	0.387728516	1.122364136	5.786537109	4.743918457
	3.587024902	1.143088867	6.007285645	1.671965454	11.80621484
	9.452959961	4.14034668	1.61983728	9.397269531	4.07598877
	4.60894043	0.044391834	5.159187012	6.176229492	4.21179541
	7.380458496	0.020140263	2.636102783	1.918957275	3.855185059
	12.42824121	7.956678711	2.636219971	12.20743262	2.10840332
	1.617584229	0.181040451	10.22994629	1.535411133	6.234746094
	5.631896973	1.97139856	6.554281738	2.549123291	4.081328613
	5.50968457	0.753565491	7.999151855	3.142222656	7.790749512
	7.648881348	2.853336914	2.290608398	5.764934082	12.49784766
	7.243889648	2.134730225	3.952739502	2.064831055	5.605489746
	1.673033813	0.082260223	0.716276001	4.443207031	4.660748047
	3.737975342	0.168303391	6.779841309	5.776748535	3.65961499
	3.903456055	2.590674805	3.476205566	1.791719849	6.68042041
		0.53862561	4.490854004	8.86315918	
		0.445560425	4.730633301		
		5.383587891	12.25826758		
		1.393002686	1.725601196		
		0.598703308	6.535405273		
		0.125680862	1.734318115		
		4.091167725	0.943911194		
		0.189941406	8.049019531		
		1.30984729			
		1.825361938			
		2.967205322			
		0.407705048			
		0.494904663			
		0.279454132			
		0.317594727			
		0.238563858			
**Mean**	5.372122807	1.501153061	4.620366258	4.872649886	5.858032209
**Number (# cells)**	14	30	22	15	14
**Statistical Test**	ANOVA with Dunnett's Multiple comparisons test				
**Significance**		to dCre, *** or 0.0003	ns	ns	ns

	IPSC Amplitude				
**Figure 2h**	**dCre**	**Cre**	**Cre + Nrxn3a (LNS2-only, -SS2)**	**Cre + Nrxn3a (LNS2-only, +SS2a)**	**Cre + Nrxn3a (LNS2-only, +SS2ab)**
	5964.409668	4606.71	7361.821289	3639.122803	3097.981934
	4745.520508	5399.395	11019.78516	3819.175049	7974.344238
	11629.30176	6389.247	7548.390625	1165.312012	8083.233398
	5912.60791	4517.534	5775.598145	4328.794922	5331.562988
	6221.105957		6929.060059	6349.186523	6319.923828
	13302.37695	536.1362	11310.85352	499.245544	266.343872
	4464.449707	1430.944	4805.038574	8412.263672	174.491028
	4620.115723	4690.004	8723.996094	298.91629	59.955173
				1489.80542	7368.539063
				2932.223877	3609.56665
	2691.438721	174.148	2283.496094	506.367737	2053.677246
	5187.203613	308.1678	1365.148071	1636.432861	629.925537
				411.405151	1003.198242
				570.505127	2115.99707
					699.965393
**Mean**	6473.853052	3116.920667	6712.318763	2575.625499	3252.580377
**Number (# cells)**	10	9	10	14	15
**Statistical Test**	ANOVA with Dunnett's Multiple comparisons test				
**Significance**		ns, p = 0.0511	ns	**, p = 0.0078	*, p = 0.0314

Figure 3e

Gephyrin Puncta Density, Avg. Mouse

	ΔCre	Cre	Cre + N3-L2 (SS2-)	Cre + N3-L2 (SS2ab)
	0.41575045	0.390080245	0.312931575	0.405016317
	0.47059792	0.411151178	0.442962728	0.445329132
	0.527587061	0.492753112	0.519471051	0.544329284
	0.557290105		0.55677772	0.490630199
	0.525490392		0.45413924	
**Mean**	0.499343186	0.431328178	0.457256463	0.471326233
**Number (# animals)**	5	3	5	4
**Statistical Test**	One-Way ANOVA with Tukey's Multiple comparisons test			
**Significance**	ns	ns	ns	ns

Synaptoporin Puncta Density, Avg. Mouse

	ΔCre	Cre	Cre + N3-L2 (SS2-)	Cre + N3-L2 (SS2ab)
	0.383518935	0.457873998	0.411570348	0.308552997
	0.410929424	0.295864549	0.389362215	0.343481464
	0.279541875	0.299965309	0.275481705	0.327842414
	0.263204624		0.364960929	0.264833474
	0.313737688		0.247342369	
**Mean**	0.330186509	0.351234619	0.337743513	0.311177587
**Number (# animals)**	5	3	5	4
**Statistical Test**	One-Way ANOVA with Tukey's Multiple comparisons test			
**Significance**		ns	ns	ns

Inh. Synapse Puncta Density, Avg. Mouse

	ΔCre	Cre	Cre + N3-L2 (SS2-)	Cre + N3-L2 (SS2ab)
	0.152955794	0.170527656	0.117548491	0.109650786
	0.185073468	0.115286629	0.191494241	0.151129673
	0.138270037	0.149206517	0.134013041	0.171808576
	0.151434443		0.09452829	0.11558629
	0.147357153		0.212354191	
**Mean**	0.155018179	0.145006934	0.149987651	0.137043831
**Number (# animals)**	5	3	5	4
**Statistical Test**	One-Way ANOVA with Tukey's Multiple comparisons test			
**Significance**	ns	ns	ns	ns

Gephyrin Puncta Density, Pseudoreplicate

	ΔCre	Cre	Cre + N3-L2 (SS2-)	Cre + N3-L2 (SS2ab)
	0.537651479	0.431108994	0.485791395	0.529184527
	0.510161593	0.495316717	0.529184527	0.578222293
	0.540473797	0.534829162	0.485438606	0.534123582
	0.538357058	0.464976804	0.46956307	0.5813974
	0.549293539	0.534829162	0.510486673	0.609973364
	0.578927872	0.475913284	0.472032598	0.556702122
	0.517542467	0.486144185	0.570813709	0.543296114
	0.484733026	0.478382812	0.587042035	0.55846607
	0.54788238	0.458979379	0.565521864	0.549293539
	0.540473797	0.511192253	0.548940749	0.518248046
	0.498491824	0.512956201	0.535181951	0.550351908
	0.509075515	0.479441181	0.548587959	0.506253197
	0.534476372	0.516836888	0.46921028	0.535534741
	0.532359634	0.464271225	0.484733026	0.46956307
	0.493905558	0.546118431	0.529537316	
	0.597978515		0.579986241	0.483321868
	0.551057487		0.524245471	0.591275511
	0.573636027		0.55952444	0.494582705
	0.57010813		0.577163924	0.484380237
	0.566227444		0.596567356	0.483321868
	0.537298689		0.542237745	0.445573371
	0.567285813		0.564816285	0.415586248
	0.571872078		0.5369459	0.537651479
	0.549293539		0.548940749	0.389479812
	0.484027447		0.556349332	0.520364785
	0.540826586		0.555996543	0.520011995
	0.541179376		0.541884955	0.545765642
	0.561993967		0.570813709	0.485085816
	0.570813709		0.539415427	0.480435368
	0.575752765			0.482616288
	0.55952444		0.403591399	
	0.516484098		0.500608562	
	0.516131308		0.463212856	
	0.540152011		0.396535605	
	0.556702122		0.4582738	
	0.541532166		0.439928736	
	0.520011995		0.460743328	
	0.533418003		0.422289252	
	0.546471221		0.490024872	
	0.504848298		0.455098693	
	0.509906345		0.485791395	
	0.506605987		0.519830713	
	0.50343088		0.457568221	
	0.594097829		0.454040324	
	0.498491824		0.454040324	
	0.460037748		0.404649768	
	0.410999982	0.389127022	0.198973382	0.370781958
	0.409941613	0.400769082	0.206381965	0.381365649
	0.455804272	0.398652343	0.321038613	0.397946764
	0.411705562	0.408883244	0.389127022	0.445220582
	0.406413716	0.42475878	0.376073804	0.432167363
	0.417702986	0.370429169	0.415586248	0.398153154
	0.416291828	0.416997407	0.241308144	0.409236034
	0.427933887	0.457215431	0.226490977	0.40253303
	0.427933887	0.431108994	0.244836041	0.381365649
	0.355964792	0.382071228	0.285406855	0.417350197
	0.338678097	0.328447196	0.345028311	0.404296978
	0.36901801	0.355445966	0.366548482	0.408883244
	0.468151911	0.384187966	0.357130038	0.421583673
	0.448748479	0.336561359	0.362315006	0.415939038
	0.446436653	0.366548482	0.35772874	0.388421443
	0.440281526			
	0.483321868	0.439223157	0.440634316	0.418408566
	0.45862659	0.441692685	0.470974229	0.474149336
	0.515778519	0.494963927	0.491083241	0.444867792
	0.451570796	0.474149336	0.412058351	0.448042899
	0.410647193	0.431461784	0.432520153	0.452629165
	0.470268649	0.456862641	0.419819724	0.34608668
	0.463565645	0.384187966	0.460743328	0.476971653
	0.489672082	0.356670371	0.323155351	0.455451483
	0.437811998	0.360903847	0.408177665	0.438870367
	0.474149336	0.344675522	0.455804272	0.44769011
	0.485438606	0.340442045	0.473796546	0.496375086
	0.456157062	0.419819724	0.444515002	0.454393114
	0.503783669	0.361609427	0.479088391	0.435342471
	0.51436736	0.449454058	0.499902983	
	0.443809423		0.432167363	
**Mean**	0.49859714	0.431786746	0.45588795	0.470957657
**Number (# ROI's)**	77	44	75	57
**Statistical Test**	One-Way ANOVA with Tukey's Multiple comparisons test			
**Significance**	ns	****, p < 0.0001 (to dCre)<br>*, p = 0.0369 (to Cre + LNS2ab)	**, p = 0.0019 (to dCre)	ns

Synaptoporin Puncta Density, Pseudoreplicate

	ΔCre	Cre	Cre + N3-L2 (SS2-)	Cre + N3-L2 (SS2ab)
	0.251186255	0.229313295	0.299871232	0.233546771
	0.325142023	0.32456651	0.284701275	0.328094407
	0.355612002	0.327036037	0.295637756	0.306574236
	0.274823164	0.295637756	0.22331587	0.36866522
	0.246599989	0.251539045	0.208145914	0.378543331
	0.347497839	0.229313295	0.228254926	0.428286677
	0.331975093	0.318921875	0.23531072	0.3693708
	0.297401704	0.258947628	0.182392267	0.3796017
	0.251891835	0.292462648	0.264239474	0.365490113
	0.288934752	0.258242049	0.34679226	0.269531319
	0.275528744	0.400769082	0.351025736	0.269178529
	0.206381965	0.342205994	0.338678097	0.276587113
	0.26882574	0.33550299	0.222963081	0.287876382
	0.257889259	0.348203419	0.263181105	0.328447196
	0.213437759	0.286818013	0.387715863	
	0.155227461		0.444162213	0.269178529
	0.407824875		0.454393114	0.240602565
	0.381012859		0.474854915	0.228212494
	0.237780247		0.468504701	0.251539045
	0.22331587		0.348203419	0.198973382
	0.274823164		0.343969942	0.143938191
	0.224727029		0.348556208	0.255066942
	0.249069517		0.282584537	0.210968231
	0.225432608		0.283995696	0.299518442
	0.176394842		0.305868656	0.224727029
	0.224727029		0.342205994	0.248716728
	0.246599989		0.372898696	0.333033462
	0.306927025		0.415586248	0.336208569
	0.284348486		0.22366866	0.443942121
	0.329858355			0.287876382
	0.30234076		0.20073733	
	0.285406855		0.231430033	
	0.338325307		0.243777672	
	0.316334083		0.209204283	
	0.363726165		0.225785398	
	0.460037748		0.243777672	
	0.405355347		0.190859219	
	0.341500415		0.22366866	
	0.390538181		0.228254926	
	0.259668951		0.389832601	
	0.254775257		0.34608668	
	0.215907287		0.3082214	
	0.273412005		0.206734755	
	0.305515867		0.244836041	
	0.26812016		0.244836041	
	0.238838616		0.219435184	
	0.40288582	0.490377661	0.524245471	0.305515867
	0.382424018	0.505194828	0.3796017	0.306221446
	0.452981955	0.453334744	0.41311672	0.26847295
	0.354906423	0.434284102	0.477677233	0.293873807
	0.340442045	0.476266074	0.391949339	0.282231748
	0.228607716	0.479441181	0.387363074	0.300623895
	0.295637756	0.451570796	0.375368224	0.396535605
	0.410999982	0.408530455	0.366901272	0.369723589
	0.43640084	0.427581098	0.393713288	0.287523593
	0.389832601	0.461448907	0.417702986	0.304810287
	0.360198268	0.453334744	0.445220582	0.317157926
	0.354553633	0.412764331	0.472385387	0.310807712
	0.467446332	0.484027447	0.314776928	0.33585578
	0.381718439	0.504489249	0.460390538	0.314335609
	0.435574448	0.425464359	0.353142474	0.23460514
	0.441692685			
	0.352084105	0.261417156	0.404649768	0.378896121
	0.366548482	0.339736466	0.376779383	0.372193117
	0.463212856	0.363373375	0.419466935	0.360198268
	0.416291828	0.340794835	0.35772874	0.239544196
	0.370429169	0.330563934	0.387363074	0.240249775
	0.396535605	0.408530455	0.347850629	0.188742481
	0.365842903	0.233899561	0.331622303	0.352789684
	0.497080665	0.185567374	0.218024025	0.318216295
	0.41346951	0.286465224	0.372545907	0.31257166
	0.378543331	0.256478101	0.434989681	0.348556208
	0.41311672	0.325624879	0.409941613	0.434989681
	0.400769082	0.313982819	0.349967367	0.506253197
	0.456157062	0.265650632	0.48049955	0.412058351
	0.481910709	0.230018874	0.492847189	
	0.391949339		0.456157062	
**Mean**	0.330665517	0.352493029	0.336175265	0.309751753
**Number (# ROI's)**	77	44	75	57
**Statistical Test**	One-Way ANOVA with Tukey's Multiple comparisons test			
**Significance**	ns	ns	ns	ns

Inh. Synapse Puncta Density, Pseudoreplicate

	ΔCre	Cre	Cre + N3-L2 (SS2-)	Cre + N3-L2 (SS2ab)
	0.124534759	0.090314159	0.143232612	0.120301282
	0.170174556	0.173219735	0.135471239	0.168986259
	0.184861795	0.174630894	0.134765659	0.155227461
	0.139704715	0.131590552	0.098781112	0.203912438
	0.132296132	0.121359651	0.086080683	0.222257501
	0.189448061	0.119242913	0.103014588	0.22366866
	0.174630894	0.166869521	0.113598278	0.175336473
	0.161577675	0.126298707	0.091372528	0.193681537
	0.14534935	0.147113298	0.137940767	0.21273218
	0.15663862	0.130532183	0.174278104	0.135824029
	0.140057505	0.207793124	0.194739906	0.128768235
	0.092783687	0.170750207	0.173572525	0.132296132
	0.149935616	0.171808576	0.095606004	0.158049779
	0.14534935	0.152405144	0.118537334	0.174278104
	0.103014588	0.154169092	0.209204283	
	0.075849782		0.26847295	0.124181969
	0.217318446		0.255772521	0.125593128
	0.2120266		0.300929601	0.103099214
	0.130884973		0.299518442	0.115362227
	0.121006862		0.21273218	0.074438623
	0.136882398		0.202148489	0.05574077
	0.116067806		0.20109012	0.101250639
	0.131590552		0.154169092	0.087139052
	0.104778536		0.152405144	0.098075532
	0.094194846		0.159460937	0.115362227
	0.11148154		0.185567374	0.11218712
	0.125240338		0.225785398	0.16722231
	0.163341624		0.2462472	0.148524457
	0.152405144		0.108659223	0.188843696
	0.180981108			0.116773386
	0.163694414		0.076202572	
	0.138646346		0.098075532	
	0.169691838		0.101250639	
	0.155142475		0.063149354	
	0.197209434		0.079024889	
	0.221551922		0.080436048	
	0.196151065		0.073733044	
	0.170044628		0.077260941	
	0.190859219		0.096311584	
	0.119729883		0.158755358	
	0.117068519		0.130884973	
	0.091372528		0.134103346	
	0.12241802		0.080788838	
	0.160166517		0.096664374	
	0.110775961		0.096664374	
	0.098428322		0.069146778	
	0.154521882	0.186625743	0.085727893	0.113245489
	0.153463513	0.194739906	0.081141627	0.1231236
	0.188742481	0.166869521	0.107248064	0.097017163
	0.133354501	0.17921716	0.164399993	0.099133901
	0.132296132	0.193328747	0.152757933	0.095606004
	0.079024889	0.164752783	0.147818878	0.104834821
	0.115362227	0.17886437	0.084316735	0.140410294
	0.171455787	0.170397418	0.085022314	0.127357076
	0.161577675	0.186625743	0.080788838	0.092078108
	0.136176818	0.174278104	0.103720167	0.107600854
	0.129121025	0.144643771	0.127004286	0.113598278
	0.131943342	0.140952711	0.154521882	0.117831755
	0.216965656	0.171808576	0.102293529	0.118537334
	0.162636045	0.15663862	0.160519306	0.115362227
	0.192261041	0.148171667	0.125945917	0.079024889
	0.188389691			
	0.160519306	0.114656647	0.195092695	0.14534935
	0.171455787	0.152052354	0.183450636	0.180981108
	0.202501279	0.177806001	0.22402145	0.159813727
	0.19015364	0.135471239	0.159460937	0.101956219
	0.186978533	0.127004286	0.197562223	0.10054506
	0.181686687	0.176042053	0.159813727	0.051860084
	0.161224886	0.082199996	0.150641195	0.15699141
	0.255066942	0.063502143	0.083258366	0.14570214
	0.184156215	0.087844631	0.176394842	0.136529608
	0.163341624	0.088903	0.21237939	0.14534935
	0.169339049	0.10089785	0.194387116	0.22366866
	0.160872096	0.117126175	0.152052354	0.236016299
	0.195445485	0.106895274	0.275528744	0.179922739
	0.229666085	0.083611155	0.266356212	
	0.163694414		0.242013724	
**Mean**	0.154944853	0.145682395	0.148416639	0.135939684
**Number (# ROI's)**	77	44	75	57
**Statistical Test**	One-Way ANOVA with Tukey's Multiple comparisons test			
**Significance**	ns	ns	ns	ns

Sex info
Male
Female

Figure 4b	ΔCre	Cre	Cre + N3-L2 (SS2-)	Cre + N3-L2 (SS2ab)
	[illegible]	[illegible]	[illegible]	[illegible]
	[illegible]	[illegible]	[illegible]	[illegible]
	[illegible]	[illegible]	[illegible]	[illegible]
	[illegible]	[illegible]	[illegible]	[illegible]
	[illegible]	[illegible]	[illegible]	[illegible]
	[illegible]	[illegible]	[illegible]	[illegible]
	[illegible]	[illegible]	[illegible]	[illegible]
	[illegible]	[illegible]	[illegible]	[illegible]
	[illegible]	[illegible]	[illegible]	[illegible]
	[illegible]	[illegible]	[illegible]	[illegible]
	[illegible]	[illegible]	[illegible]	[illegible]
	[illegible]	[illegible]	[illegible]	[illegible]
	[illegible]	[illegible]	[illegible]	[illegible]
	[illegible]	[illegible]	[illegible]	[illegible]
	[illegible]	[illegible]	[illegible]	[illegible]
	[illegible]	[illegible]	[illegible]	[illegible]
	[illegible]	[illegible]	[illegible]	[illegible]
	[illegible]	[illegible]	[illegible]	[illegible]
**Average**	6.938547203	2.975921813	6.272955093	3.247186118
**Count (cells)**	[illegible]	[illegible]	[illegible]	[illegible]
**Statistical Test**		one-way ANOVA with Tukey's Multiple comparisons test		
**Significance**		***, 0.0004 (to ΔCre)	ns, 0.9839 (to ΔCre)	**, 0.0012 (to ΔCre)

Figure 4c	ΔCre	Cre	Cre + N3-L2 (SS2-)	Cre + N3-L2 (SS2ab)
	[illegible]	[illegible]	[illegible]	[illegible]
	[illegible]	[illegible]	[illegible]	[illegible]
	[illegible]	[illegible]	[illegible]	[illegible]
	[illegible]	[illegible]	[illegible]	[illegible]
	[illegible]	[illegible]	[illegible]	[illegible]
	[illegible]	[illegible]	[illegible]	[illegible]
	[illegible]	[illegible]	[illegible]	[illegible]
	[illegible]	[illegible]	[illegible]	[illegible]
	[illegible]	[illegible]	[illegible]	[illegible]
	[illegible]	[illegible]	[illegible]	[illegible]
	[illegible]	[illegible]	[illegible]	[illegible]
	[illegible]	[illegible]	[illegible]	[illegible]
	[illegible]	[illegible]	[illegible]	[illegible]
	[illegible]	[illegible]	[illegible]	[illegible]
	[illegible]	[illegible]	[illegible]	[illegible]
	[illegible]	[illegible]	[illegible]	[illegible]
	[illegible]	[illegible]	[illegible]	[illegible]
	[illegible]	[illegible]	[illegible]	[illegible]
**Average**	74.40771997	68.14240709	74.39690604	61.90947909
**Count (cells)**	[illegible]	[illegible]	[illegible]	[illegible]
**Statistical Test**		one-way ANOVA with Tukey's Multiple comparisons test		
**Significance**		ns, 0.9816 (to ΔCre)	ns, >0.9999 (to ΔCre)	ns, 0.4602 (to ΔCre)

Figure 4e ΔCre	cell 1	cell 2	cell 3	cell 4	cell 5	cell 6	cell 7	cell 8	cell 9	cell 10	cell 11	cell 12	cell 13	cell 14	cell 15	cell 16	cell 17
5pA	[illegible]	[illegible]	[illegible]	[illegible]	[illegible]	[illegible]	[illegible]	[illegible]	[illegible]	[illegible]	[illegible]	[illegible]	[illegible]	[illegible]	[illegible]	[illegible]	[illegible]
10pA	[illegible]	[illegible]	[illegible]	[illegible]	[illegible]	[illegible]	[illegible]	[illegible]	[illegible]	[illegible]	[illegible]	[illegible]	[illegible]	[illegible]	[illegible]	[illegible]	[illegible]
20pA	[illegible]	[illegible]	[illegible]	[illegible]	[illegible]	[illegible]	[illegible]	[illegible]	[illegible]	[illegible]	[illegible]	[illegible]	[illegible]	[illegible]	[illegible]	[illegible]	[illegible]
30pA	[illegible]	[illegible]	[illegible]	[illegible]	[illegible]	[illegible]	[illegible]	[illegible]	[illegible]	[illegible]	[illegible]	[illegible]	[illegible]	[illegible]	[illegible]	[illegible]	[illegible]
40pA	[illegible]	[illegible]	[illegible]	[illegible]	[illegible]	[illegible]	[illegible]	[illegible]	[illegible]	[illegible]	[illegible]	[illegible]	[illegible]	[illegible]	[illegible]	[illegible]	[illegible]
50pA	[illegible]	[illegible]	[illegible]	[illegible]	[illegible]	[illegible]	[illegible]	[illegible]	[illegible]	[illegible]	[illegible]	[illegible]	[illegible]	[illegible]	[illegible]	[illegible]	[illegible]
75pA	[illegible]	[illegible]	[illegible]	[illegible]	[illegible]	[illegible]	[illegible]	[illegible]	[illegible]	[illegible]	[illegible]	[illegible]	[illegible]	[illegible]	[illegible]	[illegible]	[illegible]
100pA	[illegible]	[illegible]	[illegible]	[illegible]	[illegible]	[illegible]	[illegible]	[illegible]	[illegible]	[illegible]	[illegible]	[illegible]	[illegible]	[illegible]	[illegible]	[illegible]	[illegible]

Cre	cell 1	cell 2	cell 3	cell 4	cell 5	cell 6	cell 7	cell 8	cell 9	cell 10	cell 11	cell 12	cell 13	cell 14	cell 15	cell 16
5pA	[illegible]	[illegible]	[illegible]	[illegible]	[illegible]	[illegible]	[illegible]	[illegible]	[illegible]	[illegible]	[illegible]	[illegible]	[illegible]	[illegible]	[illegible]	[illegible]
10pA	[illegible]	[illegible]	[illegible]	[illegible]	[illegible]	[illegible]	[illegible]	[illegible]	[illegible]	[illegible]	[illegible]	[illegible]	[illegible]	[illegible]	[illegible]	[illegible]
20pA	[illegible]	[illegible]	[illegible]	[illegible]	[illegible]	[illegible]	[illegible]	[illegible]	[illegible]	[illegible]	[illegible]	[illegible]	[illegible]	[illegible]	[illegible]	[illegible]
30pA	[illegible]	[illegible]	[illegible]	[illegible]	[illegible]	[illegible]	[illegible]	[illegible]	[illegible]	[illegible]	[illegible]	[illegible]	[illegible]	[illegible]	[illegible]	[illegible]
40pA	[illegible]	[illegible]	[illegible]	[illegible]	[illegible]	[illegible]	[illegible]	[illegible]	[illegible]	[illegible]	[illegible]	[illegible]	[illegible]	[illegible]	[illegible]	[illegible]
50pA	[illegible]	[illegible]	[illegible]	[illegible]	[illegible]	[illegible]	[illegible]	[illegible]	[illegible]	[illegible]	[illegible]	[illegible]	[illegible]	[illegible]	[illegible]	[illegible]
75pA	[illegible]	[illegible]	[illegible]	[illegible]	[illegible]	[illegible]	[illegible]	[illegible]	[illegible]	[illegible]	[illegible]	[illegible]	[illegible]	[illegible]	[illegible]	[illegible]
100pA	[illegible]	[illegible]	[illegible]	[illegible]	[illegible]	[illegible]	[illegible]	[illegible]	[illegible]	[illegible]	[illegible]	[illegible]	[illegible]	[illegible]	[illegible]	[illegible]

Cre + N3-L2 (SS2-)	cell 1	cell 2	cell 3	cell 4	cell 5	cell 6	cell 7	cell 8	cell 9	cell 10	cell 11	cell 12	cell 13	cell 14
5pA	[illegible]	[illegible]	[illegible]	[illegible]	[illegible]	[illegible]	[illegible]	[illegible]	[illegible]	[illegible]	[illegible]	[illegible]	[illegible]	[illegible]
10pA	[illegible]	[illegible]	[illegible]	[illegible]	[illegible]	[illegible]	[illegible]	[illegible]	[illegible]	[illegible]	[illegible]	[illegible]	[illegible]	[illegible]
20pA	[illegible]	[illegible]	[illegible]	[illegible]	[illegible]	[illegible]	[illegible]	[illegible]	[illegible]	[illegible]	[illegible]	[illegible]	[illegible]	[illegible]
30pA	[illegible]	[illegible]	[illegible]	[illegible]	[illegible]	[illegible]	[illegible]	[illegible]	[illegible]	[illegible]	[illegible]	[illegible]	[illegible]	[illegible]
40pA	[illegible]	[illegible]	[illegible]	[illegible]	[illegible]	[illegible]	[illegible]	[illegible]	[illegible]	[illegible]	[illegible]	[illegible]	[illegible]	[illegible]
50pA	[illegible]	[illegible]	[illegible]	[illegible]	[illegible]	[illegible]	[illegible]	[illegible]	[illegible]	[illegible]	[illegible]	[illegible]	[illegible]	[illegible]
75pA	[illegible]	[illegible]	[illegible]	[illegible]	[illegible]	[illegible]	[illegible]	[illegible]	[illegible]	[illegible]	[illegible]	[illegible]	[illegible]	[illegible]
100pA	[illegible]	[illegible]	[illegible]	[illegible]	[illegible]	[illegible]	[illegible]	[illegible]	[illegible]	[illegible]	[illegible]	[illegible]	[illegible]	[illegible]

Cre + N3-L2 (SS2ab)	cell 1	cell 2	cell 3	cell 4	cell 5	cell 6	cell 7	cell 8	cell 9	cell 10	cell 11	cell 12	cell 13	cell 14	cell 15	cell 16	cell 17	cell 18	cell 19	cell 20
5pA	[illegible]	[illegible]	[illegible]	[illegible]	[illegible]	[illegible]	[illegible]	[illegible]	[illegible]	[illegible]	[illegible]	[illegible]	[illegible]	[illegible]	[illegible]	[illegible]	[illegible]	[illegible]	[illegible]	[illegible]
10pA	[illegible]	[illegible]	[illegible]	[illegible]	[illegible]	[illegible]	[illegible]	[illegible]	[illegible]	[illegible]	[illegible]	[illegible]	[illegible]	[illegible]	[illegible]	[illegible]	[illegible]	[illegible]	[illegible]	[illegible]
20pA	[illegible]	[illegible]	[illegible]	[illegible]	[illegible]	[illegible]	[illegible]	[illegible]	[illegible]	[illegible]	[illegible]	[illegible]	[illegible]	[illegible]	[illegible]	[illegible]	[illegible]	[illegible]	[illegible]	[illegible]
30pA	[illegible]	[illegible]	[illegible]	[illegible]	[illegible]	[illegible]	[illegible]	[illegible]	[illegible]	[illegible]	[illegible]	[illegible]	[illegible]	[illegible]	[illegible]	[illegible]	[illegible]	[illegible]	[illegible]	[illegible]
40pA	[illegible]	[illegible]	[illegible]	[illegible]	[illegible]	[illegible]	[illegible]	[illegible]	[illegible]	[illegible]	[illegible]	[illegible]	[illegible]	[illegible]	[illegible]	[illegible]	[illegible]	[illegible]	[illegible]	[illegible]
50pA	[illegible]	[illegible]	[illegible]	[illegible]	[illegible]	[illegible]	[illegible]	[illegible]	[illegible]	[illegible]	[illegible]	[illegible]	[illegible]	[illegible]	[illegible]	[illegible]	[illegible]	[illegible]	[illegible]	[illegible]
75pA	[illegible]	[illegible]	[illegible]	[illegible]	[illegible]	[illegible]	[illegible]	[illegible]	[illegible]	[illegible]	[illegible]	[illegible]	[illegible]	[illegible]	[illegible]	[illegible]	[illegible]	[illegible]	[illegible]	[illegible]
100pA	[illegible]	[illegible]	[illegible]	[illegible]	[illegible]	[illegible]	[illegible]	[illegible]	[illegible]	[illegible]	[illegible]	[illegible]	[illegible]	[illegible]	[illegible]	[illegible]	[illegible]	[illegible]	[illegible]	[illegible]

Average	Count	Statistical Test	Significance
[illegible] (ΔCre, 8 rows)	17	Two-way ANOVA with Dunnett Multiple comparisons test	
[illegible] (Cre, 8 rows)	16		****, <0.0001 (to deltaCre)
[illegible] (SS2-, 8 rows)	14		ns, 0.3688 (to deltaCre)
[illegible] (SS2ab, 8 rows)	20		****, <0.0001 (to deltaCre)

Figure 4f	ΔCre	Cre	Cre + N3-L2 (SS2-)	Cre + N3-L2 (SS2ab)
	[illegible]	[illegible]	[illegible]	[illegible]
	[illegible]	[illegible]	[illegible]	[illegible]
	[illegible]	[illegible]	[illegible]	[illegible]
	[illegible]	[illegible]	[illegible]	[illegible]
	[illegible]	[illegible]	[illegible]	[illegible]
	[illegible]	[illegible]	[illegible]	[illegible]
	[illegible]	[illegible]	[illegible]	[illegible]
	[illegible]	[illegible]	[illegible]	[illegible]
	[illegible]	[illegible]	[illegible]	[illegible]
	[illegible]	[illegible]	[illegible]	[illegible]
	[illegible]	[illegible]	[illegible]	[illegible]
	[illegible]	[illegible]	[illegible]	[illegible]
	[illegible]	[illegible]	[illegible]	[illegible]
	[illegible]	[illegible]	[illegible]	[illegible]
	[illegible]	[illegible]		[illegible]
	[illegible]	[illegible]		[illegible]
	[illegible]			[illegible]
				[illegible]
				[illegible]
				[illegible]
**Average**	21.38535508	11.44220292	19.11623734	12.81272388
**Count (cells)**	17	16	14	20
**Statistical Test**		one-way ANOVA with Tukey's Multiple comparisons test		
**Significance**		**, 0.0036 (to ΔCre)	ns, 0.9007 (to ΔCre)	**, 0.0089 (to ΔCre)

Figure 4g	rise time				decay time			
	ΔCre	Cre	Cre + N3-L2 (SS2-)	Cre + N3-L2 (SS2ab)	ΔCre	Cre	Cre + N3-L2 (SS2-)	Cre + N3-L2 (SS2ab)
	[illegible]	[illegible]	[illegible]	[illegible]	[illegible]	[illegible]	[illegible]	[illegible]
	[illegible]	[illegible]	[illegible]	[illegible]	[illegible]	[illegible]	[illegible]	[illegible]
	[illegible]	[illegible]	[illegible]	[illegible]	[illegible]	[illegible]	[illegible]	[illegible]
	[illegible]	[illegible]	[illegible]	[illegible]	[illegible]	[illegible]	[illegible]	[illegible]
	[illegible]	[illegible]	[illegible]	[illegible]	[illegible]	[illegible]	[illegible]	[illegible]
	[illegible]	[illegible]	[illegible]	[illegible]	[illegible]	[illegible]	[illegible]	[illegible]
	[illegible]	[illegible]	[illegible]	[illegible]	[illegible]	[illegible]	[illegible]	[illegible]
	[illegible]	[illegible]	[illegible]	[illegible]	[illegible]	[illegible]	[illegible]	[illegible]
	[illegible]	[illegible]	[illegible]	[illegible]	[illegible]	[illegible]	[illegible]	[illegible]
	[illegible]	[illegible]	[illegible]	[illegible]	[illegible]	[illegible]	[illegible]	[illegible]
	[illegible]	[illegible]	[illegible]	[illegible]	[illegible]	[illegible]	[illegible]	[illegible]
	[illegible]	[illegible]	[illegible]	[illegible]	[illegible]	[illegible]	[illegible]	[illegible]
	[illegible]	[illegible]	[illegible]	[illegible]	[illegible]	[illegible]	[illegible]	[illegible]
	[illegible]	[illegible]	[illegible]	[illegible]	[illegible]	[illegible]	[illegible]	[illegible]
	[illegible]	[illegible]		[illegible]	[illegible]	[illegible]		[illegible]
	[illegible]	[illegible]		[illegible]	[illegible]	[illegible]		[illegible]
	[illegible]			[illegible]	[illegible]			[illegible]
				[illegible]				[illegible]
				[illegible]				[illegible]
				[illegible]				[illegible]
**Average**	1.389267833	1.304359663	1.524182754	1.403252313	16.04197648	16.13340008	16.65209416	16.41611013
**Count (cells)**	17	16	14	20	17	16	14	20
**Statistical Test**		one-way ANOVA with Tukey's Multiple comparisons test				one-way ANOVA with Tukey's Multiple comparisons test		
**Significance**		ns, 0.9539 (to ΔCre)	ns, 0.8551 (to ΔCre)	ns, 0.9997 (to ΔCre)		ns, 0.9997 (to ΔCre)	ns, 0.9669 (to ΔCre)	ns, 0.9883 (to ΔCre)

Figure 4h	ΔCre	Cre	Cre + N3-L2 (SS2-)	Cre + N3-L2 (SS2ab)
	[illegible]	[illegible]	[illegible]	[illegible]
	[illegible]	[illegible]	[illegible]	[illegible]
	[illegible]	[illegible]	[illegible]	[illegible]
	[illegible]	[illegible]	[illegible]	[illegible]
	[illegible]	[illegible]	[illegible]	[illegible]
	[illegible]	[illegible]	[illegible]	[illegible]
	[illegible]	[illegible]	[illegible]	[illegible]
	[illegible]	[illegible]	[illegible]	[illegible]
	[illegible]	[illegible]	[illegible]	[illegible]
	[illegible]	[illegible]	[illegible]	[illegible]
	[illegible]	[illegible]	[illegible]	[illegible]
	[illegible]	[illegible]	[illegible]	[illegible]
	[illegible]	[illegible]	[illegible]	[illegible]
	[illegible]	[illegible]	[illegible]	[illegible]
	[illegible]	[illegible]		[illegible]
	[illegible]	[illegible]		[illegible]
	[illegible]			[illegible]
				[illegible]
				[illegible]
				[illegible]
**Average**	0.198993426	0.388937634	0.218393655	0.365056757
**Count (cells)**	17	16	14	20
**Statistical Test**		one-way ANOVA with Tukey's Multiple comparisons test		
**Significance**		***, 0.0003 (to ΔCre)	ns, 0.9233 (to ΔCre)	***, 0.0009 (to ΔCre)

Figure 4i ΔCre	cell 1	cell 2	cell 3	cell 4	cell 5	cell 6	cell 7	cell 8	cell 9	cell 10	cell 11	cell 12	cell 13	cell 14
PPR 20ms	[illegible]	[illegible]	[illegible]	[illegible]	[illegible]	[illegible]	[illegible]	[illegible]	[illegible]	[illegible]	[illegible]	[illegible]	[illegible]	[illegible]
PPR 50ms	[illegible]	[illegible]	[illegible]	[illegible]	[illegible]	[illegible]	[illegible]	[illegible]	[illegible]	[illegible]	[illegible]	[illegible]	[illegible]	[illegible]
PPR 100ms	[illegible]	[illegible]	[illegible]	[illegible]	[illegible]	[illegible]	[illegible]	[illegible]	[illegible]	[illegible]	[illegible]	[illegible]	[illegible]	[illegible]
PPR 500ms	[illegible]	[illegible]	[illegible]	[illegible]	[illegible]	[illegible]	[illegible]	[illegible]	[illegible]	[illegible]	[illegible]	[illegible]	[illegible]	[illegible]

Cre	cell 1	cell 2	cell 3	cell 4	cell 5	cell 6	cell 7	cell 8	cell 9	cell 10	cell 11	cell 12	cell 13
PPR 20ms	[illegible]	[illegible]	[illegible]	[illegible]	[illegible]	[illegible]	[illegible]	[illegible]	[illegible]	[illegible]	[illegible]	[illegible]	[illegible]
PPR 50ms	[illegible]	[illegible]	[illegible]	[illegible]	[illegible]	[illegible]	[illegible]	[illegible]	[illegible]	[illegible]	[illegible]	[illegible]	[illegible]
PPR 100ms	[illegible]	[illegible]	[illegible]	[illegible]	[illegible]	[illegible]	[illegible]	[illegible]	[illegible]	[illegible]	[illegible]	[illegible]	[illegible]
PPR 500ms	[illegible]	[illegible]	[illegible]	[illegible]	[illegible]	[illegible]	[illegible]	[illegible]	[illegible]	[illegible]	[illegible]	[illegible]	[illegible]

Cre + N3-L2 (SS2-)	cell 1	cell 2	cell 3	cell 4	cell 5	cell 6	cell 7	cell 8	cell 9	cell 10
PPR 20ms	[illegible]	[illegible]	[illegible]	[illegible]	[illegible]	[illegible]	[illegible]	[illegible]	[illegible]	[illegible]
PPR 50ms	[illegible]	[illegible]	[illegible]	[illegible]	[illegible]	[illegible]	[illegible]	[illegible]	[illegible]	[illegible]
PPR 100ms	[illegible]	[illegible]	[illegible]	[illegible]	[illegible]	[illegible]	[illegible]	[illegible]	[illegible]	[illegible]
PPR 500ms	[illegible]	[illegible]	[illegible]	[illegible]	[illegible]	[illegible]	[illegible]	[illegible]	[illegible]	[illegible]

Cre + N3-L2 (SS2ab)	cell 1	cell 2	cell 3	cell 4	cell 5	cell 6	cell 7	cell 8	cell 9	cell 10	cell 11	cell 12	cell 13	cell 14	cell 15	cell 16	cell 17
PPR 20ms	[illegible]	[illegible]	[illegible]	[illegible]	[illegible]	[illegible]	[illegible]	[illegible]	[illegible]	[illegible]	[illegible]	[illegible]	[illegible]	[illegible]	[illegible]	[illegible]	[illegible]
PPR 50ms	[illegible]	[illegible]	[illegible]	[illegible]	[illegible]	[illegible]	[illegible]	[illegible]	[illegible]	[illegible]	[illegible]	[illegible]	[illegible]	[illegible]	[illegible]	[illegible]	[illegible]
PPR 100ms	[illegible]	[illegible]	[illegible]	[illegible]	[illegible]	[illegible]	[illegible]	[illegible]	[illegible]	[illegible]	[illegible]	[illegible]	[illegible]	[illegible]	[illegible]	[illegible]	[illegible]
PPR 500ms	[illegible]	[illegible]	[illegible]	[illegible]	[illegible]	[illegible]	[illegible]	[illegible]	[illegible]	[illegible]	[illegible]	[illegible]	[illegible]	[illegible]	[illegible]	[illegible]	[illegible]

Average	Count	Statistical Test	Significance
[illegible] (ΔCre, 4 rows)	14	Two-way ANOVA with Dunnett Multiple comparisons test	
[illegible] (Cre, 4 rows)	13		****, 0.0001 (to deltaCre)
[illegible] (SS2-, 4 rows)	10		0.9932 (to deltaCre)
[illegible] (SS2ab, 4 rows)	17		****, <0.0001 (to deltaCre)

Sex info
Male
Female

Figure 5d	ΔCre	Cre	Cre + N3-L2 (SS2-)
	33.08665537	20.27520154	11.68815989
	22.77766001	19.79623029	24.04925212
	16.97379736	11.40458955	17.30123684
	35.13991623	17.22425327	20.50749234
	16.86132199	11.78101486	29.05597039
	11.20778883	7.635044856	16.18437203
	29.57005031	14.18084305	22.06644975
	9.656517788	9.175799355	12.06718974
	16.51654994	5.871967708	11.03018241
	8.059706473	8.209237209	17.95375156
	11.29492573	5.328606415	9.077870398
	38.33180054	11.12854616	13.09220381
	17.26966583	26.02585235	7.943947178
	10.1939918	15.15848163	15.60338313
	18.11080274	9.348153159	18.40795737
	9.176556956	9.420838035	34.73227207
	9.896645772	19.38673301	9.620996623
	21.47950885	13.30023054	11.05057834
	13.2832697	10.08081453	25.45954394
	14.70163013	8.40908536	21.24014074
	17.33252448	17.45007784	13.76058394
	10.9260962	6.302057622	9.105588187
**Average**	17.81124468	12.58607538	16.86359649
**Count (cells)**	22	22	22
**Statistical Test**	one-way ANOVA with Tukey's Multiple comparisons test		
**Significance**		*, 0.0442 (to ΔCre)	ns, 0.9624 (to ΔCre)

Figure 5e	ΔCre	Cre	Cre + N3-L2 (SS2-)
	55.72067444	30.72271545	37.07911214
	47.53241618	29.48103353	35.58536418
	30.5562733	37.10095703	25.12172892
	51.89694763	38.51826538	42.05912964
	46.39984273	42.01057983	33.78859517
	41.56410889	30.19556217	31.140631
	33.93053813	37.88191233	25.62493591
	26.85420512	39.67763916	23.11932699
	54.60321676	17.6805364	41.00677205
	32.1289684	39.67808695	46.18389832
	35.29991699	16.85451619	40.50687876
	61.7972583	32.60593892	31.76019409
	46.35625732	39.55006958	38.14988688
	40.95822717	28.67827108	27.92633606
	50.86942769	46.00581523	30.58007874
	31.07650085	27.36680176	39.87721415
	43.50107402	25.22674744	25.28307821
	41.71091268	30.07298574	33.20721578
	31.35516073	39.8756488	34.53149028
	33.74540609	33.67227804	34.87043172
	26.68144836	26.77633565	29.4364327
	32.63278117	37.90568156	39.22144511
**Average**	40.78052559	33.06992628	33.91182622
**Count (cells)**	22	22	22
**Statistical Test**	one-way ANOVA with Tukey's Multiple comparisons test		
**Significance**		**, 0.0070 (to ΔCre)	*, 0.0185 (to ΔCre)

Figure 5g	ΔCre	cell 1	cell 2	cell 3	cell 4	cell 5	cell 6	cell 7	cell 8	cell 9	cell 10	cell 11	cell 12	cell 13	cell 14	cell 15	cell 16	Average	Count	Statistical Test	Significance
	5uA	0.00869751	0.018030556	0.025964685	0.004245924	0.013997396	0.042272949	0	0.05201392	0.264009918	0	0	0.033761162	0	0.046159225	0	0.020560129	0.033107086	16		
	10uA	0.504306946	1.495073608	1.153765747	0.962425415	0.088355812	2.301730713	0.448645966	2.459514404	4.767181641	0.337881226	0.79303717	0.125297035	3.77070752	0.166915894	0.027583008	2.106986572	1.344338042	16		
	15uA	0.798132996	3.857144531	2.598949463	3.018617432	0.515710754	3.009377686	2.89542334	3.473037598	5.259799316	4.374201172	3.996394775	1.653526489	8.020148926	2.256633057	0.036665752	4.413043457	3.136050422	16		
	20uA	1.431837402	4.124155273	3.231218018	3.685756104	1.111239014	3.264994873	4.189687988	4.64798584	6.137756348	4.820350586	6.031804688	1.88698938	8.90043457	4.428713867	4.205271973	4.860915527	4.184944466	16		
	25uA	2.095214844	5.492344727	5.936523438	4.329061035	2.147388428	2.75896875	6.119327148	4.072883789	6.771738281	4.812659668	7.87084375	1.027388184	9.477169922	3.860094971	3.620283936	6.767128906	4.822438736	16		
	30uA	2.468828857	6.082618164	7.338557617	5.749165527	2.774387695	2.801755615	6.102416992	3.95893335	6.880056152	5.371413574	8.399644531	3.085812744	9.71675293	3.901509521	4.044665527	6.623270508	5.331236832	16		
	40uA	4.413566406	5.170899902	8.593271484	7.502909668	3.964625977	2.958983154	7.117675781	4.37536377	6.497514648	4.905714844	7.943739746	3.053729492	8.476850586	4.594478516	4.358313477	6.561964844	5.655600143	16		
	50uA	4.935589844	7.180820801	9.481775391	7.692547363	5.073556152	3.262817871	6.137160156	4.228392578	6.683088867	4.685387207	7.74375	3.140893066	8.65812207	4.410916504	4.202091309	7.013630371	5.908158722	16		
	**Cre**	cell 1	cell 2	cell 3	cell 4	cell 5	cell 6	cell 7	cell 8	cell 9	cell 10	cell 11	cell 12	cell 13	cell 14	cell 15	cell 16				
	5uA	0.105557526	0	0.249742294	0.02846854	0	0.066156158	0.109959656	0.098388672	0	0	0.096153259	0	0	0	0	0.130205658	0.055289485	16		
	10uA	0.847917847	0.056711834	0.989045715	2.731400391	0.79934198	0.191226502	0.484249725	0.250015595	0.216158356	0.730644043	0.64258374	0.0625	0.140489929	0.032413738	1.746132446	2.234503174	0.759708438	16		
	15uA	1.076224243	1.782363892	1.088324097	3.279325684	1.342871094	1.252939575	0.829856995	0.60889148	1.284827637	2.416580078	3.529101074	1.337161377	0.325493805	0.501796753	2.054075928	2.689841553	1.587479704	16		
	20uA	2.968005859	1.607110596	1.574232178	3.447105469	1.601904663	2.64439502	1.286716675	1.12503418	2.81557959	4.355733398	3.932210693	1.720812622	2.861818359	0.947172058	4.035453125	4.142020508	2.566581562	16	Two-way ANOVA with Dunnett Multiple comparisons test	****, <0.0001 (to ΔCre)
	25uA	3.617193359	1.483201294	1.589385498	3.976438232	1.276062012	3.521484619	1.470496094	1.437540649	2.439744141	4.354559082	4.207924316	1.778860229	2.768212402	1.01932782	3.297922119	6.155692871	2.774627796	16		
	30uA	3.664765869	1.373370972	1.794380859	3.500243164	1.161647583	2.973595215	1.889885376	1.212772461	2.364945313	4.319019531	3.026588623	1.310377808	3.73182251	1.032688354	3.275404785	6.237675781	2.679324013	16		
	40uA	4.250709473	1.42716626	2.24791748	3.337801758	1.569291992	3.478177734	2.210175049	1.957796509	2.259200928	4.828723633	3.17591333	1.381532349	3.316103271	1.145548462	3.325061768	6.613478516	2.907787407	16		
	50uA	4.15723584	1.585543091	2.336954346	3.280918945	1.958566772	3.517077881	2.298072021	1.994413696	3.096338379	6.848422363	2.104624512	1.604365479	3.418854492	1.127203369	3.965078613	6.597674805	3.118209038	16		
	**Cre + N3-L2 (SS2-)**	cell 1	cell 2	cell 3	cell 4	cell 5	cell 6	cell 7	cell 8	cell 9	cell 10	cell 11	cell 12	cell 13	cell 14	cell 15					
	5uA	0.052777325	0.139853348	0.009943782	0.004118652	0.025966099	0.113244629	0	0.982086731	0.13114827	0.16907959	0.060058594	0	0	0	0.438937714		0.141814315	15		
	10uA	0.732871704	2.331780273	0.203233872	0.494167023	0.511092377	1.935807251	0.933540833	3.074328613	0.658398438	1.376643311	0.166745865	0.07131723	3.548142822	0.165002899	3.173278076		1.291756706	15		
	15uA	1.479908447	4.405369629	1.670439331	0.731253967	3.673931152	2.305904053	1.278975464	6.762988281	3.028836914	3.068199463	1.668776245	0.297861725	4.501173828	0.133721252	4.998974121		2.667087592	15		
	20uA	1.9899021	7.880351074	2.420779785	1.841532959	4.745338379	2.70847583	2.612434814	10.17621289	3.494921875	2.795551025	3.257821777	4.26559668	8.701650391	1.195543091	4.975330078		4.204096183	15		ns, 0.6555 (to ΔCre)
	25uA	2.848230713	8.473379883	4.189586914	3.112232422	5.174488281	2.478792236	3.044799805	10.00442676	5.641194824	3.092251953	4.080453613	3.753511963	9.802490234	1.001812988	4.806583496		4.766949072	15		
	30uA	3.281430908	8.478006836	4.272862305	3.959835449	5.006510254	2.339782715	3.021976807	9.759273438	6.369456055	3.148259277	3.789973877	4.206787109	10.19152832	1.255447998	6.743647461		5.054985254	15		
	40uA	3.61738916	9.325978516	8.146677246	4.493507324	5.10253418	2.002652954	2.611755371	9.606818359	6.538146973	3.405451904	3.980318604	4.062166748	9.867807617	1.591471313	4.595881348		5.263237174	15		
	50uA	4.655300781	9.468817383	8.013209473	4.672678223	5.572425293	2.491455078	2.945333008	11.1617666	6.118855957	3.206054688	3.999692627	5.015186523	10.17522754	1.724785645	5.886253906		5.673802848	15		

Figure 5h	ΔCre	Cre	Cre + N3-L2 (SS2-)
	11.68206085	9.773437148	9.983163093
	13.95954387	2.899435782	20.30585799
	22.60592812	4.312806518	20.34177967
	17.94573302	4.486203859	11.84258889
	12.18758736	3.141547713	11.7679947
	4.372985882	8.388615369	2.858218761
	15.70437448	5.084910556	6.06366142
	6.922495037	4.518664667	20.10821642
	10.22913233	6.289057219	14.48635023
	10.04903231	13.91851617	5.60751494
	18.46421884	3.811656729	9.562705737
	7.398046439	3.096298452	12.05487527
	15.20233784	8.912671561	21.40151521
	10.1950954	2.707703315	4.186287173
	11.20017553	6.907552041	8.683177929
	14.20672116	14.38784931	
**Average**	12.64534178	6.414807901	11.9502605
**Count (cells)**	16	16	15
**Statistical Test**	one-way ANOVA with Tukey's Multiple comparisons test		
**Significance**		**, 0.0026 (to ΔCre)	ns, 0.9197 (to ΔCre)

Figure 5i	rise time					decay time		
	ΔCre	Cre	Cre + N3-L2 (SS2-)			ΔCre	Cre	Cre + N3-L2 (SS2-)
	1.920768023	1.322306633	1.934096575			77.30950165	40.71490097	47.63690186
	1.597219706	4.384708881	1.400321841			49.55550003	78.2141037	64.3864975
	1.35463953	2.07113	0.881232262			58.18799973	65.771	57.29650116
	1.275668502	3.742317438	1.846053004			82.02749634	93.44529724	84.49199677
	1.210665464	4.044305325	2.174274921			92.62010193	71.98090363	47.42079926
	2.540911436	3.049522161	2.121698141			99.76709747	77.40070343	57.66049957
	2.110756159	1.919538498	2.222308636			47.28630066	31.5583992	54.37779999
	2.638007879	2.251010656	1.726323366			83.39089966	53.66659927	65.07489777
	1.838764191	2.465481281	2.8472929			49.50579834	55.51190186	73.72327423
	2.107265949	2.065537214	2.625669479			42.36880112	43.44979858	56.34550095
	1.50511539	5.772886276	2.074166536			52.31750107	109.2099991	37.2378006
	3.340965986	2.854610682	2.750765085			66.00050354	68.94550323	70.1835022
	1.600717783	1.574673772	1.587146282			50.24029922	31.82299995	42.97999954
	4.41513586	3.388375759	4.499539852			57.52420044	54.14020157	71.90920258
	0.729940057	4.520892143	1.362264514			69.58820343	129.3880005	30.12750053
	2.211130142	1.425482869				33.32820129	42.39070129	
**Average**	2.024854504	2.928298724	2.136876893		**Average**	63.18865037	65.47568835	57.3901783
**Count (cells)**	16	16	15		**Count (cells)**	16	16	15
**Statistical Test**	one-way ANOVA with Tukey's Multiple comparisons test				**Statistical Test**	one-way ANOVA with Tukey's Multiple comparisons test		
**Significance**		*, 0.0323 (to ΔCre)	ns, 0.9361 (to ΔCre)		**Significance**		ns, 0.9354 (to ΔCre)	ns, 0.6707 (to ΔCre)

Figure 5j	ΔCre	Cre	Cre + N3-L2 (SS2-)
	0.113337342	0.068451696	0.045371897
	0.138923405	0.238515657	0.024565276
	0.050349036	0.154278375	0.051207975
	0.15312625	0.135442164	0.122252264
	0.079559307	0.114925828	0.108946908
	0.094557377	0.129556867	0.15781886
	0.277126487	0.366294653	0.103197537
	0.092199881	0.485610369	0.041884933
	0.104090418	0.069876757	0.119559277
	0.080135851	0.206561537	0.075411384
	0.053088968	0.48366384	0.154039671
	0.113963948	0.467731097	0.149033647
	0.036759723	0.177826308	0.048125623
	0.131240028	0.214367571	0.214016572
	0.064644373	0.104129118	0.14229068
	0.047542857	0.069427341	
**Average**	0.101915328	0.217916199	0.103848167
**Count (cells)**	16	16	15
**Statistical Test**	one-way ANOVA with Tukey's Multiple comparisons test		
**Significance**		**, 0.0051 (to ΔCre)	ns, 0.9984 (to ΔCre)

Figure 6a	Ctrl	Dag1 gRNA
	100	39.78243606
	100	41.18925945
	100	31.15536592
**Average**	100	37.37568714
**Count (cultures)**	3	3
**Statistical Test**	two-tailed, one sample t test	
**T-value**	19.97	
**df**	2	
**Significance**	**, p = 0.0025	

Figure 6c	Ctrl	Dag1 gRNA
	1.393716675	4.538832031
	2.448588867	0.374032013
	1.557436279	0.319219849
	6.824246094	1.741697021
	3.690708008	0.605207947
	1.576948242	0.570067383
	5.698196777	2.974365234
	2.13142041	1.538900391
	4.416081543	3.182121338
	4.263709961	0.683313416
	0.848329895	2.760336914
	1.689602783	1.110234009
	8.952677734	0.441584839
**Average**	3.499358713	1.603070183
**Count (cells)**	13	13
**Statistical Test**	unpaired two-tailed T-test with Welch's correction	
**T-value**	2.439	
**df**	18.69	
**Significance**	*, p = 0.0249	

Figure 6e	dCre	dCre + Dag1 gRNA	Cre	Cre + Dag1 gRNA
	1.51827417	2.10462793	2.110289795	3.119434814
	3.787624268	0.245582733	0.366818573	4.967742188
		0.665654175	2.347228271	0.922079834
		0.498255951	1.312861328	1.540783447
		0.285121552	1.465384644	0.973251953
	5.331669922	0.73066095	8.572755859	3.184894531
	4.191088867	0.601045288	1.769262939	5.042607422
	5.69667334	0.25892804	2.786459473	1.875074219
	0.689091675	4.245503906	2.910873047	
	9.865595703		0.152849976	
	4.40634668	3.223001221	2.037180176	5.77991748
	9.017279297	2.340318848	1.687684937	4.436753906
	6.398844238	0.498766571	2.844393311	5.753077637
	5.571657715	4.732151367	6.844233398	0.699419678
	6.601068848	2.016201172	0.380742035	0.409260254
**Average**	5.256267894	1.603272836	2.505934517	2.977253643
**Count (cells)**	12	14	15	13
**Statistical Test**	2-way ANOVA with Tukey's multiple comparisons test	***, p = 0.0004 (to dCre)	**, p = 0.0096 (to dCre)	ns, p = 0.0521 (to dCre)
**Significance**				

Figure 6f	dCre	dCre + Dag1 gRNA	Cre	Cre + Dag1 gRNA
	0.07399437	0.152970875	0.105920813	0.189186156
	0.240058	0.078600055	0.023582816	0.343229219
		0.276714656	0.235149234	0.049740852
		0.094741336	0.083423867	0.088756281
		0.024394848	0.062349836	0.097094117
	1.113047	0.07453825	0.505035844	0.236606031
	0.2177181	0.060650957	0.077243258	0.44477025
	0.430611	0.008706488	0.203309938	0.255611781
	0.03278019	0.341865844	0.05439418	
	1.353603		0.013444664	
	0.3251208	0.328706344	0.149212828	0.57552875
	0.7693861	0.150327672	0.073906609	0.320148438
	0.3028154	0.027874043	0.138047297	0.482904125
	0.3486759	0.408728156	0.406146375	0.046889703
	0.2724544	0.211029719	0.013951248	
**Average**	0.456688688	0.159989232	0.14300792	0.260872142
**Count (cells)**	12	14	15	12
**Statistical Test**	2-way ANOVA with Tukey's multiple comparisons test			
**Significance**		*, p = 0.0123 (to dCre)	**, p = 0.0063 (to dCre)	ns, p = 4023 (to dCre)

	NMDAR-EPSCs			
Figure 6h	dCre	dCre + Dag1 gRNA	Cre	Cre + Dag1 gRNA
	0.37112735	1.83625	0.813398804	1.076017822
	0.694535767	0.148152313	2.626472412	0.581522644
	0.816284912	0.905649109	0.617216675	1.569328369
	1.315511841	1.646802612	0.250404358	0.532439087
	0.057595222	0.092072693	0.078903465	0.045541519
	0.128634232	0.048939213	0.35528775	0.071145027
	0.096125938	0.093399841		0.185211075
	0.023359358	0.006191372	0.050013329	0.325438599
	0.167145096	0.008552102	0.015279064	0.11013488
	0.095614426	0.093797729	0.306264832	0.059980179
	0.075727943			
**Average**	0.349242008	0.487980698	0.568137854	0.45567592
**Count (cells)**	11	10	9	10
**Statistical Test**	2-way ANOVA with Tukey's multiple comparisons test			
**Significance**		ns	ns	ns

	AMPAR-EPSCs			
Figure 6i	dCre	dCre + Dag1 gRNA	Cre	Cre + Dag1 gRNA
	2.358732666	2.915829346	0.583675903	0.979468201
	0.658214722	2.160535156	1.657297363	2.89213501
	1.105677612	1.045551147	5.009924805	1.766452637
	0.75769989	2.304394287	0.848229553	1.108194214
	0.741980042	0.805942688	0.534341003	1.164858887
	0.616252441	0.244198593	0.37730896	1.044040039
	0.30530304	0.283946869	0.644206543	0.468593994
	0.294131012	0.545852234	0.24519519	0.104488419
		0.32795343	1.767139771	1.126227783
	0.883407043	0.689507324	0.829792236	0.425960571
	0.634854187		0.313576599	
	0.922574707			
**Average**	0.84352976	1.132371107	1.164607993	1.108041976
**Count (cells)**	11	10	11	10
**Statistical Test**	2-way ANOVA with Tukey's multiple comparisons test			
**Significance**		ns	ns	ns

Sex info

Male (yellow)

Female (grey)

Figure 8d	Gephyrin Puncta Density, Avg. per Animal Ctrl	Dag1 gRNA
	0.43640084	0.436584029
	0.449365861	0.425237566
	0.455509494	0.49300013
**Mean**	0.447092065	0.451607242
**Number (# cells)**	3	3
**Statistical Test**	two-tailed unpaired Welch's t-test	
**t-value**	0.2081	
**df**	2.287	
**Significance**	ns, p = 0.8522	

Figure 8d	Gephyrin Puncta Size, Avg. per Animal Ctrl	Dag1 gRNA
	0.186247542	0.194552
	0.189311989	0.184149
	0.175112783	0.193998
**Mean**	0.183557438	0.190899667
**Number (# cells)**	3	3
**Statistical Test**	two-tailed unpaired Welch's t-test	
**t-value**	1.34	
**df**	3.783	
**Significance**	ns, p = 0.2551	

Figure 8g	Ctrl	Dag1 gRNA
	3.909635296	2.299516587
	3.595656447	2.995040167
	4.35858981	3.959439665
	6.064980102	5.921750087
	8.087474461	1.3082139
	4.036191181	3.380620645
	3.625272568	1.69367998
	7.973655375	4.521415792
	6.793216867	3.816750266
	3.052829209	1.920266725
	5.200956353	8.245019654
	3.582337509	4.316385189
	4.444648672	1.461520339
	5.538836475	3.881500224
	7.491889883	3.335004599
	6.13878547	2.146423176
	2.407368504	2.522093606
	3.555799852	1.827618978
	7.421260714	1.341267677
		1.150447012
**Mean**	5.119967618	3.102198713
**Number (# cells)**	19	20
**Statistical Test**	two-tailed unpaired parametric t-test	
**t-value**	3.541	
**df**	37	
**Significance**	**, 0.0011	

Figure 8h	Ctrl	Dag1 gRNA
	108.2802053	59.68530047
	50.64075326	176.8284962
	118.207772	76.21474932
	60.19776909	45.2346832
	89.424501	21.84822956
	111.1759058	72.73580492
	29.81628505	41.95094409
	51.90787139	57.73432754
	28.36054598	61.36599194
	113.8104703	28.80612369
	38.73290899	27.30958785
	67.52041058	50.1498934
	50.85524441	39.12495601
	99.83105502	57.4950924
	123.9068941	50.97900089
	85.99441837	55.35536807
	118.1043532	87.35778537
	62.24492551	60.39623466
	66.79484883	32.19995023
		52.06958718
**Mean**	77.6740599	57.74210535
**Number (# cells)**	19	20
**Statistical Test**	two-tailed unpaired parametric t-test	
**t-value**	1.92	
**df**	37	
**Significance**	0.0626	

Figure 8j

Ctrl	cell 1	cell 2	cell 3	cell 4	cell 5	cell 6	cell 7	cell 8	cell 9	cell 10	cell 11	cell 12	Mean	Number (# cells)
5uA	0.031474609	0.043486328	0	0	0.007141113	0.091515175	0.110262047	0.047985542	0.0693806	0.129817307	0.097531204	0	0.052382827	12
10uA	0.02628418	0.060776367	0	0	0.037841797	0.102711395	0.982356384	0.127104965	0.253462631	0.186468216	0.060016911	0.151181503	0.165683696	12
20uA	0.035904594	0.101010742	0.052040501	0.054248131	0.376037598	2.772379883	1.50570459	0.482328857	0.482195587	0.721340271	0.208338303	0.931259888	0.643565745	12
30uA	0.088775635	0.130922852	0.071965996	0.202257965	1.59128418	2.128801514	1.320086182	0.675057739	0.831288513	3.239874023	0.275560333	1.029508789	0.965448643	12
40uA	0.106132813	0.536416016	0.116586914	0.516092224	2.050795898	3.60232	1.296279541	0.836994446	0.929600586	3.419802734	0.413389618	1.160182495	1.248716107	12
50uA	0.126616211	0.52152832	1.983847656	0.737582275	1.85279541	3.417922852	1.379556519	0.925656189	1.232713623	3.205703369	0.375696686	1.530207031	1.440818845	12
75uA	0.798700684	0.499174683	2.975693848	0.914426209	3.00335498	2.548973389	1.677492432	1.299780273	1.250039185	3.287775391	0.461017212	1.487925171	1.683696121	12
100uA	0.951189819	0.897519531	3.212612305	1.243427368	2.836173584	1.672636475	1.8892323	1.73614502	1.34569458	3.255654297	0.731093994	2.374923096	1.845525197	12

Dag1 gRNA	cell 1	cell 2	cell 3	cell 4	cell 5	cell 6	cell 7	cell 8	cell 9	cell 10	cell 11	cell 12	cell 13	cell 14	cell 15	Mean	Number (# cells)	Significance (compared to ctrl)
5uA	0	0	0	0	0	0	0.032287598	0.01167977	0.06951458	0.118286133	0.056191048	0.052246094	0.129148041	0.037920784	0.088005516	0.039685304	15	>0.9999
10uA	0	0	0.031463068	0.08427639	0	0.019917151	0	0.050070885	0.886066711	0.290036072	0.415649414	0.052716511	0.173754181	0.035330109	0.232666016	0.1514631	15	>0.9999
20uA	0.112419334	0.00982307	0.058144531	0.195040802	0.279845001	0.157300873	0.0346143	0.078462486	0.958475281	0.365189728	0.817521545	0.146025864	0.376779999	0.238518204	0.276079956	0.273616065	15	>0.9999
30uA	0.05980703	0.021563362	0.20378418	0.233376892	0.585796265	0.161785629	0.051740974	0.10731823	2.584055908	0.616752808	0.770276794	0.830465149	0.366377625	0.273796539	0.343096558	0.480666263	15	0.5697
40uA	0.049901844	0.110265396	0.100991211	0.167736572	0.618221497	0.339723541	0.125036621	0.127388435	2.28573999	0.472150116	1.277739502	0.848197937	0.407491821	0.361313751	0.401812378	0.512914041	15	0.0516
50uA	0.123102135	0.112563187	0.100058594	0.306545685	0.699374329	0.654956055	0.105578613	0.187721115	2.395504639	0.489929199	1.3316521	1.092434082	0.690214294	0.49766629	0.484059052	0.618090625	15	* 0.019
75uA	0.294210999	0.350881714	0.325490723	0.74230957	0.643015564	1.003979492	0.409556061	0.219996018	2.643435547	1.019368591	1.129436768	1.295481689	0.997099	0.942955017	0.764099121	0.852087725	15	* 0.0171
100uA	0.333569489	0.483326477	0.742670898	0.87916394	0.6121922	1.281112671	0.525480164	0.484514343	2.103018311	1.33657019	1.549746582	1.293406738	1.026224121	1.134314941	0.716937866	1.033483262	15	* 0.0216

Statistical Test: Two-way ANOVA with Bonferroni Multiple comparisons test at each intensity (ctrl vs. Dag1 sgRNA: p value<0.0001)

Figure 8k	Ctrl	Dag1 gRNA
	10.62550475	3.645751918
	8.805873242	5.390839994
	40.39377718	6.746950138
	14.10199631	9.256741527
	33.73833846	6.696902324
	15.96151562	14.417346
	12.80473751	5.701350381
	17.16271428	4.313186436
	13.37820452	27.85725361
	34.98470153	11.75106217
	6.413722686	13.02248327
	21.96850698	14.88843904
		10.24650226
		12.17131082
		6.826420951
**Mean**	19.19496609	10.19550272
**Number (# cells)**	12	15
**Statistical Test**	two-tailed unpaired parametric t-test	
**t-value**	2.665	
**df**	25	
**Significance**	* 0.0133	

Figure 8l	Ctrl	Dag1 gRNA
	0.307302003	0.444587741
	0.371998124	0.347425134
	0.146834551	0.457595003
	0.153129044	0.188288923
	0.197922961	0.389014145
	0.151253648	0.735129968
	0.167889583	0.60624117
	0.320445735	0.465523295
	0.094573964	0.118239269
	0.142643303	0.358275495
	0.397936578	0.330202692
	0.252114904	0.221660146
		0.161286348
		0.169566094
		0.612903308
**Mean**	0.225337033	0.373729249
**Number (# cells)**	12	15
**Statistical Test**	two-tailed unpaired parametric t-test	
**t-value**	2.499	
**df**	25	
**Significance**	* 0.0194	

Figure 8n

Ctrl	cell 1	cell 2	cell 3	cell 4	cell 5	cell 6	cell 7	cell 8	cell 9	cell 10	cell 11	cell 12	Mean	Number (# cells)
PPR 20ms	0.631132638	0.553751999	0.897062675	1.241378573	0.667080217	0.552956731	0.580330664	0.601166998	0.539317135	0.734770715	0.981567309	0.791583981	0.731008303	12
PPR 50ms	0.753242573	0.69213681	1.044903825	0.888412017	0.850612881	0.596307637	0.7419849	0.54745353	0.332142049	0.829316616	1.090596408	0.758465759	0.760464584	12
PPR 100ms	0.991041593	0.850460542	1.182192124	0.912071185	0.837344827	0.773359063	0.557769256	0.534545484	0.498983611	0.808577581	1.238880522	0.718261539	0.82529061	12
PPR 500ms	0.564586598	0.699284282	0.942433935	0.92916978	0.831414369	0.429989857	0.785126866	0.801652688	0.565272	0.77850737	1.16701019	0.849577446	0.778668782	12

Dag1 gRNA	cell 1	cell 2	cell 3	cell 4	cell 5	cell 6	cell 7	cell 8	cell 9	cell 10	cell 11	cell 12	cell 13	cell 14	cell 15	Mean	Number (# cells)	Significance (compared to ctrl)
PPR 20ms	0.591979003	1.321339575	3.251087449	0.769447564	1.015054539	2.377504499	2.335096941	0.530300319	0.439970328	1.210002502	1.401069664	1.515203471	0.935971177	1.058561348	0.535322794	1.285860745	15	* 0.0213
PPR 50ms	0.707593675	1.317858115	0.762419007	0.706948044	0.659447105	3.221664611	1.866368252	0.58561396	0.663184244	1.119577301	1.749416521	1.0621295	0.681301703	0.641521118	1.288656271	1.135579962	15	0.228
PPR 100ms	0.764808599	0.952697823	0.561193754	0.553718418	0.975345869	2.757802322	2.47220728	0.49935488	0.731149454	1.124975777	0.952675808	0.873160422	0.886924609	0.583607209	0.976273481	1.044393047	15	>0.9999
PPR 500ms	1.009533104	0.934917856	0.745162369	0.63645839	0.999617417	1.457476853	0.532195917	0.51496537	0.739616839	1.121869649	1.02740597	0.878485018	0.678342627	0.530561623	0.878964459	0.845704897	15	>0.9999

Statistical Test: Two-way ANOVA with Bonferroni Multiple comparisons test at each PPR interval (ctrl vs. Dag1 sgRNA: **, p value=0.0024)

[illegible]	Gephyrin Puncta Density, Avg. per Animal			
	Dendrite		Soma	
	Ctrl	Dag1 gRNA	Ctrl	Dag1 gRNA
	1.526869	0.843597	0.154881	0.104939
	1.311606	1.858334	0.130709	0.221581
	2.246503	1.536325	0.205448	0.210425
	1.417114	1.365429	0.154499	0.202417
Mean	[illegible]	[illegible]	[illegible]	[illegible]
Number (# animals)	[illegible]	[illegible]	[illegible]	[illegible]
Statistical Test	[illegible]		[illegible]	
t-value	[illegible]		[illegible]	
df	[illegible]		[illegible]	
Significance	[illegible]		[illegible]	

[illegible]	Gephyrin Puncta Size Avg. per Animal			
	Dendrite		Soma	
	Ctrl	Dag1 gRNA	Ctrl	Dag1 gRNA
	0.165403	0.142322	0.162902	0.141129
	0.15703	0.178171	0.125497	0.125952
	0.155251	0.156403	0.14149	0.133431
	0.16161	0.160925	0.145249	0.136866
Mean	[illegible]	[illegible]	[illegible]	[illegible]
Number (# animals)	[illegible]	[illegible]	[illegible]	[illegible]
Statistical Test	[illegible]		[illegible]	
t-value	[illegible]		[illegible]	
df	[illegible]		[illegible]	
Significance	[illegible]		[illegible]	

[illegible]

Two-way ANOVA with Bonferroni Multiple comparisons test at each intensity (ctrl vs. Dag1 sgRNA: **, p value=0.0013)

[illegible]

Two-way ANOVA with Bonferroni Multiple comparisons test at each PPR interval (ctrl vs. Dag1 sgRNA: **, p value=0.0011)

[illegible]

Figure S1a	dCre	dCre + Nrxn3a (-SS4)	dCre + Nrxn3a (+SS4)	dCre + Nrxn3b (+SS4)	Cre	Cre + Nrxn3a (-SS4)	Cre + Nrxn3a (+SS4)	Cre + Nrxn3b (+SS4)
	0.184179813	0.132523391	0.112809039	0.212469641	0.362849313	0.014300614	0.170201797	0.008244272
	0.516034031	0.108467477	1.144635125	0.009742867	0.080754898	0.079138828	0.350381156	0.001572201
	0.153269688	0.285304313	0.346378938	0.207753125	0.498088313	0.056131234	0.140267266	0.001122983
	0.216897078	0.634393688	0.144387875	0.344235125	0.071051156	0.130748305	0.12140775	
	0.57171525	0.117355289	0.228069563	0.030268994	0.280509563	0.823991438	0.218572672	
		0.333540031						
	0.067119234		0.212084625	0.007029649	0.006869941	0.028232646	0.048105672	0.018294398
	0.018991566	0.435695531	0.010184222	0.269547469	0.006312526	0.028103434	0.205653672	0.006640425
	0.055050047	0.037417738	0.54872475	0.007917137	0.039493953	0.147968641	0.126755148	0.000350974
	0.096679422	0.128275039	0.074563742	0.033583039		0.01272389	0.073041789	0.009193352
	0.011834082	0.132610688		0.0222705	0.007688306			
						0.213402984	0.169983125	0.020588008
	0.830609125		0.381527656	0.048641852	0.207010906	0.183987828	0.425984125	0.015814412
	0.604184188	0.182053906	0.616769	0.432661813	0.019592262	0.905159625	0.185564109	0.005912603
	0.592879	0.976778563	0.118442094	0.039742832	0.025494992	0.329058531	0.685742	0.006224247
	0.056895133	0.458283625	0.292397188	0.085202234	0.075105211	0.230089094		0.087138047
	0.291643156	0.508208563	0.466116156	0.01397306	0.104828328			
		0.398557063			0.045287148			
	0.149115984							
	0.131776859							
	0.330084938							
	0.296013063							
	0.19267625							
	0.360642188							
	0.165656266							
	0.298955125							
	0.076521531							
	0.106366188							
	0.142602891							
	0.493508188							
	0.845217875							
	0.030788553							
	0.6855145							
**Average**	0.285780707	0.324630994	0.335506427	0.117669289	0.122062454	0.227359792	0.224743099	0.015091327
**Count**	30	15	14	15	15	14	13	12
**Statistical Test**	One-way ANOVA with Dunnett's multiple comparison test							
**Significance**		ns	ns	ns	ns	ns	ns	**, p = 0.0031

Figure S1b	dCre	dCre + Nrxn3a (-SS4)	dCre + Nrxn3a (+SS4)	dCre + Nrxn3b (+SS4)	Cre	Cre + Nrxn3a (-SS4)	Cre + Nrxn3a (+SS4)	Cre + Nrxn3b (+SS4)
	0.172900516	0.147628859	0.426429719	0.052199926	0.08994532	0.02735673	0.130763734	0.064350621
	0.138945453	0.166570484	0.05748875	0.119348359	0.05754648	0.057297309	0.027245523	0.300385938
	0.041784023	0.125379234	0.189357438	0.335354813	0.088014336	0.117863961	0.118510492	0.028194094
	0.04824059	0.117460977	0.202081781	0.097453203	0.04153818	0.020971979	0.031355678	0.037774453
		0.07175875	0.242168141	0.323941625				
	0.187374406				0.415725156	0.294442344	0.082677281	0.175065125
	0.199416328	0.176805844	0.123195039	0.029904594	0.360621406	0.175970672	0.189741234	0.202216656
	0.123923672	0.046846746	0.064127355	0.063642313	0.132333594	0.066926719	0.08895307	0.265621063
	0.081788984	0.166277516	0.019694625	0.153851578	0.100311016	0.407120156	0.144947547	0.077962063
	0.068353898	0.079766102	0.049177723	0.060529426	0.398671438	0.135114094	0.122186297	0.292406656
		0.055760875	0.062459543	0.124969148				
	0.057336391				0.582446	0.151896438	0.218407688	0.211084203
	0.131578859	0.166019234	0.435618969	0.37423725	0.191710422	0.337513125	0.3809835	0.113531352
	0.184666969	0.232899328	0.354688281	0.457417	0.150926484	0.063308051	0.026606115	0.247742641
	0.320672406	0.094221266	0.112987695	0.185119484	0.164241969	0.174373344	0.112115219	0.162228141
	0.351822875	0.076578414	0.3335195		0.132110516		0.118401977	0.155037438
	0.129770547							
	0.136397719							
	0.127870883							
	0.187718547							
	0.0647005							
	0.124405094							
	0.04485525							
	0.172780594							
	0.159539969							
	0.399236969							
	0.400889625							
	0.09124343							
	0.303677719							
	0.312901688							
**Average**	0.170171211	0.123140974	0.190928183	0.182920671	0.207581594	0.156165763	0.128063954	0.166685746
**Count**	28	14	14	13	14	13	14	14
**Statistical Test**	One-way ANOVA with Dunnett's multiple comparison test							
**Significance**		ns	ns	ns	ns	ns	ns	ns

	IPSC Charge	
**Figure S1c**	CTRL	Nrxn3b (+SS4)
	0.345903063	0.091930125
	0.461169031	0.019823619
	0.415922969	0.050931902
	0.379047906	0.092001266
	0.279923063	0.036017352
	0.160254672	0.054837168
	0.13295325	0.279817156
	0.845059875	0.108485586
	0.878630938	0.010681768
	0.981140125	0.087839922
	0.59734275	0.448897281
	0.646609313	0.371157719
	0.139396469	0.148149531
	0.498109188	0.087313555
	0.014579151	0.232181156
		0.059043805
	0.327678656	
	0.189382266	0.205759859
	0.193190828	0.33160725
	0.162173516	0.080424234
		0.411683875
	0.536585063	0.148563109
	0.09031875	0.024800535
	1.33266325	0.342029406
		1.180190125
**Average**	0.436728822	0.204340304
**Count**	22	24
**Statistical Test**	Unpaired two-tailed t-test with Welch's correction	
**t value**	2.661	
**df**	38.52	
**Significance**	*, p = 0.0113	

	NMDA EPSC Charge	
**Figure S1d**	CTRL	Nrxn3b (+SS4)
	0.183872766	0.033696805
	0.143652656	0.130113109
	0.869577875	1.1204615
	0.158806313	0.165253875
	0.07700025	0.193441578
	0.370902344	0.031689832
	0.04082391	0.2752975
	0.091058281	0.5264295
	0.210610375	0.089020219
	0.457153906	0.134514359
	0.109111625	
	0.146066547	0.046663695
	0.374963	0.508864
	0.08757918	0.069019078
	0.057311289	0.304277375
	0.302646469	0.223863734
**Average**	0.230071049	0.256840411
**Count**	16	15
**Statistical Test**	Unpaired two-tailed t-test with Welch's correction	
**t value**	0.2953	
**df**	25.29	
**Significance**	ns, p = 0.7701	

	mIPSC Frequency	
**Figure S1f**	dCre	Cre
	1.066666667	0.920833333
	4.404166667	1.333333333
	2.8	5.3375
	3.595833333	0.175
	2.125	0.591666667
	0.441666667	1.354166667
	4.5875	2.245833333
	3.4125	0.9625
	6.179166667	6.291666667
	0.195833333	0.208333333
	3.070833333	0.029166667
	0.158333333	0.95
	6.883333333	1.220833333
	6.733333333	0.270833333
		0.32
**Average**	3.261011905	1.480777778
**Count**	14	15
**Statistical Test**	Unpaired two-tailed t-test with Welch's correction	
**t value**	2.266	
**df**	24.94	
**Significance**	*, p = 0.0324	

	mIPSC Amplitude	
	dCre	Cre
	48.751831	49.422012
	61.820564	36.538712
	88.837051	41.517723
	42.643044	30.685257
	44.222057	67.094864
	35.121231	39.904263
	43.812778	41.958763
	43.69558	63.694691
	48.555325	100.563744
	36.982784	56.726528
	49.005009	37.738255
	69.158958	43.307274
	57.806011	38.840649
	56.243481	34.165604
		51.941872
**Average**	51.90397886	48.94001407
**Count**	14	15
**Statistical Test**	Unpaired two-tailed t-test with Welch's correction	
**t value**	0.4972	
**df**	26.4	
**Significance**	ns, p = 0.6231	

	mIPSC Decay	
**Figure S1g**	dCre	Cre
	18.173332	15.151967
	22.525948	18.832672
	16.075632	17.478884
	18.973219	16.758999
	19.06813	14.715192
	21.991556	17.967175
	17.552132	16.986338
	17.163748	16.26474
	14.805205	14.888712
	22.883045	15.110542
	17.639212	14.107884
	18.637833	20.572859
	14.077933	18.734268
	19.648577	22.746523
		22.894957
**Average**	18.515393	17.54744747
**Count**	14	15
**Statistical Test**	Unpaired two-tailed t-test with Welch's correction	
**t value**	0.9556	
**df**	26.99	
**Significance**	ns, p = 0.3478	

	mIPSC Rise Time	
	dCre	Cre
	1.616022	1.614003
	1.829803	1.476859
	1.680236	1.748508
	2.439566	1.581552
	2.004077	1.400808
	1.604385	1.682388
	1.252414	1.737455
	1.40623	1.561611
	1.193044	1.224215
	1.376423	1.004603
	1.291497	1.72433
	2.336378	1.911399
	1.197355	1.605655
	1.62662	2.077952
		2.544178
**Average**	1.632432143	1.659701067
**Count**	14	15
**Statistical Test**	Unpaired two-tailed t-test with Welch's correction	
**t value**	0.1931	
**df**	26.08	
**Significance**	ns, p = 0.8484	

	mIPSC Frequency				
**Figure S1i**	dCre	Cre	Cre + Nrx3aSS4-	Cre + Nrx3aSS4+	Cre + Nrx3bSS4+
	0.570833333	0.358333333	0.2	0.295833	0.2
	7.2	0.320833333	2.8375	0.054167	0.07
	3.945833333	0.255	7.216666667	0.8125	0.06
	2.725	0.045833333	0.058333333	2.608333	0.49
	1.9125	0.275	4.2125	3.2375	0.09
	0.1625	0.041666667	0.15	0.454167	0.05
	0.641666667	0.204166667	3.008333333	0.1125	0.14
	0.566666667	0.595833333	0.829166667	0.3625	0.07
	0.570833333	1.683333333	0.429166667	0.141667	0.25
	0.4625		1.816666667	6.833333	0.08
			0.695833333	0.620833	0.64
			0.716666667	7.666667	0.05
			2.825	0.141667	0.22
			0.333333333	0.120833	0.12
**Average**	1.875833333	0.42	1.809226191	1.675892857	0.180714286
**Count**	10	9	14	14	14
**Statistical Test**	One Way ANOVA with Dunnett's multiple comparison test				
**Significance**		ns	ns	ns	ns, p = 0.0896

	mIPSC Amplitude				
	dCre	Cre	Cre + Nrx3aSS4-	Cre + Nrx3aSS4+	Cre + Nrx3bSS4+
	68.406891	31.184708	44.639023	45.35992	41.786243
	69.406616	34.419247	25.583582	42.29163	31.004263
	40.533661	38.884315	34.789028	41.41488	45.898438
	39.219593	32.891167	30.63868	58.35344	102.891937
	39.119232	42.073158	62.892738	27.53627	62.03949
	37.118416	41.205513	36.449669	62.96078	38.717251
	36.787994	59.7328	37.52206	35.07386	82.46727
	66.117928	51.417019	41.911488	28.83073	43.207306
	69.323166	39.025906	36.64677	30.77688	70.832428
	41.315117		21.116858	37.58856	37.231445
			46.370068	42.64996	54.191418
			33.363914	44.26833	32.927685
			36.190269	50.66516	62.716232
			46.132404	35.86108	49.559578
**Average**	50.7348614	41.20375922	38.16046793	41.68796286	53.96221314
**Count**	10	9	14	14	14
**Statistical Test**	One Way ANOVA with Dunnett's multiple comparison test				
**Significance**		ns	ns	ns	ns

	mIPSC Frequency			
**Figure S1k**	dCre	dCre + Nrx3b	Cre	Cre + Nrx3b
	6.929166667	0.0125	1.025	0.041666667
	0.129166667	0.204166667	0.445833333	0.00833
	1.495833333	0.033333333	0.520833333	0.379166667
	0.258333333	0.020833333	0.2625	0.0125
	0.220833333	0.020833333	0.229166667	0.104166667
				0.116666667
	0.770833333	0.070833333	7.4	4.016666667
	0.158333333	0.075	0.495833333	0.1125
	0.125	0.045833333	0.65	0.145833333
	0.516666667	0.054166667		0.433333333
	7.15	0.025		0.070833333
	0.15	1.091666667	2.945833333	0.070833333
	7.033333333	0.154166667	2.2	0.020833333
	3.208333333	0.141666667	0.095833333	0.9
	1.020833333	0.020833333	2.025	0.6
	0.2	0.204166667	0.566666667	1.791666667
	1.966666667		0.120833333	0.1
	1.179166667		1.0625	0.0625
	2.170833333		0.016666667	
			0.175	
	1.333333333	0.175	0.308333333	0.320833333
	1.1125	0.158333333	0.120833333	0.0625
	1.3375	0.133333333	0.0625	0.0875
	0.620833333	0.095833333	0.1	0.204166667
	1.191666667	1.6625	0.804166667	0.091666667
	0.554166667	0.045833333	0.675	0.029166667
	2.108333333	0.354166667	0.433333333	0.2875
	0.416666667	0.055555556	0.7	0.104166667
	1.729166667	0.5375	0.854166667	0.0375
	0.175	0.15	0.761111111	2.195833333
**Average**	1.616517857	0.221722222	0.928034979	0.443154643
**Count**	28	25	27	28
**Statistical Test**	One Way ANOVA with Dunnett's multiple comparison test			
**Significance**		**, p = 0.0010	ns, p = 0.16	**, p = 0.0050

	mIPSC Amplitude			
	dCre	dCre + Nrx3b	Cre	Cre + Nrx3b
	48.634289	35.760788	56.514019	37.94294
	47.018448	46.348991	45.16227	22.181919
	42.691334	50.619942	47.068779	98.941917
	33.716438	43.30706	56.619312	36.975677
	43.266853	50.896343	39.934429	42.347935
				50.206398
	44.884575	50.16531	37.290131	48.431755
	63.584927	47.911606	44.299431	33.759766
	34.38699	42.314598	46.982208	23.689777
	67.863716	34.84375	33.455906	28.23571
	38.708496	90.018135	51.713654	76.088631
	82.831108		26.189554	32.86845
	59.320034		41.295479	34.262695
	44.322674		30.285645	
			60.292854	
	36.887436	66.17897	35.407978	35.532604
	37.795624	59.617855	33.84631	36.429039
	60.960144	68.044281	58.382164	43.51981
	65.956314	39.219597	33.117676	34.586754
	61.870441	30.416801	33.921886	37.760788
	70.900391	36.52644	38.5275	62.271248
	59.208473	44.038662	29.142906	55.937965
	44.213867	62.184052	31.560902	33.835018
	140.107025	41.741035	34.241688	30.497631
	39.92503	65.222847	29.468266	42.500969
**Average**	55.17628813	50.26885315	40.61337279	42.55675635
**Count**	23	20	24	23
**Statistical Test**	One Way ANOVA with Dunnett's multiple comparison test			
**Significance**		ns	*, p = 0.0112	*, p = 0.035

Figure S2b

Measure	Group	Average	Count	Statistical Test	t value	df	Significance
Homer1 Puncta Density, Avg. Per Culture	ΔCre	0.24881683	3	Unpaired two-tailed t test with Welch's correction	0.7312	3.48	ns, p = 0.5108
	Cre	0.27336084	3				
Homer1 Puncta Density, Pseudoreplicate	ΔCre	0.24794782	52	Unpaired two-tailed t test with Welch's correction	0.9198	103	ns, p = 0.3598
	Cre	0.27022857	53				
Gephyrin Puncta Density, Avg. Culture	ΔCre	0.16671195	3	Unpaired two-tailed t test with Welch's correction	0.7542	3.32	ns, p = 0.5006
	Cre	0.20634746	3				
VGAT Puncta Density, Avg. Culture	ΔCre	0.13838428	3	Unpaired two-tailed t test with Welch's correction	0.4023	2.37	ns, p = 0.7210
	Cre	0.15533396	3				
Gephyrin Puncta Density, Pseudoreplicate	ΔCre	0.16340959	52	Unpaired two-tailed t test with Welch's correction	1.561	102	ns, p = 0.1215
	Cre	0.20122159	53				
VGAT Puncta Density, Pseudoreplicate	ΔCre	0.13715154	52	Unpaired two-tailed t test with Welch's correction	0.7546	103	ns, p = 0.4522
	Cre	0.15211393	53				

Figure S2c

Measure	Group	Average	Count	Statistical Test	t value	df	Significance
Gephyrin Puncta Size, Avg. Per Culture	ΔCre	0.31400272	3	Unpaired two-tailed t test with Welch's correction	0.1738	2.59	ns, p = 0.8747
	Cre	0.32696911	3				
Gephyrin Puncta Size, Pseudoreplicate	ΔCre	0.31826817	50	Unpaired two-tailed t test with Welch's correction	1.526	81	ns, p = 0.1308
	Cre	0.26956691	50				
Homer1 Puncta Size, Avg. Per Culture	ΔCre	0.32313954	3	Unpaired two-tailed t test with Welch's correction	1.515	3.97	ns, p = 0.2049
	Cre	0.40541289	3				
Homer1 Puncta Size, Pseudoreplicate	ΔCre	0.32365402	52	Unpaired two-tailed t test with Welch's correction	1.517	98.5	ns, p = 0.1325
	Cre	0.36320083	50				
vGAT Puncta Size, Avg. Per Culture	ΔCre	0.9531134	3	Unpaired two-tailed t test with Welch's correction	0.2931	2.81	ns, p = 0.7898
	Cre	0.99083033	3				
vGAT Puncta Size, Pseudoreplicate	ΔCre	0.94376789	48	Unpaired two-tailed t test with Welch's correction	0.4875	96	ns, p = 0.6270
	Cre	0.9961794	50				

Figure S2d

Measure	Group	Average	Count	Statistical Test	t value	df	Significance
Gephyrin Intensity, Avg. Per Culture	ΔCre	1011.764	3	Unpaired two-tailed t test with Welch's correction	0.4463	3.94	ns, p = 0.6788
	Cre	916.486627	3				
Gephyrin Intensity, Pseudoreplicate	ΔCre	1022.44286	50	Unpaired two-tailed t test with Welch's correction	1.769	88	ns, p = 0.0804
	Cre	915.826333	51				
Homer1 Intensity, Avg. Per Culture	ΔCre	349.91021	3	Unpaired two-tailed t test with Welch's correction	0.9807	2.6	ns, p = 0.4090
	Cre	238.115522	3				
Homer1 Intensity, Pseudoreplicate	ΔCre	350.628942	52	Unpaired two-tailed t test with Welch's correction	4.221	69.1	****, p < 0.0001
	Cre	234.342469	52				
vGAT Intensity, Avg. Per Culture	ΔCre	437.540301	3	Unpaired two-tailed t test with Welch's correction	0.7519	2.86	ns, p = 0.5091
	Cre	529.271862	3				
vGAT Intensity, Pseudoreplicate	ΔCre	440.881528	48	Unpaired two-tailed t test with Welch's correction	2.391	84.2	*, p = 0.019
	Cre	540.198931	50				

Figure S2e

Measure	Group	Subset	Average	Count	Statistical Test	t value	df	Significance
sGABAARα1 Levels (dendrite) - avg. per culture	dCre	MAP2	100	3				
	dCre	VGAT having MAP2	100	3				
	dCre	VGAT Having MAP2 and Homer1	100	3				
	dCre	VGAT having MAP2 not Homer1	100	3				
	Cre	MAP2	115.749777	3	Unpaired two-tailed t test with Welch's correction	0.8615	2	ns, p = 0.479
	Cre	VGAT having MAP2	122.12411	3	Unpaired two-tailed t test with Welch's correction	0.6816	2	ns, p = 0.565
	Cre	VGAT Having MAP2 and Homer1	131.991967	3	Unpaired two-tailed t test with Welch's correction	1.293	2	ns, p = 0.325
	Cre	VGAT having MAP2 not Homer1	121.36309	3	Unpaired two-tailed t test with Welch's correction	0.4509	2	ns, p = 0.696
sGABAARα1 Levels (dendrite) - Pseudoreplicate	dCre	MAP2	100.594785	57				
	dCre	VGAT having MAP2	100.000003	45				
	dCre	VGAT Having MAP2 and Homer1	100.000003	34				
	dCre	VGAT having MAP2 not Homer1	100.000005	34				
	Cre	MAP2	114.518198	56	Unpaired two-tailed t test with Welch's correction	0.9217	109	ns, p = 0.359
	Cre	VGAT having MAP2	119.555093	39	Unpaired two-tailed t test with Welch's correction	1.02	67.1	ns, p = 0.312
	Cre	VGAT Having MAP2 and Homer1	130.814886	29	Unpaired two-tailed t test with Welch's correction	1.66	51.2	ns, p = 0.103
	Cre	VGAT having MAP2 not Homer1	118.401944	32	Unpaired two-tailed t test with Welch's correction	0.6605	53.7	ns, p = 0.512
sGABAARα1 Levels (soma) - avg. per culture	dCre	MAP2	100	3				
	dCre	VGAT having MAP2	100	3				
	dCre	VGAT Having MAP2 and Homer1	100	3				
	dCre	VGAT having MAP2 not Homer1	100	3				
	Cre	MAP2	112.13388	3	Unpaired two-tailed t test with Welch's correction	0.5815	2	ns, p = 0.620
	Cre	VGAT having MAP2	96.8248833	3	Unpaired two-tailed t test with Welch's correction	0.1835	2	ns, p = 0.871
	Cre	VGAT Having MAP2 and Homer1	101.815373	3	Unpaired two-tailed t test with Welch's correction	0.1092	2	ns, p = 0.923
	Cre	VGAT having MAP2 not Homer1	91.3450433	3	Unpaired two-tailed t test with Welch's correction	0.7899	2	ns, p = 0.512
sGABAARα1 Levels (soma) - Pseudoreplicate	dCre	MAP2	99.999997	27				
	dCre	VGAT having MAP2	99.9999984	25				
	dCre	VGAT Having MAP2 and Homer1	99.9999942	24				
	dCre	VGAT having MAP2 not Homer1	100.000005	23				
	Cre	MAP2	113.088468	28	Unpaired two-tailed t test with Welch's correction	0.7947	50.1	ns, p = 0.431
	Cre	VGAT having MAP2	98.9792696	27	Unpaired two-tailed t test with Welch's correction	0.07471	48.2	ns, p = 0.941
	Cre	VGAT Having MAP2 and Homer1	106.22976	23	Unpaired two-tailed t test with Welch's correction	0.4491	39.8	ns, p = 0.656
	Cre	VGAT having MAP2 not Homer1	92.7500644	27	Unpaired two-tailed t test with Welch's correction	0.5025	47.7	ns, p = 0.618

Figure S2f

Measure	Group	Subset	Average	Count	Statistical Test	t value	df	Significance
sGABAARα2 Levels (dendrite) - avg. per culture	dCre	MAP2	100	3				
	dCre	VGAT having MAP2	100	3				
	dCre	VGAT Having MAP2 and Homer1	100	3				
	dCre	VGAT having MAP2 not Homer1	100	3				
	Cre	MAP2	104.282557	3	Unpaired two-tailed t test with Welch's correction	1.151	2	ns, p = 0.369
	Cre	VGAT having MAP2	105.393733	3	Unpaired two-tailed t test with Welch's correction	0.846	2	ns, p = 0.487
	Cre	VGAT Having MAP2 and Homer1	122.09297	3	Unpaired two-tailed t test with Welch's correction	1.675	2	ns, p = 0.236
	Cre	VGAT having MAP2 not Homer1	90.07392	3	Unpaired two-tailed t test with Welch's correction	1.427	2	ns, p = 0.290
sGABAARα2 Levels (dendrite) - Pseudoreplicate	dCre	MAP2	100.000001	76				
	dCre	VGAT having MAP2	100.000001	58				
	dCre	VGAT Having MAP2 and Homer1	100.000006	49				
	dCre	VGAT having MAP2 not Homer1	100	48				
	Cre	MAP2	103.822964	75	Unpaired two-tailed t test with Welch's correction	0.3729	148	ns, p = 0.709
	Cre	VGAT having MAP2	103.736805	54	Unpaired two-tailed t test with Welch's correction	0.3268	108	ns, p = 0.744
	Cre	VGAT Having MAP2 and Homer1	114.308909	41	Unpaired two-tailed t test with Welch's correction	0.9845	80.9	ns, p = 0.328
	Cre	VGAT having MAP2 not Homer1	89.6259029	39	Unpaired two-tailed t test with Welch's correction	0.7867	83.9	ns, p = 0.434
sGABAARα2 Levels (soma) - avg. per culture	dCre	MAP2	100	3				
	dCre	VGAT having MAP2	100	3				
	dCre	VGAT Having MAP2 and Homer1	100	3				
	dCre	VGAT having MAP2 not Homer1	100	3				
	Cre	MAP2	85.6978567	3	Unpaired two-tailed t test with Welch's correction	1.429	2	ns, p = 0.289
	Cre	VGAT having MAP2	72.0811567	3	Unpaired two-tailed t test with Welch's correction	3.043	2	ns, p = 0.0932
	Cre	VGAT Having MAP2 and Homer1	75.24247	3	Unpaired two-tailed t test with Welch's correction	2.24	2	ns, p = 0.154
	Cre	VGAT having MAP2 not Homer1	80.7741667	3	Unpaired two-tailed t test with Welch's correction	1.596	2	ns, p = 0.252
sGABAARα2 Levels (soma) - Pseudoreplicate	dCre	MAP2	100.000003	28				
	dCre	VGAT having MAP2	100.000003	27				
	dCre	VGAT Having MAP2 and Homer1	99.9999969	26				
	dCre	VGAT having MAP2 not Homer1	100.000001	27				
	Cre	MAP2	84.5754022	27	Unpaired two-tailed t test with Welch's correction	1.304	49.9	ns, p = 0.198
	Cre	VGAT having MAP2	85.94671	24	Unpaired two-tailed t test with Welch's correction	1.072	46.6	ns, p = 0.289
	Cre	VGAT Having MAP2 and Homer1	74.5364591	22	Unpaired two-tailed t test with Welch's correction	2.28	44.5	*, p = 0.027
	Cre	VGAT having MAP2 not Homer1	81.5467432	22	Unpaired two-tailed t test with Welch's correction	1.07	45.5	ns, p = 0.290

Figure S2g

Measure	Group	Subset	Average	Count	Statistical Test	t value	df	Significance
sGABAARγ2 Levels (dendrite) - avg. per culture	dCre	MAP2	100	3				
	dCre	VGAT having MAP2	100	3				
	dCre	VGAT Having MAP2 and Homer1	100	3				
	dCre	VGAT having MAP2 not Homer1	100	3				
	Cre	MAP2	124.4462	3	Unpaired two-tailed t test with Welch's correction	1.54	2	ns, p = 0.263
	Cre	VGAT having MAP2	105.40633	3	Unpaired two-tailed t test with Welch's correction	0.3675	2	ns, p = 0.749
	Cre	VGAT Having MAP2 and Homer1	106.427923	3	Unpaired two-tailed t test with Welch's correction	0.7329	2	ns, p = 0.540
	Cre	VGAT having MAP2 not Homer1	110.580983	3	Unpaired two-tailed t test with Welch's correction	0.4325	2	ns, p = 0.708
sGABAARγ2 Levels (dendrite) - Pseudoreplicate	dCre	MAP2	100.000001	79				
	dCre	VGAT having MAP2	99.9999959	63				
	dCre	VGAT Having MAP2 and Homer1	100	53				
	dCre	VGAT having MAP2 not Homer1	99.9999982	46				
	Cre	MAP2	120.501637	80	Unpaired two-tailed t test with Welch's correction	2.273	146	*, p = 0.0245
	Cre	VGAT having MAP2	101.073728	60	Unpaired two-tailed t test with Welch's correction	0.09844	107	ns, p = 0.922
	Cre	VGAT Having MAP2 and Homer1	106.018144	49	Unpaired two-tailed t test with Welch's correction	0.5892	99	ns, p = 0.557
	Cre	VGAT having MAP2 not Homer1	103.280849	43	Unpaired two-tailed t test with Welch's correction	0.1697	67	ns, p = 0.866
sGABAARγ2 Levels (soma) - avg. per culture	dCre	MAP2	100	3				
	dCre	VGAT having MAP2	100	3				
	dCre	VGAT Having MAP2 and Homer1	100	3				
	dCre	VGAT having MAP2 not Homer1	100	3				
	Cre	MAP2	94.9104033	3	Unpaired two-tailed t test with Welch's correction	0.3617	2	ns, p = 0.752
	Cre	VGAT having MAP2	102.290537	3	Unpaired two-tailed t test with Welch's correction	0.4586	2	ns, p = 0.692
	Cre	VGAT Having MAP2 and Homer1	97.0766167	3	Unpaired two-tailed t test with Welch's correction	0.4543	2	ns, p = 0.694
	Cre	VGAT having MAP2 not Homer1	89.11749	3	Unpaired two-tailed t test with Welch's correction	1.516	2	ns, p = 0.269
sGABAARγ2 Levels (soma) - Pseudoreplicate	dCre	MAP2	99.9999989	28				
	dCre	VGAT having MAP2	99.9999958	26				
	dCre	VGAT Having MAP2 and Homer1	100	24				
	dCre	VGAT having MAP2 not Homer1	96.8714383	23				
	Cre	MAP2	94.3679886	28	Unpaired two-tailed t test with Welch's correction	0.553	53.9	ns, p = 0.583
	Cre	VGAT having MAP2	98.6372868	28	Unpaired two-tailed t test with Welch's correction	0.116	47.6	ns, p = 0.908
	Cre	VGAT Having MAP2 and Homer1	93.411447	27	Unpaired two-tailed t test with Welch's correction	0.5532	46.1	ns, p = 0.583
	Cre	VGAT having MAP2 not Homer1	88.9673048	25	Unpaired two-tailed t test with Welch's correction	0.6853	41.4	ns, p = 0.497

0.307481	0.276499295<br>0.291358	0.258972697<br>0.255357	0.021505376<br>0.183657	0.56437734<br>0.281842	0.368867946<br>0.589185	0.149035043<br>0.293594	0.213919441<br>0.264988	0.04663056<br>0.282916	0.046274954<br>0.429099		0.154294383<br>0.095740502	0.094633182<br>0.310001322	0.266671027<br>0.400655204	0.260745793<br>0.340232618	0.0937053<br>0.409690552	0.18168294<br>0.25700832	0.371928313<br>0.265715832	0.255735147<br>0.413804736	
0.015124	0.280199205<br>0.50012	0.151371052<br>0.276715	0.188838192<br>0.231865	0.077294686<br>0.258235	0.049892778<br>0.428033	0.138950195<br>0.380999	0.205276814<br>0.194397	0.151766004<br>0.077784	0.02392917<br>0.193594		0.409549817<br>0	0<br>0.158363839	0.198393655<br>0.409984233	0.736085363<br>0.269470292	0.275062546<br>0.237236394	0.043773255<br>0.374201974	0.349170954<br>0.315633147	0.334397881<br>0.271585421	
0	0.045945325<br>0.203209	0.147772852<br>0.11951	0<br>0.050034	0.115942029<br>0.149607	0.049892778<br>0.091385	0.035495156<br>0.23774	0.278968978<br>0.086622	0.165582914<br>0.138918	0.095716679<br>0.193594		0.076941397<br>0.109662411	0<br>0.04940646	0.138042341<br>0.027446137	0.479124769<br>0.191137256	0.204539326<br>0.050305959	0<br>0.074607711	0.377948501<br>0.18930216	0.253577796<br>0	
0.3	0.13333333<br>0.387251	0.105<br>0.139481	0.09611111<br>0.320833	0.755<br>0.280513	0.545<br>0.242222	0.26<br>0.255731	0.90833333<br>0.587708	0.60727273<br>0.12625	0.73<br>0.337273		0.30259259	0.312777778	0.20816667<br>0.221766382	0.32088889<br>2.669*	0.20714286<br>0.398944444	0.095<br>0.352047619	0.30722222<br>0.351904762	0.32175<br>0.153333333	
0.431389	0.20247619<br>0.4165	0.14166667<br>0.412778	0.1<br>0.652857	0.52159091<br>0.438283	0.32930556<br>1.909*	0.29125<br>0.237778	0.24611111<br>0.535417	0.17<br>0.223333	0.26<br>0.394848		0.38111111<br>0.156666667	0.09833333<br>0.4535	0.19111111<br>0.429	0.198<br>0.329191919	0.27666667<br>0.341474359	0.215625<br>0.52765873	0.4115<br>0.207555556	0.2375<br>0.482222222	
	0.56<br>1.044444	1.07714286<br>2.243667	1.4125	0.10833333<br>1.161667	1.13<br>2.477	1.695<br>1.876	2.54938889<br>0.365	1.785<br>0.540625	1.1175<br>0.674545		1.64333333<br>0.766	0.9	0.62416667<br>0.385	1.31314815<br>1.23	1.14033333<br>0.385	1.59375	1.08611111<br>0.757142857	0.84944444	
807.61	594.8133	556.9133	1048.155	1008.16	673.1667	479.4<br>584.3567	509.07<br>604.745	535.1933<br>510.26	1645.54<br>537.76	889.33	566.67	947.005	886.54	604.18		649.4667	743.1566667	588.1067<br>739.83	802.1<br>868.29
187.6633333	283.2967<br>211.5667	237.4033<br>182.6967	230.38<br>305.53	255.5367<br>139.6233	262.6033<br>157.4167	228.335<br>129.61	239.7267<br>206.23	263.3<br>217.85	241.735<br>156.79		313.2133<br>152.3333333	247.9367<br>230.2366667	230.4367<br>299.495	298.72<br>167.36	323.4033<br>229.8966667	247.54<br>236.0633333	283.7367<br>181.1933333	243.885<br>219.9266667	
	266.26<br>322.8967	656.44<br>401.18	531.765	139.65<br>480.445	267.94<br>771.02	490.39<br>509.28	433.5167<br>243.55	286.87<br>298.24	366.68<br>232.67		466.07<br>296.555	400.87	180.695<br>186	405.2167<br>369.735	453.93<br>141.58	261.205	478.3933<br>201.52	336.6733	

33.48021	12.2502	63.34532	
42.33584	34.76389	65.59058	79.18105
41.91633	33.61175	58.37682	
36.64892		100.06	107.9747
39.44164	43.93734		
105.0972			
115.9903			
82.4835			

39.16337	42.11664	93.33142	49.09946	21.34694	19.13479	39.46503	19.93921	14.83709	39.12985	45.91157	64.66649	82.74361	68.91204	140.5837	202.2075	106.7496	96.00538	55.70986	77.15363
102.6496	185.8874	55.05906	10.14853	22.74097	38.40238	39.24474	19.55933	222.0497	110.0081	68.85602	89.65206	97.44521		213.8005		174.7543	82.98026	78.18902	2.296363
74.21718	198.0743	55.76811	5.474394	12.98871	40.25966	41.04813	21.78253		172.8461	73.92536	125.7399	101.9231		224.0573			76.91649	83.45434	
100.8319	174.8998	103.43	12.82897	45.78832	25.77273		2.999882	215.917	103.1326	33.25934	74.58439			199.8064		169.9278	84.52828	73.22532	2.232941
197.6082	234.2987	52.25383	61.55681	72.68835	206.5872	39.19316	57.79912	81.26746	50.73437	55.97427	64.97932	47.02509	46.48509	203.768	217.6554	226.3737			
	51.73256	73.02757	150.0926	223.0235					72.34349	87.70712	55.26295	10.70116	126.7131	134.1736	142.3665				
		84.09971	158.1738	233.272						101.0223	57.80241	11.1929	144.3124	135.8806					
	50.30377	46.75811	145.8272						70.34545	64.13965			89.08024	135.4007	138.4345				

51.51454	20.42581	25.89501	21.56196	27.10597	166.0047	233.9983	177.4256	187.2051	102.0075	110.6277	79.20016	30.69293	36.1586	23.95518	69.69614	112.711	120.5162	50.67563	113.8129	155.8579	
39.5808	19.90921	25.1273	12.46702	97.69895	137.7296	158.8966			59.4108	62.73111			62.75646	40.50909	134.8465	128.2788	147.2597		119.7309	223.5182	
37.97189	9.182969	33.06181	12.29081	105.8446	208.0786	156.6508			46.98307				65.83939	49.72859	198.0929	128.8458	145.7968		109.5178		
88.9494	26.04585	23.48384		60.31853	140.4888				195.6028	75.9399			48.93905	26.59047	39.99118	100.2598	26.94332		182.5204	270.5838	
80.13594	93.49063	102.6847	155.4389	55.91072	94.49498	63.81926	277.9352	166.4696	169.804	34.36024	45.14409	28.98267	179.331	164.9946	200.4338	98.77064	75.07372	119.4143	76.46259	34.8079	135.6905
		111.3921	130.9823				185.4306	149.7531	127.0463	49.45303	46.54458			97.49302	101.7194	196.2407	52.30444	87.07268	102.0711	50.22924	145.7041
		109.8177	129.5704				192.302	312.78	125.2507	48.75407	45.95857			120.3687	125.588	193.467	52.36791	83.72744	99.82262	63.80289	147.0025
			157.4104				195.9096	157.8208			54.04006			23.71396	79.57198		45.04617	127.3336	147.822	44.07199	157.372

**Datapoints**																												
	0.163413		0.215625		0.334817		0.385309	0.222681	0.174779	0.089002	0.094114	0.409507		0.211347		0.17702		0.389947		0.358869		0.281459		0.202268		0.420773	0.226879	
	0.231057		0.311105		0.204122		0.380168	0.248278	0.033033	0.144657	0.138926			0.153709		0.460853		0.226431		0.383818		0.375892		0.568858		0.371226	0.318739	
	0.030525		0.077071		0.331675		0.21384	0.299888	0.203724	0.155798	0.094114	0.25748		0.196138		0.194049		0.016205		0.177831		0.095702		0.115398		0.189794	0.117089	
	0.212193		0.238871		0.368235		0.41655	0.312506	0.071194	0.246185	0.169824	0.107502	0.308318		0.111649		0.219331		0.220612		0.207722		0.260198		0.229374			
	0.030525		0.077071		0.256593		0.055586	0.171737	0.152816	0.13359	0.260538	0.26899		0.20332		0.116347		0.162461		0.162932		0.054141		0.225407		0.249501	0.029551	
	0.111782		0.238814		0.355097		0.412873	0.264639	0.01908	0.257971	0.314812			0.348623		0.216294		0.254433		0.190546		0.278945		0.293681		0.182031	0.035746	
	0.135		0.49		0.4575		0.1525	0.233333	0.53	0.185	0.15	0.310833		0.136889		0.366667		0.04		0.229444		0.316667		0.438333		0.238889	0.096667	
	0.29		0.366603		0.165667		0.282159	0.315667	0.11	0.258889	0.139643			0.17		0.441333		0.088333		0.238667		0.47881		0.203667		0.351667	0.217333	
	0.226984		0.245556		0.358111		0.517013	0.35	0.620476	0.205	0.305	0.400714		0.391667		0.26		0.323286		0.532381		0.306417		0.248333		0.619206	0.406667	
	0.29619		0.260185		0.325556		0.35225	0.274762	0.99*	0.249444	0.281429			0.116667		0.60027		0.23		0.664194		0.552035		0.588121		0.338741	0.421944	
	0.56		0.6825		1.755		0.093333	1.059167	1.683333	0.953333	0.368889	1.247111		0.460476		1.05		0.2175		0.469333		0.9875		0.94075		0.647619	0.46	
	0.776		1.755635		1.246		1.713778	1.884214	0.53	0.821111	0.440741			0.8275		1.407778		0.568889		0.683778		1.148889		0.424196		0.935556	0.65	
757.93		500.47		638.06		661.565			1189.1	1662.78	1775.737	982.445	1322.7		1578.81		1304.323		1022.007		1018.797			1123.233		1704.703	850.87	1490.65
650.855		589.1333333		587.7166667		658.51			1036.807	1121.233	1272.12	1257.093	1114.077		1044.705		1254.47		980.575					1150.353		1281.95	908.1067	1386.643
	695.45		730.5633		839.31		534.5033	756.655	861.28	606.2367	544.7833	460.21		436.0433		390.1		577.655		421.71		495.27		350.1967		437.92	513.08	
	397.0633		263.0933		345.3567		406.5133	402.6167	372.465	410.56	290.485			272.95		234.7933		240.715		339.7633		204.65		225.35		379.95	347.4567	
	357.08		454.945		688.5633		176.41	345.305	791.72	403.04	213.18	278.2867		451.6467		505.31		266.27		386.575		479.375		341.05		334.7733	270.19	
	295.27		494.6833		634.4767		573.9267	925.3067	380.95	574.3633	398.4033			485.91		619.2733		339.53		496.5		524.2533		350.94		502.76	723.69	
**Datapoints**																												

1292.263	1217.25	1326.66	899.86
1513.347	1132.347	1284.8	1035.473

Figure S3b

Tissue	Nrxn1 SS4 Inclusion Replicate 1	Nrxn1 SS4 Inclusion Replicate 2
OB	71.7208675	73.4044161
Cx	40.3062898	41.1188425
Cer	94.3704878	94.1069557
BS	63.5638871	69.5460111
Eye	60.3800097	61.2033195
Hipp	40.826907	42.8999145

Tissue	Nrxn2 SS4 Inclusion Replicate 1	Nrxn2 SS4 Inclusion Replicate 2
OB	66.2033743	64.5215211
Cx	39.3448176	38.8608044
Cer	81.1493241	82.1305221
BS	52.1439123	51.4490076
Eye	53.6475466	53.6708987
Hipp	40.2637603	41.1503027

Tissue	Nrxn3SS4 Inclusion Replicate 1	Nrxn3SS4 Inclusion Replicate 2
OB	98.4275009	98.2556755
Cx	76.1719699	80.616479
Cer	99.0512897	98.9837387
BS	98.151548	98.3253956
Eye	52.6253965	36.476927
Hipp	55.0252365	50.3261549

Figure S3d

	Ctx		
Nrxn1+SS4	47.9969941	47.7335176	47.5698134
Nrxn2 +SS4	40.6625049	43.1159953	40.0250177
Nrxn3+SS4	76.8516497	78.4108513	75.755711

	Hipp		
Nrxn1+SS4	33.7922943	37.886894	36.777151
Nrxn2 +SS4	26.2854525	38.4577705	37.5427854
Nrxn3+SS4	48.9909823	56.023238	54.5863105

	OB			
Nrxn1+SS4	67.6337451	70.5328449	69.1133255	67.9828137
Nrxn2 +SS4	71.324379	75.625915	69.6024798	71.1623393
Nrxn3+SS4	91.2059826	94.8076694	93.8187561	83.5549349

Figure S3e

Cell #	Cell Type	Nt Transporter	Cell #	% Total
1	GC-1 (a)	vGAT	1914	8.801618688
2	GC-2 (b)	vGAT	3608	16.59155707
3	GC-3 (c)	vGAT	273	1.255403293
4	GC-4 (d)	vGAT	234	1.076059965
5	GC-5 (e)	vGAT	1669	7.674974708
6	GC-6 (f)	vGAT	237	1.089855606
7	GC-7 (g)	vGAT	679	3.122413317
8	PGC-1 (h)	vGAT	1037	4.768693093
9	PGC-2 (i)	vGAT	377	1.733652166
10	PGC-3 (j)	vGAT	279	1.282994574
11	EPL-IN (k)	vGAT	161	0.740366044
12	M/TC-1 (l)	vGLUT1	90	0.413869217
13	M/TC-2 (m)	vGLUT2	116	0.533431436
14	M/TC-3 (n)	vGLUT1	927	4.262852938
15	OSN (o)	n/a	1200	5.518256231
16	Transition (p)	n/a	2085	9.587970201
17	Immature (q)	n/a	3910	17.98031822
18	Astrocyte-Like (r)	n/a	2950	13.56571323
		Total	21746	

Cell Type	Nt Transporter	Cell #	% Total
GC-1 (a)	vGAT	1914	18.28429499
GC-2 (b)	vGAT	3608	34.46694689
GC-3 (c)	vGAT	273	2.607948032
GC-4 (d)	vGAT	234	2.235384028
GC-5 (e)	vGAT	1669	15.94382881
GC-6 (f)	vGAT	237	2.264042797
GC-7 (g)	vGAT	679	6.486434849
PGC-1 (h)	vGAT	1037	9.906381353
PGC-2 (i)	vGAT	377	3.601452044
PGC-3 (j)	vGAT	279	2.665265571
EPL-IN (k)	vGAT	161	1.538020634
	Total	10468	

% Total	Class
82.2888804	GC
16.17309897	PGC
1.538020634	EPL IN

Figure S3g

Nrxn1a	Nrxn1beta	Nrxn1gamma	Nrxn2alpha	Nrxn2b	Nrxn3a	Nrxn3b
71.12621854	99.1914017	124.191117	173.743031	98.8759267	182.511533	13.3611674
64.126373	86.0375944	101.486104	163.942845	99.734682	188.953559	18.1534874

Figure S3h

	Nrxn1+SS4		Nrxn2+SS4		Nrxn3+SS4	
VGAT-Cre x Ribotag (IP)	87.7867892	84.5185716	80.2605538	81.5170844	99.9998789	99.9998688
VGAT-Cre x Ribotag (Input)	75.8672742	77.7975766	68.0439768	70.835292	99.9998782	99.9998759

Figure S3i

aqp4	mbp	vgat	vglut1
1.32249151	2.5596993	216.064581	1.44791383
7.34345152	7.02640725	195.949886	8.31610714

Dag1-ex4/5	GRID1	GRID2	Nlgn1	Nlgn2	Nlgn3	LRRTM1	LRRTM2	LRRTM3	LRRTM4
74.55410775	79.1848698	13.262568	77.4629552	79.4991902	105.028284	10.6024128	113.412944	142.626714	110.915265
71.71963751	74.2983743	15.6517838	77.4660276	80.7797306	108.452086	18.6879369	95.7455801	132.564143	97.0570802

Cbln1	Cbln2	Cbln4	Nxphn1	Nxphn2	Nxphn3	Nxphn4
25.7730436	130.889255	2.9720944	151.823795	110.262673	89.4505852	43.5184137
28.9375895	116.048155	14.4385551	155.28374	116.068716	64.2376581	56.3486118

Car10	Car11	Fam19a1	Fam19a2	Fam19a4
91.7151355	46.4374026	2.96880737	81.8619855	31.934031
90.0590841	54.8881018	5.63161432	70.4320831	61.6198036

Sex Info	
Male	
Female	

Figure S4B

	Nrxn1+SS2a			Nrxn1+SS2ab		
OB	23.7997239	32.2456529	20.9447097	11.424269	4.28671274	0.23111404
Cx	38.6745243	34.7919661	32.6795186	3.12952473	9.70502156	0.63396085
Cer	14.7599742	16.1028898	3.62182863	4.62608225	6.03811695	0.71319345
BS	33.5629296	30.8352376	19.5077275	5.56943315	2.21983864	0.58499756
Eye	32.7050069	26.1400815	17.3548094	9.99992312	6.08933698	0.40834846
Hipp	29.1816702	22.8044389	10.1076601	4.30128743	6.1536954	0.52101004

	Nrxn2+SS2a			Nrxn2+SS2ab		
OB	28.5094542	24.9017565	16.8172125	2.99925581	2.51822949	0.19718005
Cx	50.2571349	48.4946611	51.9543239	4.67542608	3.93138537	1.02817131
Cer	51.2400945	49.4457019	55.9551432	2.59825706	2.57927569	0.45553619
BS	54.5110411	51.6679932	61.893745	8.65015149	8.44922265	3.65726614
Eye	53.6427778	48.8971785	60.364578	3.17026886	2.93660837	0.85465117
Hipp	45.567049	41.4261587	42.0220875	4.3938154	6.47958282	1.69988516

	Nrxn3+SS2a				Nrxn3+SS2ab			
OB	5.07276223	5.40128113	5.34709904	5.5790284	2.30849049	3.03799968	3.02814074	3.69268352
Cx	15.9258123	14.125159	12.8265741	12.9826657	3.53341664	3.38457514	3.34298999	4.52252604
Cer	8.11731176	6.52477303	9.66149714	6.03572067	3.64586348	3.25371096	4.30462194	2.58195832
BS	8.12651131	7.38369428	5.50860295	9.64217042	3.1772718	3.2702236	0	1.61424267
Eye	37.1758201	37.9784824	38.3735894	38.77649	3.67178176	2.9743369	5.48200332	3.54929969
Hipp	5.36066559	4.7700174	5.1153688	4.93079022	2.35652561	2.08082157	2.81958056	2.26591836

Figure S4D

		SS2a				SS2ab			
	Cx	39.9950667	42.1953469	39.0558421		0	0	0	
	Hipp	27.4875702	24.4343891	21.4373855		0	0	0	
Nrxn1	OB	57.2913284	64.5729304	56.8186383		0	0	0	
	Cx	63.0087959	62.9662996	61.9267294		11.422138	9.65968671	9.32448298	
	Hipp	73.376578	71.9248309	71.5079088		9.19924087	10.1919125	10.2652807	
Nrxn2	OB	41.5851435	47.9350129	46.3446934		7.0133218	6.70929239	8.26908011	
	Cx	3.63741736	4.09779417	3.27640856		0.35578707	0.30367704	0.67152621	
	Hipp	9.46532216	7.23930484	4.65554447		4.72962029	3.67867693	5.25117864	
Nrxn3	OB	13.081799	16.7218649	23.4350967	12.9042225	2.6628382	5.83591231	11.1126736	9.62748256

Figure S4F

Nrxn1 SS2	SS2a			SS2ab		
VGAT-Cre x Ribotag (IP)	7.58603292	6.57230977		0.60663137	2.44736908	
VGAT-Cre x Ribotag (Input)	25.1040962	21.9707598		1.11325543	1.70680497	
Tbet-Cre x Ribotag (IP)	63.7294833	60.4917908	56.5329429	2.3343465	2.34476108	5.52393185
Tbet-Cre x Ribotag (Input)	27.4689148	28.2129902	25.0341771	3.15083653	2.00808639	7.02504851

Nrxn2 SS2	SS2a			SS2ab		
VGAT-Cre x Ribotag (IP)	16.035355	14.9702996		0.843909	0.90710201	
VGAT-Cre x Ribotag (Input)	21.1556895	22.2662653		1.09516642	0.77430731	
Tbet-Cre x Ribotag (IP)	61.2515835	55.9792982	63.7814723	6.51525502	4.98458378	5.74364792
Tbet-Cre x Ribotag (Input)	23.199189	19.5961182	19.8181309	1.11452616	1.12829991	1.46705286

Nrxn2 SS3	SS2a			SS2ab		
VGAT-Cre x Ribotag (IP)	0.60080094	0.97665756		0.000072646	0.25730406	
VGAT-Cre x Ribotag (Input)	1.40220641	1.48190368		0.36261927	0.25765162	
Tbet-Cre x Ribotag (IP)	55.7089527	51.7598605	55.5424997	2.23131301	2.14231923	2.56996727
Tbet-Cre x Ribotag (Input)	1.48844967	1.40992792	1.41884026	0.11742332	0.37446783	0.19516724

Figure S4G

	dCre	Cre	Cre + Nrxn3a (+SS1, -SS2)	Cre + Nrxn3a (-SS1, -SS2)	Cre + Nrxn3a (-SS1, +SS2a)	Cre + Nrxn3a (-SS1, +SS2ab)	Cre + Nrxn3a (+SS1, +SS2a)	Cre + Nrxn3a (+SS1, +SS2ab)
	0.569891			0.706300375				
	0.233139063			0.204014531				
	0.03281327			0.55805				
	0.193254391							
		0.006841523	0.040388504	0.063349227		0.005818364		0.007813666
		0.086078438	0.96045525	0.04229241		0.020161061		8.0533E-05
		0.013472939	0.7800825	0.182568141		0.009070908		0.002347791
		0.074814945	0.001605872	0.007378239		0.00520351		0.147032438
		0.017514301	0.008813113			0.005669896		0.093342164
								0.004548723
		0.021170223	0.033911367	0.099012883		0.05311916	0.120276438	
		0.073130563	0.039751164	0.043083043	0.007759813		0.120164445	
		0.007764755	0.044591875	0.013076548	0.003369775	0.021059115	0.453669906	
		0.016584412	0.018136447	0.141026453	0.053152191	0.009508304	0.077901055	
		0.004835541	0.02511891	0.10703257	0.174748313	0.08465243	0.248176781	
		0.099819359	0.096631766	0.199760594	0.240849906	0.545518375	0.036356422	0.399405781
	0.093480844	0.023086189	0.026287072	0.094621078	0.828672438	0.016489037	0.032917965	0.034142141
	0.456857438	0.046879367	0.787552063	0.469979406	0.185947969	0.036249195	0.512565844	0.01941476
		0.097105961	0.438977313	0.633015188	0.415111406	0.115724367	0.450303906	
		0.143756578	0.240514094				0.427999344	
	0.068554484				0.181880328		0.090869625	0.047667004
	0.075162414				0.065542117		0.120187227	0.079569156
	0.074576875				0.205911922		0.100152695	0.014909358
	0.239198188							0.145043359
**Average**	0.203692796	0.048857006	0.236187821	0.222785043	0.214813289	0.071403363	0.214733973	0.076562836
**Count**	10	15	15	16	11	13	13	13
**Statistical Test**	One-Way ANOVA with Dunnett's Multiple Comparison Test							
**Significance**		ns	ns	ns	ns	ns	ns	ns

Figure S4H

	dCre	Cre	Cre + Nrxn3a (-SS2, +SS4)	Cre + Nrxn3a (+SS2a, +SS4)	Cre + Nrxn3a (+SS2ab, +SS4)
	0.228151266	0.270493969			
	0.632447375	0.081538258			
	0.469092063	0.07242107			
	0.304565438	0.28847425			
	0.312269188	0.321017563			
			0.095012641	0.793223313	0.038284688
			0.784338625	0.127359453	0.228408781
			0.174334047	0.040040902	0.248709281
				0.01543212	0.317301281
	0.164052672	0.091610852	0.60800125	0.1570415	0.117128031
	0.93577825	0.110595063	0.257874609	0.205395828	0.039205551
	0.389883969	0.624779813	0.536665375	0.158192047	0.099106039
	0.267138469	0.112728047	0.628825188	0.243786016	0.085628391
	0.490128125	0.162505703	0.598339	0.565154438	0.196270875
	0.409490438	0.030569123	0.243040156	0.16094575	
	0.316334	0.08349718	0.314777625	0.098338031	0.085651383
		0.02063374	0.341420406	0.174690344	0.149125688
		0.01376566	0.824854125	0.042296313	0.069020094
			1.030536938	0.097620594	
**Average**	0.409944271	0.163187878	0.495232307	0.205679761	0.139486674
**Count**	12	14	13	14	12
**Statistical Test**	One-Way ANOVA with Dunnett's Multiple Comparison Test				
**Significance**		*, p = 0.011		*, p = 0.0446	**, p = 0.0068

Figure S4I

	dCre	Cre	Cre + Nrxn3a (-SS4)	Cre + Nrxn3a (LNS2/3-only)	Cre + Nrxn3a (LNS2-only)
		0.006038114		0.759377938	0.349821031
		0.034301043		0.07197793	1.752929875
		0.215116219		0.98268975	0.232681375
				0.623020563	0.247843141
				0.081284563	0.528363688
	0.040933578	0.676100313	0.085394891	0.880997063	0.375117031
	0.253019375	0.016212763	0.545715375	0.099553203	0.485550125
	0.735948875	0.097722383	0.03950193	0.145887094	0.222493031
	0.278015594	0.065696508	0.252600188	0.185638109	0.477250938
	0.61814575	0.155046531	0.141472875	0.173300422	
			0.211748391		
			1.002428063		
		0.345421094	0.391278156		
		0.008918974	0.546738563		
		0.056377566	0.145824328		
		0.108022813	0.358434875		
		0.014229912	0.023247305		
		0.012300916			
		0.198396063			
		0.040120508			
		0.033272973			
		0.007997512			
	0.8344835	0.212273719	0.363734531	0.147021609	1.15941675
	0.080197617	0.039506512	0.160035563	0.295032406	0.329454063
	0.36258625	0.039967867	0.134588969	0.32206275	0.266610469
	0.276471594	0.110030922	0.330427188	0.129477984	0.252036391
	0.436331	0.167885297	1.32554575	1.046698125	0.995378063
	0.488727375	0.019573406	0.098544648		
	0.083844281	0.027915848	0.543393313		
	0.243237906	0.015381745	0.077077031		
	0.187276766	0.122543922	0.054908414		
		0.013174115	0.912878		
**Average**	0.351372819	0.102126627	0.352069016	0.396267967	0.548210426
**Count**	14	28	22	15	14
**Statistical Test**	One-Way ANOVA with Dunnett's Multiple Comparison Test				
**Significance**		*, p = 0.0469	ns	ns	ns

Figure S4j

	dCre	Cre	Cre + Nrxn3a (-SS4)	Cre + Nrxn3a (deltaLNS1-EGF2)	Cre + Nrxn3a (deltaLNS5-LNS6)
	0.70574725	0.025854367	0.293816031	0.008608752	0.248454188
	0.261348391	0.016649018	0.163300688	0.048268961	0.107842938
	0.044233617	0.693520375	0.112910531	0.05135275	0.018739975
	0.36153275	0.226495172	0.184953078	0.186158359	0.568767813
		0.029888117	0.486453906	0.003417466	0.905099813
	0.421234344	0.004150544	0.453952344		0.100832352
	0.402312125	0.040636957	0.070376109	0.030526645	0.210022797
	0.240020766	0.021392611	0.466697781	0.050977512	0.037917664
	0.318077313	0.015197372	0.8036285	0.076578188	0.418372375
	0.941805813	0.01180463	0.024431449	0.054586637	0.280105156
	0.039975055	0.022097689		0.037625414	0.529729438
	0.101985688	0.083755789		0.096436031	0.141273797
	0.372174594	0.124188		0.018898256	0.345108094
	0.336703781	0.466572063		0.301472969	1.024519188
	0.585802438	0.097374922		0.004514382	0.188632875
**Average**	0.366639566	0.125305175	0.306052042	0.069244451	0.341694564
**Count**	14	15	10	14	15
**Statistical Test**	One-Way ANOVA with Dunnett's Multiple Comparison Test				
**Significance**		*, p = 0.0207	ns	**, p = 0.0037	ns

Figure S4k

	dCre	Cre	Cre + Nrxn3a (LNS2-only, -SS2)	Cre + Nrxn3a (LNS2-only, +SS2a)	Cre + Nrxn3a (LNS2-only, +SS2ab)
	0.516451844	0.399643563	0.825810438	0.279778969	0.187477984
	0.31208925	0.380795719	1.023200563	0.395768688	0.357884938
	1.33785525	0.396974313	0.532168188	0.086468313	0.454155063
	0.554088875		0.305869969	0.476970688	0.178738656
	0.512526906		0.466617281	0.504745938	0.5852615
	1.266085	0.011399637	0.849412563	0.027186744	0.033822902
	0.219318391	0.141984906	0.210171234	0.379162031	0.023802578
	0.426461813	0.216048313	0.494751188	0.085967398	0.018461205
				0.094952336	0.252259063
				0.176041641	0.097650125
	0.211821766	0.00890393	0.089310445	0.018858912	0.109565125
	0.359874344	0.000566914	0.038812066	0.073612805	0.045932152
				0.055196316	0.096226516
				0.031795727	0.134679813
					0.066377906
**Average**	0.571657344	0.194539662	0.483612393	0.191893322	0.176153035
**Count**	10	8	10	14	15
**Statistical Test**	One-Way ANOVA with Dunnett's Multiple Comparison Test				
**Significance**		*, p = 0.0122	ns	**, p = 0.0032	**, p = 0.0018

Sex info
Male
Female

Figure S5b		Average	Count	Statistical Test	Significance
Gephyrin Size, Avg. Per Culture	dCre	0.207358906	5	One-Way ANOVA with Dunnett's Multiple Comparison Test	
	Cre	0.210395827	3		ns
	Cre + LNS2-SS2(-)	0.206538707	5		ns
	Cre + LNS2a/SS2ab	0.21191586	4		ns
Gephyrin Size, Pseudoreplicates	dCre	0.207020506	77	One-Way ANOVA with Dunnett's Multiple Comparison Test	
	Cre	0.210385812	44		ns
	Cre + LNS2-SS2(-)	0.206144217	75		ns
	Cre + LNS2a/SS2ab	0.211914099	57		ns
Synaptoporin Size, Avg. Per Culture	dCre	0.206043234	5	One-Way ANOVA with Dunnett's Multiple Comparison Test	
	Cre	0.209413227	3		ns
	Cre + LNS2-SS2(-)	0.213757592	5		ns
	Cre + LNS2a/SS2ab	0.19418001	4		ns
Synaptoporin Size, Pseudoreplicates	dCre	0.205999847	77	One-Way ANOVA with Dunnett's Multiple Comparison Test	
	Cre	0.209811725	44		ns
	Cre + LNS2-SS2(-)	0.213031978	75		ns
	Cre + LNS2a/SS2ab	0.19300512	57		*, p = 0.0490 (to dCre)

Sex-related data is in Source Data for Fig. 3

Figure S5c		Average	Count	Statistical Test	Significance
Capacitance	dCre	144.9868085	47	One-Way ANOVA with Tukey's Multiple Comparison Test (to dCre)	
	Cre	155.2536087	46		0.559
	Cre + LNS2-SS2(-)	158.2747727	44		0.208
	Cre + LNS2a/SS2ab	149.4508333	48		0.9024

Figure S5d		Average	Count	Statistical Test	Significance
Input Resistance	dCre	65.68085106	47	One-Way ANOVA with Tukey's Multiple Comparison Test (to dCre)	
	Cre	71.24130435	46		0.8789
	Cre + LNS2-SS2(-)	59.8	44		0.8641
	Cre + LNS2a/SS2ab	72.2875	48		0.8079

Figure S5e		Average	Count	Statistical Test	Significance
mIPSC rise time	dCre	1.442106167	18	One-Way ANOVA with Tukey's Multiple Comparison Test (to dCre)	
	Cre	1.510283	18		0.7491
	Cre + LNS2-SS2(-)	1.550786	18		0.3875
	Cre + LNS2a/SS2ab	1.598385611	18		0.1092
mIPSC decay time	dCre	6.662867944	18	One-Way ANOVA with Tukey's Multiple Comparison Test (to dCre)	
	Cre	7.255218444	18		0.0762
	Cre + LNS2-SS2(-)	6.708311444	18		0.9976
	Cre + LNS2a/SS2ab	7.078743389	18		0.319

Figure S5f

Male mIPSC Frequency			
ΔCre	Cre	Cre + Nom3n (LNS3-only, -SS2)	Cre + Nom3n (LNS3-only, +SS2)
	2.238584		3.280782
	2.825588		2.998381
	2.531171		5.656087
	1.931001		2.509022
	5.587882		3.14356
7.27338	1.321882	5.14865	
4.402726	1.081102	5.89494	
5.883589	5.729488	4.403482	
5.204376	6.150818	5.008673	
19.03614	5.440025	2.694169	
5.038824	2.558924	5.479813	
5.210758	2.402191	4.122657	
3.829075	0.683333	8.459068	
		7.697913	4.171934
		4.708068	3.80085
		10.81581	1.575429
		8.293754	0.88
		3.706399	1.958021
**Average** 6.98485975	3.098460692	5.879476615	2.9974066
**Count** 8	13	13	10
**Statistical test** One-Way ANOVA with Tukey's Multiple Comparison Test (to dCre)			
**Significance**	*, p = 0.0149 (to dCre)	ns, p = 0.8040	*, p = 0.0187 (to dCre)

Female mIPSC Frequency			
ΔCre	Cre	Cre + Nom3n (LNS3-only, -SS2)	Cre + Nom3n (LNS3-only, +SS2)
6.858381		5.691185	
3.157127		6.709773	
6.180941		11.09307	
5.694728		9.778805	
3.576517		3.209363	
			2.824992
			2.987387
			4.064391
			2.97506
			2.158424
			5.400035
			3.688889
			4.378105
17.42413	2.437031		
6.410996	4.50533		
11.74752	3.308155		
3.160973	2.197862		
4.803659	0.838285		
**Average** 6.9014972	2.6573206	7.2959992	3.559410375
**Count** 10	5	5	8
**Statistical test** One-Way ANOVA with Tukey's Multiple Comparison Test (to dCre)			
**Significance**	ns	ns	ns

0.183784163	0.211146081	0.197990092	0.213389571
0.213037779	0.200505051	0.200505051	0.183827376

0.191347639	0.193321859	0.194373065	0.164275921	0.188336806	0.160954392	0.174211864	0.197444353	0.182250618	0.18111001	0.174364583	0.186902486	0.194996232	0.200534591	0.215320357	0.201009615
0.215486908	0.213934859	0.209442478	0.236755824	0.229186047	0.226057143	0.22677685	0.220671296	0.224328969	0.20511542	0.2044898	0.203640974	0.1995316	0.204968553	0.189451396	
0.157251773	0.179982906	0.185318681	0.202928377	0.209718574	0.190882852	0.186388889	0.172352025	0.178948127	0.195550062	0.189519427	0.217258978	0.198884422	0.207254138	0.218145957	
0.210342531	0.225004625	0.225820567	0.229088748	0.216946939	0.219229358	0.219146552	0.211358457	0.204838113	0.23	0.242585445	0.221458154	0.229506276	0.215419847	0.22297911	

0.227182482	0.220769231	0.218337893	0.190242188	0.230773196	0.211110278	0.212663623	0.223508646	0.201063658	0.223184524	0.21497093	0.208955916	0.212822129	0.211447188	0.2054531
0.220650602	0.224528754	0.224761226	0.220357143	0.227048242	0.220988417	0.209338843	0.189812067	0.197096774	0.190194473	0.176787565	0.217193277	0.215473171	0.217480377	
0.210952782	0.206666667	0.220028736	0.2125	0.215171289	0.209294118	0.208047473	0.179861572	0.199334486	0.201447368	0.197527923	0.198761905	0.219418262	0.224573042	0.217428571
0.200193929	0.235520833	0.204694687	0.206590551	0.202112237	0.178521916	0.207699704	0.199395817	0.198898714	0.195043341	0.209765458	0.218315217	0.210599676		

0.188180645	0.172551963	0.180197368	0.17042836
0.180153584	0.171224784	0.171224784	0.153858521

0.219781086	0.221070111	0.23991433	0.195337972	0.204093264	0.179290123	0.191622912	0.222652361	0.217809216	0.206841629	0.215093046	0.208288557	0.238815094	0.209408503	0.233325021	0.22951278
0.250129496	0.258361732	0.21629572	0.251478473	0.246318519	0.235829139	0.216265625	0.197737478	0.241551155	0.236116208	0.214622568	0.2190131	0.233206997	0.242	0.234145937	
0.242482988	0.2116171	0.194854142	0.226491876	0.22160216	0.21064663	0.094924812	0.195298077	0.174471326	0.208226351	0.216481775	0.202898042	0.186978335	0.208804598	0.182697303	
0.202136259	0.200748848	0.172904074	0.159615946	0.16265	0.178213852	0.181423488	0.18230916	0.180159509	0.168958333	0.182369299	0.175629966	0.188361345	0.161885522	0.157037594	

0.197364729	0.213840231	0.225468393	0.248677966	0.245971429	0.222927046	0.218138862	0.255003549	0.227704778	0.213904939	0.196900085	0.189542254	0.219582367	0.243938607	0.207985797
0.18145749	0.216593977	0.233252427	0.204806825	0.207566702	0.220958549	0.172926094	0.162319392	0.179027084	0.1665337	0.201939328	0.190581798	0.193306773	0.155260736	
0.254768963	0.243642322	0.27783852	0.257278107	0.273060109	0.241460446	0.217329787	0.179385113	0.266998106	0.240016221	0.23863167	0.216965726	0.317393539	0.302068719	0.31992286
0.223445065	0.223763033	0.221958864	0.183446244	0.18133627	0.159663551	0.22243	0.224611973	0.200699774	0.208846154	0.246188159	0.273986063	0.250094178		

190.45	112.14	92.19
173.28	160.92	

109.91	183.45	140.74	108.18

27.7	87.9	134
31.9	38.2	

69.3	38.9	47.6	51.2

Sex info
Male
Female

Figure S6c	dCre	Cre
	90.47	143
	201.3	149.14
	145.51	140.95
	159.23	152.79
	142.2	114
	101.55	168.8
	151.71	184.18
	151.7	124
	120.62	104.26
	169.65	142.1
**Average**	143.394	142.322
**Count**	10	10
**Statistical Test**	two-tailed unpaired parametric t-test	
**t-value**	0.08383	
**df**	18	
**Significance**	0.9341	

Figure S6d	dCre	Cre
	89.7	50
	46.9	61.3
	75	84.1
	60.2	47.2
	37.2	101
	92.6	39.9
	84.1	42.5
	21.6	49
	134.2	70.8
	51.8	24.4
**Average**	69.33	57.02
**Count**	10	10
**Statistical Test**	two-tailed unpaired parametric t-test	
**t-value**	0.9775	
**df**	18	
**Significance**	0.3413	

Figure S6e	dCre	Cre								
	dCre									
5	0.01089	0	0	0	0.032111	0	0.009209	0	0	0
10	0.0875	0.013375	0.014564	0.027725	0.045074	0.089594	0.010154	0.070838	0.127337	0.317108
20	0.221874	0.19543	0.190428	0.23445	0.317175	0.230337	0.992836	0.234531	0.25199	0.085175
30	0.375998	0.519828	0.112331	0.338849	0.740318	0.177182	1.349891	1.096773	0.751633	0.448586
40	0.474511	0.535978	0.217489	2.825463	0.710385	0.486782	2.674438	1.136933	0.646271	0.645232
50	0.4363	0.597307	0.653354	2.581362	0.559172	0.277183	3.899458	0.975785	0.845971	0.862549
75	0.749729	0.979681	0.749522	2.48763	0.681496	0.843481	4.053519	1.192211	0.410324	1.249104
100	1.416992	1.077604	1.017433	2.474417	0.459214	1.172573	3.810618	1.345777	0.528076	1.469727

Cre									
0.047947617	0	0	0.066221	0	0.314133	0	0	0.162537	0
0.041718273	0	0.031687	0.065439	0	0.441561	0.078632	0	0.156475	0
0.131261826	0.023123	0.110056	0.123056	0.126434	1.776295	0.221509	0.483691	0.164514	0.053085
0.079971489	0.330688	0.307013	0.366446	1.03986	1.951182	0.799997	0.749635	0.193509	0.299162
0.105022324	0.310156	0.811421	0.791532	0.761496	1.687025	0.893887	2.197707	0.526049	0.854254
0.395803558	0.525275	0.908352	1.411771	1.027281	1.410661	0.736129	1.67798	0.407896	0.755643
0.787994385	1.309665	1.771461	1.560927	0.862454	1.376572	0.963636	2.134563	0.576843	1.34044
0.941862793	1.567593	1.652299	1.255493	1.367344	1.383663	0.99953	2.139377	0.735022	1.762381

dCre Average	Count	Cre Average	Count	Statistical Test	Significance
0.005221	10	0.059083862	10		>0.9999
0.0803269	10	0.081551227	10		>0.9999
0.2954226	10	0.321302483	10		>0.9999
0.5911389	10	0.611746349	10	Two-way ANOVA with Bonferroni Multiple comparisons test at each stimulus intensity (dCre vs. Cre: p value=0.5696)	>0.9999
1.0353482	10	0.893854932	10		>0.9999
1.1688441	10	0.925679156	10		>0.9999
1.3396697	10	1.268455539	10		>0.9999
1.4772431	10	1.380456479	10		>0.9999

Figure S6f	dCre	Cre
	13.22749	10.41863517
	12.00809	18.08214
	11.2438	20.83056
	31.67152	16.79896
	4.821623	13.67707
	11.872	7.044884
	47.03444	10.66727
	14.18007	25.33376
	4.46798	6.515135
	15.71852	19.87797
**Average**	16.6245533	14.92463842
**Count**	10	10
**Statistical Test**	two-tailed unpaired parametric t-test	
**t-value**	0.3716	
**df**	18	
**Significance**	0.7145	

	IPSC Rise		IPSC Decay	
Figure S6g	dCre	Cre	dCre	Cre
	1.491027	2.033070803	22.7901	12.76850033
	1.866502	1.032214	11.3062	12.9283
	2.26088	1.103509	12.8881	12.8529
	1.873203	1.527777	13.7093	13.4344
	1.248805	1.390082	11.2262	16.4224
	1.267272	1.010623	15.6446	11.2695
	0.996662	2.324509	8.82851	16.8104
	0.992544	1.298465	12.8376	15.3013
	2.511549	2.111774	14.6574	14.5118
	1.17677	1.454148	16.2029	15.5553
**Average**	1.5685214	1.52861718	14.009091	14.18548003
**Count**	10	10	10	10
**Statistical Test**	two-tailed unpaired parametric t-test		two-tailed unpaired parametric t-test	
**t-value**	0.1772		0.1326	
**df**	18		18	
**Significance**	0.8613		0.896	

	CV	
Figure S6h	dCre	Cre
	0.248219	0.3147226
	0.170912	0.240799
	0.361479	0.163016
	0.347907	0.326478
	0.324634	0.249392
	0.28849	0.336998
	0.144615	0.296107
	0.236748	0.154233
	0.293752	0.31052
	0.343822	0.186472
**Average**	0.2760578	0.25787376
**Count**	10	10
**Statistical Test**	two-tailed unpaired parametric t-test	
**t-value**	0.5622	
**df**	18	
**Significance**	0.5809	

	PPR									
Figure S6j	dCre	Cre								
	dCre									
20	0.55792	0.96763	1.659535	1.157485	0.728309	0.788017	0.634225	0.228243	0.531632	0.565501
80	0.753186	0.973997	1.22618	1.121064	0.840462	0.500409	0.687415	0.411656	0.748231	0.748638
200	0.732349	0.731142	1.1303	1.097689	0.977172	0.974008	0.707236	0.513644	0.677517	0.666455
500	0.708264	0.747334	1.084736	0.772938	0.567979	0.613974	0.90916	0.578462	0.59251	0.850125

Cre									
0.862600778	0.276107	0.88559	1.416255	0.157067	0.54282	0.503007	1.063717	1.246876	0.839456
1.175090869	0.357578	1.022193	0.801365	0.169173	0.491376	0.741858	0.716827	0.746138	0.702322
0.885651735	0.403566	0.69693	0.804243	0.430804	0.773668	0.570142	0.812441	0.692329	0.832703
0.923939793	0.697807	0.789211	0.40992	0.604853	0.830985	0.762673	1.07154	0.938677	0.648201

dCre Average	Count	Cre Average	Count	Statistical Test	Significance
0.7818497	10	0.779349578	10		>0.9999
0.8011238	10	0.692392087	10	Two-way ANOVA with Bonferroni Multiple comparisons test at each PPR interval (dCre vs. Cre: p value=0.9825)	>0.9999
0.8207512	10	0.690247774	10		>0.9999
0.7425482	10	0.767780679	10		>0.9999

Sex info

Male

Female

Figure S7b

	IPSC Amplitudes			
	dCre	Cre	Cre+Nrx3a (-SS4)	Cre+Nrxn3a (LNS2 only, -SS2)
	7100.361816	5601.376465	8167.047852	4633.089355
	3400.566162	512.750427	7921.791016	5784.296875
	7287.860352	1913.515381	4392.326172	7638.998535
	7099.714844	3860.51123	6635.75	13725.58106
	6195.54834	1827.140015	7623.226074	8490.084961
		3372.722168		
	8594.273438	5547.77832	6144.233887	5082.243164
	9789.188477	10210.83008	6835.422852	6057.451172
	9653.928711	3489.284912	4958.172363	5576.978027
	9398.688477	8772.75293	10662.46191	9709.303711
	6084.643555	8798.503906	9509.291016	8578.446289
		387.549469		
	8266.347656	4136.430176	6443.249023	12892.1377
	9672.782227	6046.296387	10620.83984	11023.78125
	11875.01172	3261.468262	12035.57031	10215.14063
	6497.924805	3676.184814	6442.977539	6030.330078
	8187.085449	8339.413086	5139.368164	9454.601563
**Average**	7940.261735	4691.441649	7568.781868	8326.164291
**Count**	15	17	15	15
**Statistical Test**	One-Way ANOVA with Dunnett's Multiple Comparison Test			
**Significance**		**, p = 0.0021	ns, p = 0.9595	ns, p = 0.9550

	IPSC Charge			
	dCre	Cre	Cre+Nrx3a (-SS4)	Cre+Nrxn3a (LNS2 only, -SS2)
	0.666118375	0.6605415	0.54348375	0.469371281
	0.218176641	0.033610895	1.012691625	0.472652906
	0.55118625	0.198168281	0.378314031	0.65822975
	0.921520438	0.567333625	0.385520375	1.016636688
	0.593207313	0.223788969	1.026108313	0.690181875
		0.5973115		
	1.1986435	0.731438688	0.461017063	0.563785625
	1.671587375	1.1720625	1.112642625	0.385512469
	2.451847	0.401769563	0.79151775	0.457701313
	0.949564813	1.295916625	1.046387	1.65468975
	1.55711325	0.484531156	1.440997625	1.35680075
		0.099865516		
	0.74435375	0.622409313	0.572935313	1.3647195
	1.626500625	0.68656725	1.481748	1.595212
	1.13426225	0.403042563	1.59697	2.13141775
	1.774306875	0.5456205	1.572208375	1.166836
	0.832968063	0.547506813	0.546745438	1.372983875
**Average**	1.126090434	0.545381486	0.931285819	1.023782102
**Count**	15	17	15	15
**Statistical Test**	One-Way ANOVA with Dunnett's Multiple Comparison Test			
**Significance**		**, p = 0.0035	ns, p = 0.5519	ns, p = 0.8877

			Average	Count	Statistical Test	Significance
Figure S7c		dCre	177.0909091	22		
		Cre	174.1818182	22	one-way ANOVA with Tukey's Multiple comparisons test	ns. 0.9964 (to ΔCre)
		Cre + LNS2-SS2(-)	190.5909091	22		ns. 0.7492 (to ΔCre)
Figure S7d		dCre	117.6363636	22		
		Cre	130.0454545	22	one-way ANOVA with Tukey's Multiple comparisons test	ns. 0.8670 (to ΔCre)
		Cre + LNS2-SS2(-)	116.0909091	22		ns. 0.9997 (to ΔCre)
Figure S7e	mIPSC rise time	dCre	0.959482364	22		
		Cre	1.102675864	22	one-way ANOVA with Tukey's Multiple comparisons test	ns. 0.1737 (to ΔCre)
		Cre + LNS2-SS2(-)	1.091553	22		ns. 0.2346 (to ΔCre)
	mIPSC decay time	dCre	6.539121	22		
		Cre	6.772319227	22	one-way ANOVA with Tukey's Multiple comparisons test	ns. 0.6028 (to ΔCre)
		Cre + LNS2-SS2(-)	6.408139773	22		ns. 0.8980 (to ΔCre)

Datepoints

142	246	174	173	162	173	223	177	117	102	109	208	193	181	200	116	165	156	143	321	223	192
182	195	115	183	138	169	147	179	127	107	119	129	224	227	198	236	223	216	205	211	186	116
160	200	270	220	202	181	215	186	97	109	127	201	161	190	239	227	270	115	206	226	193	198
101	117	174	71	112	90	58	82	117	244	127	69	78	69	91	298	146	127	128	72	113	104
125	105	211	102	99	71	193	108	261	197	88	126	155	113	69	98	67	69	73	213	121	197
74	81	57	68	115	177	73	143	79	219	225	47	182	136	104	93	118	145	57	68	103	190
0.751497	1.225913	1.274851	0.702076	0.930317	0.844572	0.773855	0.986055	0.614388	0.78582	0.977216	0.758013	0.85835	0.871558	0.955236	1.583391	0.939874	0.915021	0.822711	1.202824	1.224807	1.110267
1.321846	0.932409	1.284141	1.342092	0.996115	1.479891	1.152727	1.376135	1.224238	1.106856	0.961501	0.803839	0.822307	0.932223	0.824554	1.195627	1.081719	1.202099	1.203101	1.145357	1.035013	0.835079
0.88882	0.703754	0.915425	1.211389	1.118476	1.317294	1.151329	0.819173	0.992525	0.959567	1.207613	0.811904	1.050443	1.20025	0.941055	1.699967	1.538993	1.048288	0.964139	0.895861	1.244015	1.333886
6.538408	6.660049	6.742847	5.439456	7.069215	7.750745	6.649893	6.179838	5.039422	5.738066	6.155865	6.03246	7.377802	5.918706	5.693177	6.842042	7.977808	6.549384	6.317698	6.807586	6.921644	7.458551
6.314991	7.378869	6.615728	6.502527	5.728236	6.083956	6.826554	6.99345	6.566127	5.425073	7.022174	6.007778	6.785942	7.442647	6.696178	7.728466	6.56808	7.536832	7.54563	7.296455	6.521329	7.404001
5.864077	6.392012	7.416133	6.375872	7.299949	6.204538	6.604402	5.705038	7.046851	6.159317	6.60732	6.109668	5.45112	6.526221	6.172696	6.292316	5.773923	6.85868	5.968486	6.707858	6.627598	6.815

		P26-30		
	Sex Info	2 males, 1 female	female	female
**Figure S8e**	% Gephyrin with Dystroglycan (avg. per animal)	10"	20"	O/N
		36.4929513	26.5676373	5.69961144
		20.72548408		
		25.93236631		
	**Average**	27.7169339	26.5676373	5.69961144
	**Count**	3	1	1

**Figure S8f**		10"	20"	O/N
	Gephyrin Size with Dystroglycan (avg. per animal)	0.048988203	0.047410739	0.038281746
		0.043506626		
		0.049433426		
	**Average**	0.047309418	0.047410739	0.038281746
	**Count**	3	1	1

		10"	20"	O/N
	Gephyrin Size without Dystroglycan (avg. per animal)	0.027810297	0.017452976	0.017541977
		0.021896803		
		0.019119668		
	**Average**	0.022942256	0.017452976	0.017541977
	**Count**	3	1	1

		10"	20"	O/N
**Figure S8g**	Dystroglycan Puncta Size (avg. per animal)	0.023284233	0.015653814	0.009445584
		0.018735656		
		0.016995115		
	**Average**	0.019671668	0.015653814	0.009445584
	**Count**	3	1	1

Sex info
Male
Female

Figure S9b

Control	Dag1
[illegible]	[illegible]
[illegible]	[illegible]
[illegible]	[illegible]

	Control	Dag1
Average	99.88424077	39.92359333
Count	3	3
Statistical Test	Unpaired two-tailed t test with Welch's correction	
t value	14.1	
df	2.003	
Significance	**, 0.0050	

Figure S9c

Control	Dag1
[illegible]	[illegible]
[illegible]	[illegible]

	Control	Dag1
Average	100	33.44560325
Count	2	2

Figure	Measure	Group	Average	Count	Statistical Test	t value	df	Significance	Values
Figure S9d	Gephyrin Puncta Density, Pseudoreplicates	Control	0.447739559	39	Unpaired two-tailed t test with Welch's correction	0.3761	80.81	ns, p = 0.7079	0.467798122 [illegible]
		Dag1	0.452206552	44					0.51472015 [illegible]
Figure S9e	Gephyrin Puncta Size Pseudoreplicates	Control	0.182907849	39	Unpaired two-tailed t test with Welch's correction	2.04	73.77	*, p = 0.0450	0.18885895 [illegible]
		Dag1	0.191052932	44					0.202037 [illegible]
Figure S9g	Synaptoporin Puncta Density	Control	0.165334459	2	n/a				0.149231134 [illegible]
		Dag1	0.169178843	3					0.183930052 [illegible]
	Synaptoporin Puncta Density (pseudoreplicates)	Control	0.165334459	16	Unpaired two-tailed t test with Welch's correction	0.4576	34.14	ns, p = 0.6501	0.112388323 [illegible]
		Dag1	0.171118242	21					0.14140477 [illegible]
Figure S9h	Synaptoporin Puncta Size	Control	0.253430851	2	n/a				0.257096162 [illegible]
		Dag1	0.26840998	3					0.298219625; 0.246341116; 0.254669199
	Synaptoporin Puncta Size (pseudoreplicates)	Control	0.253430851	16	Unpaired two-tailed t test with Welch's correction	1.306	31.87	ns, p = 0.2008	0.229677791; 0.228842505; 0.233878924; 0.243077565; 0.266352531; 0.271148477; 0.280396825; 0.303394683; 0.278296672; 0.268403782; 0.250049645; 0.229674671; 0.247780767; 0.256979052; 0.230632672; 0.236307054
		Dag1	0.267525692	21					0.272551402; 0.289169909; 0.262481297; 0.242639257; 0.276772487; 0.29722031; 0.361761149; 0.383161189; 0.241898148; 0.236330532; 0.192685644; 0.279844413; 0.269735176; 0.266934175; 0.218232601; 0.265068238; 0.242876812; 0.257990431; 0.259391081; 0.25237467 [illegible]
Figure S9i	Synaptoporin Puncta Intensity	Control	404.803125	2	n/a				403.63625; 405.97
		Dag1	372.205	3					419.28125; 281.96375; 415.37
	Synaptoporin Puncta Intensity (pseudoreplicates)	Control	404.803125	16	Unpaired two-tailed t test with Welch's correction	2.433	28.77	*, p = 0.0214	385.05; 388.02; 384.13; 392.47; 409.4; 420.09; 419.12; 430.81; 432.9; 440.33; 423.77; 409.14; 430.51; 430.14; 341.78; 339.19
		Dag1	362.4766667	21					398.78; 404.93; 397.71; 385.68; 405.76; 423; 461.54; 476.85; 267.43; 265.27; 250.49; 280.57; 277.1; 270.9; 331.65; 312.3; 340.57; 412.05; 420.86; 412.92 [illegible]

Figure S9j

Control	Dag1
[illegible]	[illegible]

	Control	Dag1
Average	139.1165	144.9806667
Count	40	45
Statistical Test	two-tailed unpaired parametric t-test	
t-value	0.8349	
df	83	
Significance	0.4062	

Figure S9k

Control	Dag1
[illegible]	[illegible]

	Control	Dag1
Average	77.4525	69.77777778
Count	40	45
Statistical Test	two-tailed unpaired parametric t-test	
t-value	0.9214	
df	18	
Significance	0.3595	

Figure S9l

mIPSC rise time	
Control	Dag1
[illegible]	[illegible]

	Control	Dag1
Average	1.518910368	1.5804569
Count	19	20
Statistical Test	two-tailed unpaired parametric t-test	
t-value	0.8242	
df	37	
Significance	0.4151	

mIPSC decay time	
Control	Dag1
[illegible]	[illegible]

	Control	Dag1
Average	6.805479263	6.73531545
Count	19	20
Statistical Test	two-tailed unpaired parametric t-test	
t-value	0.2535	
df	37	
Significance	0.8013	

Figure S9m

Males mIPSC Frequency	
Control	Dag1
[illegible]	[illegible]

	Control	Dag1
Average	4.978880296	3.526124539
Count (cells)	13	13
Statistical Test	two-tailed unpaired parametric t-test	
t-value	2.003	
df	24	
Significance	ns, p = 0.0566	

Females mIPSC Frequency	
Control	Dag1
[illegible]	[illegible]

	Control	Dag1
Average	5.425656815	2.314907897
Count (cells)	6	7
Statistical Test	two-tailed unpaired parametric t-test	
t-value	3.537	
df	11	
Significance	**, p = 0.0047	

Figure S9n

sIPSC rise time	
Control	Dag1
[illegible]	[illegible]

	Control	Dag1
Average	1.520481333	1.984885733
Count	12	15
Statistical Test	two-tailed unpaired parametric t-test	
t-value	1.427	
df	25	
Significance	0.1658	

sIPSC decay time	
Control	Dag1
[illegible]	[illegible]

	Control	Dag1
Average	17.31828833	19.63672667
Count	12	15
Statistical Test	two-tailed unpaired parametric t-test	
t-value	0.9276	
df	25	
Significance	0.3625	

0.504489249	0.494811138	0.509428304	0.462860066	0.415233459	0.484271225	0.486035173	0.40253303	0.422289252	0.462860066	0.368829807	0.469151911	0.471679808	0.432872943	0.486496975
0.513661781	0.497433455	0.510839463	0.482501188	0.518316843	0.497433455	0.515778519	0.516484098	0.410999982	0.42511157	0.487555344	0.511294641	0.508369935	0.510784017	0.490377801
0.224181818	0.219400856	0.194639889	0.147439024	0.159328802	0.163191489	0.165753217	0.165872042	0.1632665	0.18242378	0.152487992	0.170844309	0.181540783	0.1800489	0.176272801
0.191635	0.176979	0.169282	0.202819	0.198871	0.182894	0.21515	0.213094	0.174034	0.169834	0.186404	0.189174	0.218105	0.215282	0.20641

Sex info
Male
Female

Figure S10a

Capacitance	
**Control**	**Dag1**
117.08	127.54
156.18	156.8
147.57	154.18
92.21	92.05
106.49	107.11
132.96	173.51
153.4	177.5
218.83	142.93
129.11	120.78
135.17	103.95
121.94	162.33
110.33	105.29
129.31	116.06
161.98	133.55
112.81	102.06
151.12	134.67
191.96	138.21
106.78	94.93
131.6	180.84
134.86	126.56
163.45	116.3
163.45	139.05
188.33	171.43
133.39	133.85
111.89	186.05
128.07	139.17
127.2	144.51
208.98	134.89
133.01	163.4
106.75	155.07
174.95	174.91
173.98	221.79
109.43	186.66
112.28	173.76
	145.17
	111.81
	189.9

**Average**	140.4955882	144.2856757
**Count (cells)**	34	37
**Statistical Test**	two-tailed unpaired parametric t-test	
**t-value**	0.5149	
**df**	69	
**Significance**	0.6083	

Figure S10b

Input Resistance	
**Control**	**Dag1**
37.8	59.9
52	44.8
68	63
269.9	278
151.5	52.5
64.5	51.1
41.3	32.8
40.8	62.2
65.1	122.4
55.4	150.8
62.4	57.6
64	88
50.1	67.9
45.9	59.8
114.1	110.9
49.7	66
34	54.4
86.1	312
43.2	62.4
54.7	27
41.5	65.3
49.5	58.5
36.7	48.3
85.9	52.8
39.9	35
49.1	48.8
43.4	42.1
62.9	24.9
61.2	43.7
39.1	58.6
57.3	44.7
53.3	28.6
158.7	26.1
52.4	43.7
	78.4
	109.9
	33

**Average**	67.1	72.05135135
**Count (cells)**	34	37
**Statistical Test**	two-tailed unpaired parametric t-test	
**t-value**	0.3848	
**df**	69	
**Significance**	0.7016	

Figure S10c

mIPSC rise time	
**Control**	**Dag1**
1.36242464	1.32606944
1.06889619	1.31985257
1.5886688	1.18278056
1.59762136	1.29420567
1.27107595	1.55337983
1.32968221	1.59595547
1.71774937	1.68084679
1.74856638	1.68246811
1.65593917	1.69154627
1.77788161	1.53539261
1.68446109	1.92323079
1.49660859	1.03380441
1.15113071	1.2810661
1.66807376	1.57791136
1.60285633	1.65525276
1.75172701	1.30984045
1.52859493	1.96221168
1.44444239	1.63610936
1.50708954	1.61372013
1.69732553	1.63807484
1.14297076	1.54313843
1.5057735	1.74680136
	1.85466447
	1.26828731

**Average**	1.513616355	1.537775449
**Count (cells)**	22	24
**Statistical Test**	two-tailed unpaired parametric t-test	
**t-value**	0.3625	
**df**	44	
**Significance**	0.7187	

mIPSC decay time	
**Control**	**Dag1**
5.742149	6.797866
5.884112	5.580808
6.534869	6.983007
7.109778	5.806882
7.139756	6.692733
5.800244	7.029226
7.114739	6.863265
6.375718	6.755925
7.103984	7.010672
6.900731	6.049423
6.30216	7.099854
7.659043	4.980918
6.021335	6.649791
6.954067	6.967265
8.174825	7.426681
7.073588	6.095699
5.992759	6.445709
5.44497	6.59809
6.873634	7.035208
6.672432	6.974571
4.29732	6.258906
6.000148	6.692184
	6.8094
	4.837061

**Average**	6.507834591	6.518381
**Count (cells)**	22	24
**Statistical Test**	two-tailed unpaired parametric t-test	
**t-value**	0.04763	
**df**	44	
**Significance**	0.9622	

Figure S10e

eIPSC rise time	
**Control**	**Dag1**
1.023431	0.924008
0.921237	2.047187
2.07507	1.951419
2.029818	0.826652
0.69137	0.91765
0.694102	1.424211
0.749382	1.005037
1.039092	1.212802
1.00596	0.934713
0.624128	0.790403
1.082952	2.13898
0.781017	0.929334
0.711506	0.862682
0.701869	1.198773
	1.183519
	0.838493
	0.821632

**Average**	1.009352429	1.176911471
**Count (cells)**	14	17
**Statistical Test**	two-tailed unpaired parametric t-test	
**t-value**	1.014	
**df**	29	
**Significance**	0.319	

eIPSC decay time	
**Control**	**Dag1**
14.38748	17.4509
13.38692	14.30824
14.2005	11.21818
16.44889	18.64396
12.18813	10.1811
11.20201	17.40168
10.30072	21.41401
15.59659	12.86009
13.85125	16.14735
11.92286	14.19951
11.38964	10.08974
9.277852	14.54645
9.745843	11.00477
8.657164	12.65497
	16.30113
	15.0603
	13.78862

**Average**	12.32541779	14.54535294
**Count (cells)**	14	17
**Statistical Test**	two-tailed unpaired parametric t-test	
**t-value**	2.18	
**df**	29	
**Significance**	0.0375	

Figure S10f

Capacitance	
**Control**	**Dag1**
136	196
190	195
152	199
159	118
131	170
127	144
172	101
161	118
166	163
98	202
97	90
151	161
146	138
262	172
181	228
150	171
251	187
246	192
122	215
161	187

**Average**	162.95	167.35
**Count (cells)**	20	20
**Statistical Test**	two-tailed unpaired parametric t-test	
**t-value**	0.3301	
**df**	38	
**Significance**	0.7431	

Figure S10g

Input Resistance	
**Control**	**Dag1**
140	58
97	76
130	74
189	132
118	79
130	69
160	135
122	107
98	143
207	162
218	153
82	96
196	229
52	89
125	98
182	191
65	82
51	72
189	98
126	157

**Average**	133.85	115
**Count (cells)**	20	20
**Statistical Test**	two-tailed unpaired parametric t-test	
**t-value**	1.227	
**df**	38	
**Significance**	0.2276	

Figure S10h

mIPSC rise time	
**Control**	**Dag1**
0.954207	0.857973
0.825172	0.902024
0.914136	0.607832
0.885057	0.925969
0.689429	0.956497
0.950228	1.087246
1.286087	1.090048
1.329153	1.054894
0.770715	0.903228
0.830757	0.947932
0.84176	0.79982
0.768451	1.116666
0.725824	1.045166
0.689776	0.818097
1.016896	1.230898
0.996834	1.181207
0.854601	1.138153
0.795974	0.691749
0.825165	1.255616
1.004295	1.248737

**Average**	0.89772585	0.9929876
**Count (cells)**	20	20
**Statistical Test**	two-tailed unpaired parametric t-test	
**t-value**	1.699	
**df**	38	
**Significance**	0.0976	

mIPSC decay time	
**Control**	**Dag1**
6.173596	7.341197
6.308409	7.230676
6.566475	7.036025
7.833928	8.120572
6.512726	7.73916
6.168713	7.67321
7.234847	6.526757
6.888544	8.345866
7.087439	7.85644
7.318935	7.189413
7.713615	7.784021
5.519385	7.089622
5.607076	7.42665
6.014364	7.31487
7.754037	6.787562
7.859223	7.913996
6.176741	7.487029
6.116704	6.810266
7.421533	7.88936
6.160111	6.988077

	6.72182005	7.42753845
	20	20
**Statistical Test**	two-tailed unpaired parametric t-test	
**t-value**	3.519	
**df**	38	
**Significance**	0.0011	
